# Identification of NAD interacting residues in proteins

**DOI:** 10.1186/1471-2105-11-160

**Published:** 2010-03-30

**Authors:** Hifzur R Ansari, Gajendra PS Raghava

**Affiliations:** 1 Institute of Microbial Technology, Sector 39A, Chandigarh, 160036, India

## Abstract

**Background:**

Small molecular cofactors or ligands play a crucial role in the proper functioning of cells. Accurate annotation of their target proteins and binding sites is required for the complete understanding of reaction mechanisms. Nicotinamide adenine dinucleotide (NAD^+ ^or NAD) is one of the most commonly used organic cofactors in living cells, which plays a critical role in cellular metabolism, storage and regulatory processes. In the past, several NAD binding proteins (NADBP) have been reported in the literature, which are responsible for a wide-range of activities in the cell. Attempts have been made to derive a rule for the binding of NAD^+ ^to its target proteins. However, so far an efficient model could not be derived due to the time consuming process of structure determination, and limitations of similarity based approaches. Thus a sequence and non-similarity based method is needed to characterize the NAD binding sites to help in the annotation. In this study attempts have been made to predict NAD binding proteins and their interacting residues (NIRs) from amino acid sequence using bioinformatics tools.

**Results:**

We extracted 1556 proteins chains from 555 NAD binding proteins whose structure is available in Protein Data Bank. Then we removed all redundant protein chains and finally obtained 195 non-redundant NAD binding protein chains, where no two chains have more than 40% sequence identity. In this study all models were developed and evaluated using five-fold cross validation technique on the above dataset of 195 NAD binding proteins. While certain type of residues are preferred (e.g. Gly, Tyr, Thr, His) in NAD interaction, residues like Ala, Glu, Leu, Lys are not preferred. A support vector machine (SVM) based method has been developed using various window lengths of amino acid sequence for predicting NAD interacting residues and obtained maximum Matthew's correlation coefficient (MCC) 0.47 with accuracy 74.13% at window length 17. We also developed a SVM based method using evolutionary information in the form of position specific scoring matrix (PSSM) and obtained maximum MCC 0.75 with accuracy 87.25%.

**Conclusion:**

For the first time a sequence-based method has been developed for the prediction of NAD binding proteins and their interacting residues, in the absence of any prior structural information. The present model will aid in the understanding of NAD^+ ^dependent mechanisms of action in the cell. To provide service to the scientific community, we have developed a user-friendly web server, which is available from URL http://www.imtech.res.in/raghava/nadbinder/.

## Background

All organisms posses small molecular weight cofactors or ligands which function in important metabolic and regulatory pathways. To understand the function and basic mechanism behind these ligands, proteins should be properly annotated. In the present scenario protein annotation has become a big challenge for computational biologists due to large gap between the genome and annotated proteome. Although manual annotation is the most accurate, it requires more expertise and time. On the other hand, automated rule based annotation is less reliable, but faster and provides more coverage [1]. Several years of enzyme annotation for different types of ligands led to many useful specialized databases and tools such as Catalytic Site Atlas [2], Macie [3], Procognate [4], Wssas [5] etc. These tools with other methods [6-8] can assign ligand binding sites in a protein if its structure or close homolog is known. Adenine-based dinucleotides such as nicotinamide adenine dinucleotide (NAD) and flavin adenine dinucleotide (FAD^+^) are some of the very important ligands as they are involved in pathways like glycolysis and photosynthesis. NAD^+ ^and its phosphorylated and reduced forms, NADP^+^, NADH and NADPH, have critical roles in cellular metabolism and energy production as hydride-accepting and hydride donating coenzymes. NAD^+ ^is not only coenzyme for oxidoreductases but also a substrate for three enzyme classes, namely, ADP-ribose transferases, cADP-ribose synthases and sirtuins (type III protein lysine deacetylases). NAD^+ ^level in the cell compartment are critical which is maintained by these enzymes. It has been observed that depletion in NAD^+ ^causes axanopathy leading to conditions like Alzheimers's disease (AD), Parkinson's disease and multiple sclerosis [9]. Because of their role, a number of proteins bind to these cofactors, collectively termed as NAD binding proteins (NADBP). NADBP are ubiquitous and found in archea, bacteria, and higher organisms including yeast. Some bacterial toxins like cholera (CT) exploit the mechanism of NAD^+ ^binding for their activation [10]. Although NAD and FAD binding proteins are not totally distinct from each other as few proteins bind to both cofactors but through separate domain. Rossmann in 1976 elucidated the βαβαβ as structural motif responsible for the binding of nucleotides specially NAD (P)^+ ^by comparing four known crystal structures of NADBP [11]. At the start of βαβαβ motif, a stretch of 30-35 amino acids was identified that was termed as "fingerprint region" with the consensus phosphate binding sequence (GXGXXG). Later 18 NADBP structures were compared and classified into classical and non-classical structures [12]. NADBP under the classical group follow the same binding orientation as described by Rossmann and a new feature for fingerprint region, a conserved Arg or Lys at the beginning of the first B strand of βαβαβ unit, was identified. However NADBP in non-classical category do not even contain this pattern but still bind to NAD^+^. Domenighini and Rappuoli (1996) also analyzed the NAD^+ ^binding sites in ADP-ribosylating enzymes and extended the NAD^+ ^binding motif slightly but concluded at the end that their proposed model could not account for all NADBP [13]. Shirai *et al *(2006) predicted the individual nucleotide (adenine, guanine, nicotinamide and flavin) binding sites on target proteins but the efficiency was less than 40% [14]. In summary the sequence similarity based methods were unable to characterize all NADBP and annotation was further hindered for the unknown sequences. In the present study, we analyzed 555 NAD-protein complexes obtained from the Protein Data Bank (PDB) to understand the contribution of each type of residue in the NAD^+ ^binding site. The aim of this study is to predict NADBP and NIRs even when there is no similarity with known structures.

NIRs means amino acid residues which interact with the ligand through their side chains. Thus we trained and tested our models on non-redundant set of proteins where no two proteins have sequence identity greater than 40%. SVM based models were developed using amino acid sequence of proteins. In addition we used evolutionary information generated by PSI-BLAST in the form of PSSM profile as an input vector for SVM.

## Results

### Compositional Analysis

**Figure 1:**
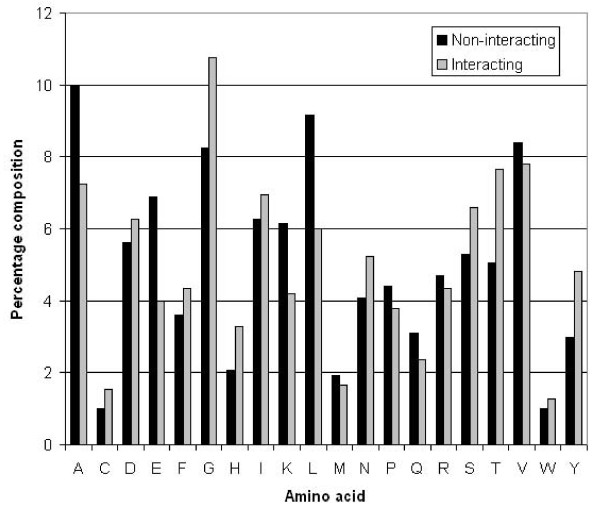
**Percentage composition of NAD interacting and non-interacting residues**.

We calculated the amino acid composition of NIRs and non-NIRs in proteins and observed that certain types of residues like Gly, His, Thr, Ser and Tyr were more abundant in NIRs in comparison to non-NIRs (Figure 1). In the literature it is already known that residues like Gly and His are conserved in the NAD binding sites, which agrees with our analysis. These residues are positively charged, neutral or hydrophobic in nature.

### SVM model using binary pattern

SVM models were generated for window sizes 3 to 21 using amino acid binary patterns as input feature (Table 1 and Additional file 1: Supplemental Table S1-S10). We found maximum accuracy of 74.13% with 0.47 MCC for window size 17. Decreasing or increasing the window size decreases the accuracy as well as specificity. Final model was selected on window size 17, to balance sensitivity and specificity.

**Table 1:** Performance of SVM model developed using amino acid sequence (binary pattern) at different window lengths.

Window size	Kernel parameters	Thr*	Sen (%)	Spe (%)	Acc (%)	MCC
**3**	t 2 g 0.1 j 1 c 1	0	63.41	61.27	62.34	0.25
**5**	t 2 g 0.1 j 1 c 1	0	64.46	65.13	64.79	0.3
**7**	t 2 g 0.1 j 1 c 1	0	67.98	66.83	67.4	0.35
**9**	t 2 g 0.1 j 1 c 1	0	69.09	69.32	69.21	0.38
**11**	t 2 g 0.1 j 1 c 1	0	69.7	71.37	70.54	0.41
**13**	t 2 g 0.1 j 1 c 10	0	70.81	72.78	71.79	0.44
**15**	t 2 g 0.1 j 1 c 10	0	71.56	73.89	72.73	0.45
**17**	**t 1 d 3**	**-0.2**	**70.28**	**76.89**	**74.13**	**0.47**
**19**	t 2 g 0.1 j 1 c 100	0	71.27	72.49	71.88	0.44
**21**	t 2 g 0.1 j 1 c 10	0	70.81	73.68	72.24	0.45

*(Thr- Threshold, Sen - Sensitivity, Spe - Specificity, Acc - Accuracy, MCC - Matthew's correlation coefficient)

SVM models were trained and tested on a dataset having equal number of positive and negative data. Bold font shows the performance and parameters of selected SVM model.

### SVM model using evolutionary information

**Figure 2:**
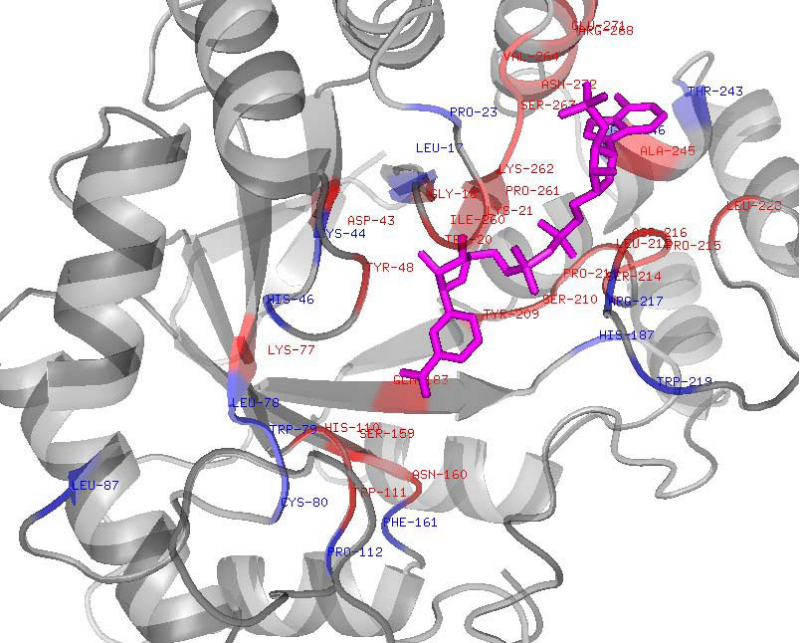
**Structure of human Aldose reductase (**2ACS) **showing prediction of NAD interacting residues by NADbinder**. NAD shown in magenta, True positives in red and False positives in blue colour (only the portion of protein with residue mentioned is shown here).

We have observed in the past [15,16] that evolutionary information provides more information than single sequence. Thus we developed SVM based model using PSSM profiles instead of amino acid sequence, using PSI-BLAST. To optimize window size for predicting NIRs in a protein, we developed SVM models using different window sizes from 3 to 21 (Table 2 and Additional file 1: Supplemental Table S11-S20). We got a maximum accuracy of 87.25% with MCC of 0.75 for 19 amino acid window. Accuracy decreased for window 21 (Additional file 1: Supplemental Table S20). Final model was selected on window size of 19. To show the NADbinder prediction more intuitively, we did prediction on human aldose reductase (UniProt- P15121, PDB- 2ACS) as shown in Figure 2, marking NAD, true positive and false positive residues.

**Table 2:** Performance of SVM models developed using PSSM profile of proteins at different window lengths.

Window size	Kernel parameters	Thr*	Sen (%)	Spe (%)	Acc (%)	MCC
**3**	t 2 g 1.0 j 1 c 10	0	83.26	82.61	82.93	0.66
**5**	t 2 g 1.0 j 1 c 10	0	82.59	87.51	85.05	0.7
**7**	t 2 g 0.1 j 1 c 10	0	82.39	84.67	83.53	0.67
**9**	t 2 g 0.1 j 1 c 10	0	84.18	86.13	85.16	0.7
**11**	t 2 g 0.1 j 1 c 10	0	85.28	86.25	85.77	0.72
**13**	t 2 g 0.1 j 1 c 10	0	85.7	86.52	86.11	0.72
**15**	t 2 g 0.1 j 1 c 10	0	85.36	86.31	85.84	0.72
**17**	t 2 g 0.1 j 1 c 10	0	83.69	87.99	85.84	0.72
**19**	**t 2 g 0.1 j 1 c 10**	**0**	**86.13**	**88.37**	**87.25**	**0.75**
**21**	t 2 g 0.1 j 1 c 10	0	85.52	87.33	86.43	0.73

SVM models were trained and tested on a dataset having equal number of positive and negative data. Bold font shows the performance and parameters of selected SVM model.

### ROC Plot

**Figure 3:**
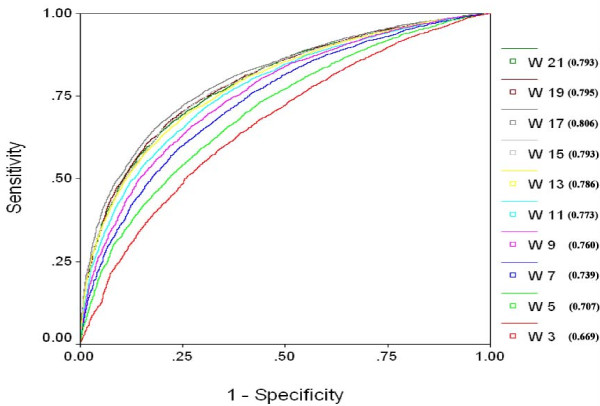
**ROC Plot for SVM models developed using single sequence (binary pattern) for window size from 3 to 21**. (W indicates the window length and value in bracket shows Area under curve).

**Figure 4:**
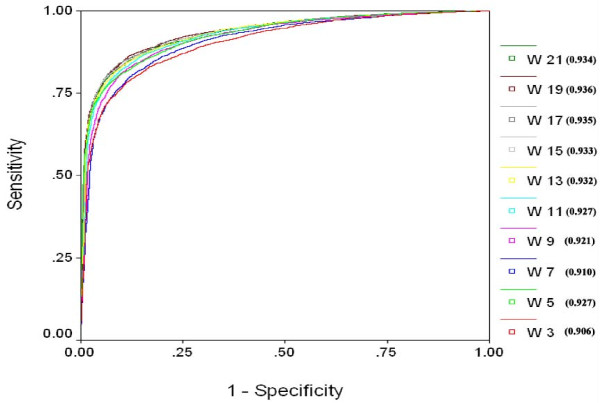
**ROC Plot for PSSM based SVM models developed using window size from 3 to 21**. (W indicates the window length and value in bracket shows Area under curve).

In order to have a threshold independent evaluation of our method, we created ROC (receiver operating curve) for all the models. ROC plots with Area under curve (AUC) values were created by using SPSS statistical package. ROC plots using binary feature for different window lengths (Figure 3) clearly show that window size 17 is best suited for sequence based prediction and window size 19 is most suitable for SVM based model using PSSM (Figure 4).

### Analysis of the rate of false positive prediction

Like any other prediction server, NADbinder could also give some false positive prediction. In order to analyse the rate of false positive prediction by NADbinder, we evaluate our method on a dataset of NAD binding and non-NAD binding proteins. This dataset contain original data of NAD binding proteins and 137 non-NAD binding (negative) proteins (non-redundant at 40% CDHIT) which do not bind to any ligands extracted from the Protein Data Bank (PDB). Combined positive and negative data was subjected to 5 fold cross validation. 4 sets were trained on the optimized parameter of the SVM and 5th set was tested. We tested the positive proteins for the sensitivity and result of negative proteins gave the specificity and ultimately accuracy of the prediction. The question arises whether we can discriminate NAD and non-NAD binding proteins based on the percent of NIRs prediction. For each protein we calculate the percentage of predicted NIRs over length i.e. **(true positive+false positive)/length **at threshold 0, 0.1, 0.2 and 0.3. At the threshold of 0.3, we find a balance between sensitivity and specificity where accuracy is achievable up to 72% if used 10% prediction cutoff (see Additional file 1: Table S28). In short if user submits an unknown protein of 100 residues and 10 or more residues are predicted to be NIRs by the server at threshold 0.3 then the accuracy of prediction will be 72% otherwise the prediction could be considered as false positive.

### Description of Web server

A user-friendly web server 'NADbinder' was developed for the prediction of NIRs in uncharacterized proteins. The user may submit the amino acid sequence(s) in 'FASTA' format. The server generates the evolutionary profile of all submitted sequences and predicts NIRs. As this sever allows users to select threshold, we suggest the users to select higher value if they are interested in high specificity (high confidence). In case the user is more interested to cover most of NAD interacting residues (high sensitivity) then they should select lower threshold. In the output NAD interacting residues are displayed underlined in red. The web-server is freely available at http://www.imtech.res.in/raghava/nadbinder.

## Discussion

Small molecular weight cofactors play very important role in the proper functioning of the cellular machinery. Enzyme and ligand research over the past many years led to very useful databases and tools for the proper annotation of ligand targets and enzymes. NAD^+ ^is one of the factors which bind to proteins having role in energy transfer, storage, or signal transduction. Binding of NAD^+ ^changes the conformation of target proteins and accordingly controls their function. For the understanding of their mechanism, structure determination is a prerequisite, which is a very time consuming process. Researchers have been trying to analyze the NAD binding motif and could slightly improve the original nucleotide binding motif identified by Rossmann. Classical NAD^+ ^binding proteins follow the Rossmann fold model but non-classical proteins bind to the NAD^+ ^even without Rossmann fold. So far no model could fit all classical and non-classical NAD^+ ^binding proteins. Analysis of these proteins through similarity based approaches could not derive an efficient rule for NAD^+ ^binding site. Due to the complexity in structure determination and limitations of available tools there is a need to develop a sequence and non-alignment based computational methods for the identification of NAD^+ ^binding sites and their interacting amino acid residues in proteins. Therefore we developed the SVM based method using amino acid binary pattern and PSSM derived evolutionary profile. PSSM profile model performed far better than amino acid binary pattern. We validated the performance of our PSSM model on the classical/non-classical NAD binding proteins and found that it is able to predict NIRs in those proteins that were not included in the training process. Like in classical proteins malate dehydrogenase (PDB: 1CME; UniProt: P61889) and in non-classical proteins aldose reductase (2ACS, P15121), isocitrate dehydrogenase (9ICD, P08200) etc were successfully analyzed for their NIRs (only 2ACS result shown). However we accept the fact that there might be few false positive interactions also like in any other prediction method. False positive prediction analysis of the server has been done by taking negative proteins form the PDB and found that prediction threshold of 0.3 is the optimum. Based on the above algorithm we developed 'NADbinder' web-server, which is freely available in the hope that it will help biologists in the identification of NAD binding proteins and their interacting residues for the purpose of annotation, structural elucidation and structure-function analysis.

## Conclusion

In order to understand the proper mechanism of action and annotation of NAD^+ ^binding proteins we need to know the amino acid residues interacting with NAD^+^. Homology and sequence similarity based methods have been proved limited for the uncharacterized proteins. Therefore we developed a SVM based method which, require only protein sequence for the prediction of NAD^+ ^binding proteins and their interacting residues without prior knowledge of structural information.

## Methods

### Dataset

We extracted 555 PDB ids from SuperSite database [17] that bind to NAD by giving 'NAD' in the cofactor search field. We used these PDBs in the Ligand Protein Contact (LPC) server [18] and obtained 1556 amino acid chains with contact details. In the present study we considered residues with direct side chain contact with NAD and marked with an asterisk '*' in the LPC output to define the NIR. Using CD-HIT [19] only the non-redundant protein chains, where no two chains had sequence identity greater than 40%, were included in the main dataset yielding 195 NAD interacting protein chains. Although we reduced the redundancy at 90 and 60% also (data not shown), 40% was chosen balance between number of sequences and redundancy. In the past 40% cut off was also used by others [16]. From 195 NAD interacting chains we extracted 4772 NIRs and the rest of the amino acid residues from each protein were considered as non-NIRs (61256). Equal (4772 positives and 4772 negatives) and real (4772 positives and 61256 negatives) datasets were created. All dataset is provided in additional file 2.

### Cross Validation

Five-fold cross-validation technique was used to evaluate the performance of all the models. Here sequences are randomly divided into five sets of which four sets are used for training and the remaining fifth set for testing. The process is repeated five times in such a way that each set is used once for testing. Final performance is obtained by averaging the performance of all the five sets.

### Pattern or Window Size

It is well established fact that the structural state (i.e. secondary structure) of a residue is not determined only by amino acid residue itself but also affected by neighboring residues. To have the information from the neighboring residues, for each sequence we created overlapping patterns of different windows size from 3 to 21 amino acid length. Considering NIR at central residue, we classified the pattern as positive or NAD interacting pattern and otherwise termed as negative or non-interacting pattern. This is similar to the approach adopted by Kumar and Raghava for the prediction of RNA binding sites in proteins [15]. To create a pattern for the terminal residues we added (L-1)/2 number of dummy residue 'X' at both termini of the protein sequence (L is length of the protein sequence). It means for window size 17 we added 8 'X' at both sides of the sequence.

### Input Features

#### Binary profile

Different window patterns were converted into binary profile [20,21]. Each amino acid in a pattern was represented by a vector of dimension 21 (e.g. Ala by 1,0,0,0,0,0,0,0,0,0,0,0,0,0,0,0,0,0,0,0,0), which contained 20 amino acids and one dummy amino acid 'X'. A pattern of window length W was represented by a vector of dimension 21 × W.

#### Evolutionary profile

Second input feature used was evolutionary profile in which NAD binding sequences were used for PSSM profile generation [15,22]. This was incorporated by using position-specific scoring matrix (PSSM) generated during PSI-BLAST [23] search against non-redundant (nr) database. The PSSM matrix was generated by three iterations of searching with cutoff e-value of 0.001. PSSM matrix represents the probability of occurrence of each amino acid residue at each position i.e. residue conservation at a particular position in a sequence. Here we created the vector of 20 × W dimensions.

#### Support Vector Machine (SVM)

In the present study, SVM classifier was used from freely available SVM_light package [24]. This package is powerful as well as user-friendly where we can adjust the parameters and kernel functions like Linear, Polynomial, RBF and Sigmoid. SVM details can be obtained from Vapnik 1995 [25]. SVM technique has been used successfully in the past for the wide range of bioinformatics applications [26,27].

#### Performance Measures

The performance of various models developed in this study was computed by using threshold- dependent as well as threshold-independent parameters. In threshold-dependent parameters we used sensitivity (Sn), Specificity (Sp) or percent coverage of non-interacting residues, overall accuracy (Acc) and Matthew's correlation coefficient (MCC) using following equations. For threshold-independent parameter ROC plots were generated for all models.

[TP- true positive; FN- false negative; TN- true negative; FP- false positive]

## Authors' contributions

HRA carried out the data analysis and interpretation, developed computer programs, wrote the manuscript and developed the web-server. GPSR conceived and coordinated the project, guided its conception and design, helped in the interpretation of data, refined the drafted manuscript and gave overall supervision to the project. Both authors read and approved the final manuscript.

## Supplementary Material

Additional file 1 **Model performance at different window lengths**. File containing model performance for binary and PSSM profiles at different window lengths as well as comparison with BLAST. Click here for file

Additional file 2 **Data used in the development of server**. File contains data including protein sequences with NAD interacting residues and PDB IDs with and without redundancy. Click here for file
