# Supplementary material for: Identification of NAD interacting residues in proteins
Source: BMC Bioinformatics. 2010 Mar 30;11:160. doi: 10.1186/1471-2105-11-160 (PMC2853471; doi:10.1186/1471-2105-11-160)
Supplement: Additional file 1 — Model performance at different window lengths. File containing model performance for binary and PSSM profiles at different window lengths as well as comparison with BLAST. [file 1471-2105-11-160-S1.DOC]

**SUPPLEMENTARY MATERIAL-**

**Additional File 1**

# Identification of NAD interacting residues in proteins

# Hifzur R Ansari, Gajendra PS Raghava^§^

Institute of Microbial Technology, Sector- 39A, Chandigarh, India-160036.

^§^Corresponding author

Email: HRA - hrahman@imtech.res.in; GPSR - raghava@imtech.res.in

**1]** Performance of SVM model developed using **amino acid sequence (binary pattern)** at different window lengths. SVM models were trained and tested on a dataset having **equal number of positive and negative data**. Bold row shows the performance of best SVM model.

**Table S1.** **Window size 3** (Kernel Parameters, t 2 g 0.1 j 1 c 1).

| **Threshold** | **Sensitivity (%)** | **Specificity (%)** | **Accuracy (%)** | **MCC** |
| --- | --- | --- | --- | --- |
| **-1.0** | 94.32 | 15.88 | 55.1 | 0.16 |
| **-0.9** | 92.25 | 20.6 | 56.42 | 0.18 |
| **-0.8** | 89.86 | 24.85 | 57.36 | 0.19 |
| **-0.7** | 87.36 | 29.02 | 58.19 | 0.2 |
| **-0.6** | 84.7 | 33.36 | 59.03 | 0.21 |
| **-0.5** | 81.71 | 38.18 | 59.94 | 0.22 |
| **-0.4** | 78.25 | 42.77 | 60.51 | 0.22 |
| **-0.3** | 74.56 | 47.61 | 61.09 | 0.23 |
| **-0.2** | 70.83 | 52.12 | 61.47 | 0.23 |
| **-0.1** | 67.2 | 56.94 | 62.07 | 0.24 |
| **0** | **63.41** | **61.27** | **62.34** | **0.25** |
| **0.1** | 59.41 | 65.49 | 62.45 | 0.25 |
| **0.2** | 55.49 | 69.49 | 62.49 | 0.25 |
| **0.3** | 50.78 | 73.76 | 62.27 | 0.25 |
| **0.4** | 45.6 | 77.12 | 61.36 | 0.24 |
| **0.5** | 41.47 | 80.32 | 60.9 | 0.24 |
| **0.6** | 37.3 | 83.61 | 60.46 | 0.24 |
| **0.7** | 32.52 | 86.5 | 59.51 | 0.23 |
| **0.8** | 27.93 | 89 | 58.47 | 0.21 |
| **0.9** | 23.97 | 91.09 | 57.53 | 0.2 |
| **1.0** | 16.01 | 94.17 | 55.09 | 0.16 |

**Table S2.** **Window size 5** (Kernel Parameters, t 2 g 0.1 j 1 c 1).

| **Threshold** | **Sensitivity (%)** | **Specificity (%)** | **Accuracy (%)** | **MCC** |
| --- | --- | --- | --- | --- |
| **-1.0** | 94.95 | 17.69 | 56.32 | 0.2 |
| **-0.9** | 93.52 | 21.77 | 57.65 | 0.22 |
| **-0.8** | 91.66 | 25.65 | 58.65 | 0.23 |
| **-0.7** | 89.4 | 30.05 | 59.72 | 0.24 |
| **-0.6** | 86.82 | 35.21 | 61.01 | 0.26 |
| **-0.5** | 83.8 | 39.9 | 61.85 | 0.26 |
| **-0.4** | 80.72 | 45.18 | 62.95 | 0.28 |
| **-0.3** | 77.1 | 50.34 | 63.72 | 0.28 |
| **-0.2** | 73.45 | 55.62 | 64.53 | 0.3 |
| **-0.1** | 69.11 | 60.44 | 64.77 | 0.3 |
| **0** | **64.46** | **65.13** | **64.79** | **0.3** |
| **0.1** | 60 | 69.51 | 64.75 | 0.3 |
| **0.2** | 55.47 | 73.85 | 64.66 | 0.3 |
| **0.3** | 51.03 | 77.87 | 64.45 | 0.3 |
| **0.4** | 46.02 | 81.68 | 63.85 | 0.3 |
| **0.5** | 41.49 | 84.97 | 63.23 | 0.29 |
| **0.6** | 36.21 | 87.99 | 62.1 | 0.28 |
| **0.7** | 31.71 | 90.76 | 61.23 | 0.28 |
| **0.8** | 27.01 | 92.73 | 59.87 | 0.26 |
| **0.9** | 22.82 | 94.49 | 58.65 | 0.25 |
| **1.0** | 18.48 | 95.87 | 57.18 | 0.23 |

**Table S3.** **Window size 7** (Kernel Parameters, t 2 g 0.1 j 1 c 1).

| **Threshold** | **Sensitivity (%)** | **Specificity (%)** | **Accuracy (%)** | **MCC** |
| --- | --- | --- | --- | --- |
| **-1.0** | 95.39 | 19.17 | 57.28 | 0.22 |
| **-0.9** | 93.92 | 22.84 | 58.38 | 0.24 |
| **-0.8** | 92.62 | 26.57 | 59.6 | 0.26 |
| **-0.7** | 91.09 | 31.45 | 61.27 | 0.28 |
| **-0.6** | 88.66 | 36.88 | 62.77 | 0.3 |
| **-0.5** | 86.36 | 41.79 | 64.07 | 0.31 |
| **-0.4** | 83.36 | 47.25 | 65.31 | 0.33 |
| **-0.3** | 80.09 | 52.12 | 66.1 | 0.34 |
| **-0.2** | 76.36 | 57.52 | 66.94 | 0.35 |
| **-0.1** | 72.11 | 62.15 | 67.13 | 0.34 |
| **0** | **67.98** | **66.83** | **67.4** | **0.35** |
| **0.1** | 63.41 | 71.65 | 67.53 | 0.35 |
| **0.2** | 59.01 | 76.24 | 67.62 | 0.36 |
| **0.3** | 53.77 | 79.92 | 66.85 | 0.35 |
| **0.4** | 48.68 | 83.65 | 66.17 | 0.35 |
| **0.5** | 43.57 | 86.74 | 65.15 | 0.34 |
| **0.6** | 37.76 | 89.15 | 63.45 | 0.31 |
| **0.7** | 33.07 | 91.58 | 62.32 | 0.3 |
| **0.8** | 28.56 | 93.61 | 61.09 | 0.29 |
| **0.9** | 23.16 | 95.31 | 59.23 | 0.27 |
| **1.0** | 18.63 | 96.84 | 57.73 | 0.25 |

**Table S4.** **Window size 9** (Kernel Parameters, t 2 g 0.1 j 1 c 1).

| **Threshold** | **Sensitivity (%)** | **Specificity (%)** | **Accuracy (%)** | **MCC** |
| --- | --- | --- | --- | --- |
| **-1.0** | 96.52 | 18.02 | 57.27 | 0.23 |
| **-0.9** | 95.33 | 22.15 | 58.74 | 0.26 |
| **-0.8** | 93.9 | 26.55 | 60.23 | 0.28 |
| **-0.7** | 92.25 | 31.43 | 61.84 | 0.3 |
| **-0.6** | 89.77 | 37.09 | 63.43 | 0.32 |
| **-0.5** | 87.53 | 42.08 | 64.81 | 0.33 |
| **-0.4** | 85.18 | 47.99 | 66.59 | 0.36 |
| **-0.3** | 81.71 | 53.46 | 67.58 | 0.37 |
| **-0.2** | 77.43 | 59.03 | 68.23 | 0.37 |
| **-0.1** | 73.72 | 64.19 | 68.95 | 0.38 |
| **0** | **69.09** | **69.32** | **69.21** | **0.38** |
| **0.1** | 64.48 | 74.1 | 69.29 | 0.39 |
| **0.2** | 59.62 | 78.5 | 69.06 | 0.39 |
| **0.3** | 53.67 | 82.88 | 68.27 | 0.38 |
| **0.4** | 48.62 | 86.34 | 67.48 | 0.38 |
| **0.5** | 43.32 | 88.94 | 66.13 | 0.36 |
| **0.6** | 37.3 | 91.14 | 64.22 | 0.34 |
| **0.7** | 32.4 | 93.27 | 62.84 | 0.32 |
| **0.8** | 27.51 | 94.93 | 61.22 | 0.3 |
| **0.9** | 22.82 | 96.35 | 59.59 | 0.28 |
| **1.0** | 18.84 | 97.42 | 58.13 | 0.26 |

**Table S5.** **Window size 11** (Kernel Parameters, t 2 g 0.1 j 1 c 1).

| **Threshold** | **Sensitivity (%)** | **Specificity (%)** | **Accuracy (%)** | **MCC** |
| --- | --- | --- | --- | --- |
| **-1.0** | 97.07 | 15.38 | 56.22 | 0.22 |
| **-0.9** | 95.91 | 19.47 | 57.69 | 0.24 |
| **-0.8** | 94.4 | 24.54 | 59.47 | 0.26 |
| **-0.7** | 92.83 | 29.02 | 60.93 | 0.28 |
| **-0.6** | 90.84 | 35.56 | 63.2 | 0.32 |
| **-0.5** | 88.62 | 41.58 | 65.1 | 0.34 |
| **-0.4** | 85.6 | 47.76 | 66.68 | 0.36 |
| **-0.3** | 82.19 | 54.25 | 68.22 | 0.38 |
| **-0.2** | 78.63 | 60.39 | 69.51 | 0.4 |
| **-0.1** | 74.5 | 65.91 | 70.2 | 0.41 |
| **0** | **69.7** | **71.37** | **70.54** | **0.41** |
| **0.1** | 63.96 | 76.53 | 70.24 | 0.41 |
| **0.2** | 58.32 | 81.31 | 69.81 | 0.41 |
| **0.3** | 52.62 | 85.44 | 69.03 | 0.4 |
| **0.4** | 47.09 | 88.77 | 67.93 | 0.39 |
| **0.5** | 41.7 | 91.24 | 66.47 | 0.38 |
| **0.6** | 35.9 | 93.21 | 64.55 | 0.36 |
| **0.7** | 30.83 | 94.93 | 62.88 | 0.34 |
| **0.8** | 25.78 | 96.48 | 61.13 | 0.31 |
| **0.9** | 21.35 | 97.65 | 59.5 | 0.29 |
| **1.0** | 15.99 | 98.28 | 57.14 | 0.25 |

**Table S6.** **Window size 13** (Kernel Parameters, t 2 g 0.1 j 1 c 10).

| **Threshold** | **Sensitivity (%)** | **Specificity (%)** | **Accuracy (%)** | **MCC** |
| --- | --- | --- | --- | --- |
| **-1.0** | 99.35 | 4.67 | 52.01 | 0.12 |
| **-0.9** | 98.89 | 7.8 | 53.34 | 0.16 |
| **-0.8** | 98.26 | 11.38 | 54.82 | 0.19 |
| **-0.7** | 97.25 | 16.01 | 56.63 | 0.23 |
| **-0.6** | 95.54 | 22.07 | 58.8 | 0.26 |
| **-0.5** | 93.38 | 29.51 | 61.44 | 0.3 |
| **-0.4** | 90.57 | 38.14 | 64.35 | 0.34 |
| **-0.3** | 86.78 | 46.88 | 66.83 | 0.37 |
| **-0.2** | 82.12 | 56.2 | 69.16 | 0.4 |
| **-0.1** | 76.82 | 64.59 | 70.7 | 0.42 |
| **0** | **70.81** | **72.78** | **71.79** | **0.44** |
| **0.1** | 63.37 | 79.67 | 71.52 | 0.44 |
| **0.2** | 56.04 | 85.33 | 70.68 | 0.43 |
| **0.3** | 48.24 | 89.75 | 69 | 0.42 |
| **0.4** | 40.78 | 92.79 | 66.79 | 0.39 |
| **0.5** | 33.59 | 95.31 | 64.45 | 0.37 |
| **0.6** | 26.99 | 97.02 | 62.01 | 0.34 |
| **0.7** | 21.02 | 98.09 | 59.56 | 0.3 |
| **0.8** | 15.3 | 98.93 | 57.11 | 0.26 |
| **0.9** | 10.46 | 99.25 | 54.85 | 0.21 |
| **1.0** | 7.31 | 99.6 | 53.46 | 0.18 |

**Table S7.** **Window size 15** (Kernel Parameters, t 2 g 0.1 j 1 c 10).

| **Threshold** | **Sensitivity (%)** | **Specificity (%)** | **Accuracy (%)** | **MCC** |
| --- | --- | --- | --- | --- |
| **-1.0** | 99.6 | 3.44 | 51.52 | 0.11 |
| **-0.9** | 99.35 | 5.66 | 52.5 | 0.14 |
| **-0.8** | 98.81 | 9.24 | 54.02 | 0.18 |
| **-0.7** | 97.86 | 13.64 | 55.75 | 0.21 |
| **-0.6** | 96.42 | 19.45 | 57.93 | 0.25 |
| **-0.5** | 94.11 | 27.64 | 60.88 | 0.29 |
| **-0.4** | 91.35 | 36.19 | 63.77 | 0.33 |
| **-0.3** | 87.64 | 46.14 | 66.89 | 0.37 |
| **-0.2** | 83.57 | 55.99 | 69.78 | 0.41 |
| **-0.1** | 78.48 | 65.47 | 71.97 | 0.44 |
| **0** | **71.56** | **73.89** | **72.73** | **0.45** |
| **0.1** | 63.37 | 80.51 | 71.94 | 0.45 |
| **0.2** | 54.67 | 86.11 | 70.39 | 0.43 |
| **0.3** | 47.3 | 90.61 | 68.95 | 0.42 |
| **0.4** | 39.02 | 93.84 | 66.43 | 0.39 |
| **0.5** | 31.56 | 96.25 | 63.9 | 0.36 |
| **0.6** | 23.72 | 97.61 | 60.67 | 0.32 |
| **0.7** | 17.5 | 98.7 | 58.1 | 0.28 |
| **0.8** | 12.3 | 99.2 | 55.75 | 0.23 |
| **0.9** | 8.53 | 99.66 | 54.1 | 0.2 |
| **1.0** | 5.34 | 99.81 | 52.58 | 0.16 |

**Table S8:** **Window size 17** (Kernel Parameters, t 1 d 3).

| **Threshold** | **Sensitivity (%)** | **Specificity (%)** | **Accuracy (%)** | **MCC** |
| --- | --- | --- | --- | --- |
| -1.0 | 99.46 | 3.98 | 43.9 | 0.11 |
| -0.9 | 99.08 | 7.71 | 45.91 | 0.16 |
| -0.8 | 97.84 | 13.04 | 48.5 | 0.19 |
| -0.7 | 95.87 | 21.91 | 52.83 | 0.25 |
| -0.6 | 93.21 | 31.89 | 57.53 | 0.3 |
| -0.5 | 89.08 | 44 | 62.85 | 0.36 |
| -0.4 | 83.57 | 56.65 | 67.91 | 0.41 |
| -0.3 | 77.47 | 68.05 | 71.99 | 0.45 |
| **-0.2** | **70.28** | **76.89** | **74.13** | **0.47** |
| -0.1 | 61.36 | 83.87 | 74.46 | 0.47 |
| 0 | 53.04 | 89.26 | 74.12 | 0.46 |
| 0.1 | 45.35 | 93.34 | 73.28 | 0.45 |
| 0.2 | 38.26 | 95.59 | 71.62 | 0.43 |
| 0.3 | 30.68 | 97.3 | 69.45 | 0.39 |
| 0.4 | 24.46 | 98.45 | 67.51 | 0.36 |
| 0.5 | 19.11 | 99.23 | 65.73 | 0.33 |
| 0.6 | 14.67 | 99.67 | 64.13 | 0.29 |
| 0.7 | 10.83 | 99.79 | 62.6 | 0.25 |
| 0.8 | 7.59 | 99.88 | 61.29 | 0.21 |
| 0.9 | 5.41 | 99.94 | 60.41 | 0.18 |
| 1.0 | 3.5 | 99.95 | 59.62 | 0.14 |

**Table S9.** **Window size 19** (Kernel Parameters, t 2 g 0.1 j 1 c 100).

| **Threshold** | **Sensitivity (%)** | **Specificity (%)** | **Accuracy (%)** | **MCC** |
| --- | --- | --- | --- | --- |
| **-1.0** | 99.87 | 1.36 | 50.62 | 0.07 |
| **-0.9** | 99.77 | 3.02 | 51.39 | 0.11 |
| **-0.8** | 99.37 | 5.57 | 52.47 | 0.14 |
| **-0.7** | 98.68 | 9.14 | 53.91 | 0.18 |
| **-0.6** | 97.78 | 14.61 | 56.19 | 0.22 |
| **-0.5** | 96.29 | 22.61 | 59.45 | 0.28 |
| **-0.4** | 93.8 | 31.75 | 62.77 | 0.33 |
| **-0.3** | 90.32 | 41.76 | 66.04 | 0.37 |
| **-0.2** | 85.46 | 53.08 | 69.27 | 0.41 |
| **-0.1** | 79.09 | 63.12 | 71.1 | 0.43 |
| **0** | **71.27** | **72.49** | **71.88** | **0.44** |
| **0.1** | 62.24 | 81.39 | 71.81 | 0.44 |
| **0.2** | 52.79 | 87.87 | 70.33 | 0.43 |
| **0.3** | 43.61 | 92.9 | 68.25 | 0.42 |
| **0.4** | 33.57 | 95.7 | 64.64 | 0.37 |
| **0.5** | 24.6 | 97.63 | 61.12 | 0.33 |
| **0.6** | 17.35 | 98.64 | 57.99 | 0.27 |
| **0.7** | 11.48 | 99.16 | 55.32 | 0.22 |
| **0.8** | 6.79 | 99.58 | 53.19 | 0.17 |
| **0.9** | 3.98 | 99.71 | 51.84 | 0.13 |
| **1.0** | 2.35 | 99.81 | 51.08 | 0.1 |

**Table S10.** **Window size 21** (Kernel Parameters, t 2 g 0.1 j 1 c 10).

| **Threshold** | **Sensitivity (%)** | **Specificity (%)** | **Accuracy (%)** | **MCC** |
| --- | --- | --- | --- | --- |
| **-1.0** | 99.94 | 0.63 | 50.28 | 0.05 |
| **-0.9** | 99.85 | 1.63 | 50.74 | 0.08 |
| **-0.8** | 99.64 | 3.29 | 51.47 | 0.11 |
| **-0.7** | 99.27 | 6.64 | 52.95 | 0.16 |
| **-0.6** | 98.28 | 10.94 | 54.61 | 0.19 |
| **-0.5** | 96.86 | 18.59 | 57.72 | 0.25 |
| **-0.4** | 94.49 | 27.58 | 61.03 | 0.3 |
| **-0.3** | 91.01 | 38.54 | 64.77 | 0.35 |
| **-0.2** | 85.83 | 50.82 | 68.33 | 0.39 |
| **-0.1** | 78.81 | 62.59 | 70.7 | 0.42 |
| **0** | **70.81** | **73.68** | **72.24** | **0.45** |
| **0.1** | 60.48 | 82.67 | 71.57 | 0.44 |
| **0.2** | 50.52 | 89.33 | 69.93 | 0.43 |
| **0.3** | 39.98 | 93.57 | 66.77 | 0.4 |
| **0.4** | 30.51 | 96.08 | 63.3 | 0.35 |
| **0.5** | 22.65 | 97.97 | 60.31 | 0.31 |
| **0.6** | 15.53 | 99.02 | 57.27 | 0.26 |
| **0.7** | 10.02 | 99.56 | 54.79 | 0.22 |
| **0.8** | 6.06 | 99.81 | 52.93 | 0.17 |
| **0.9** | 3.56 | 99.94 | 51.75 | 0.13 |
| **1.0** | 1.89 | 99.98 | 50.93 | 0.1 |

**2]** Performance of SVM model developed using **evolutionary information in the form of PSSM profile generated by PSI-BLAST** for different window lengths. SVM models were trained and tested on a dataset having **equal number of positive and negative data**. Bold row shows the performance of best SVM model.

**Table S11.** **Window size 3** (Kernel Parameters, t 2 g 1.0 j 1 c 10).

| **Threshold** | **Sensitivity (%)** | **Specificity (%)** | **Accuracy (%)** | **MCC** |
| --- | --- | --- | --- | --- |
| **-1.0** | 97.99 | 30.3 | 64.15 | 0.38 |
| **-0.9** | 97.27 | 37.2 | 67.23 | 0.43 |
| **-0.8** | 96.14 | 43.42 | 69.78 | 0.47 |
| **-0.7** | 94.92 | 49.12 | 72.02 | 0.5 |
| **-0.6** | 93.96 | 54.89 | 74.42 | 0.53 |
| **-0.5** | 92.25 | 60.4 | 76.32 | 0.56 |
| **-0.4** | 90.85 | 64.98 | 77.92 | 0.58 |
| **-0.3** | 89.38 | 69.94 | 79.66 | 0.6 |
| **-0.2** | 87.23 | 74.35 | 80.79 | 0.62 |
| **-0.1** | 85.34 | 78.54 | 81.94 | 0.64 |
| **0** | 83.26 | 82.61 | 82.93 | 0.66 |
| **0.1** | 80.86 | 85.46 | 83.16 | 0.66 |
| **0.2** | 78.73 | 88.2 | 83.46 | 0.67 |
| **0.3** | 76.06 | 90.4 | 83.23 | 0.67 |
| **0.4** | 73.42 | 92.7 | 83.06 | 0.67 |
| **0.5** | 70.35 | 94.37 | 82.36 | 0.67 |
| **0.6** | 66.81 | 95.69 | 81.25 | 0.65 |
| **0.7** | 62.8 | 96.46 | 79.63 | 0.63 |
| **0.8** | 58.39 | 97.36 | 77.88 | 0.61 |
| **0.9** | 53.69 | 98.11 | 75.9 | 0.58 |
| **1.0** | 39.27 | 98.74 | 69 | 0.47 |

**Table S12.** **Window size 5** (Kernel Parameters, t 2 g 1.0 j 1 c 10).

| **Threshold** | **Sensitivity (%)** | **Specificity (%)** | **Accuracy (%)** | **MCC** |
| --- | --- | --- | --- | --- |
| **-1.0** | 99.53 | 14.93 | 57.23 | 0.27 |
| **-0.9** | 99.11 | 23.79 | 61.45 | 0.35 |
| **-0.8** | 98.49 | 31.36 | 64.92 | 0.4 |
| **-0.7** | 97.84 | 39.62 | 68.73 | 0.46 |
| **-0.6** | 96.68 | 48.14 | 72.41 | 0.51 |
| **-0.5** | 95.14 | 56.92 | 76.03 | 0.56 |
| **-0.4** | 93.15 | 64.53 | 78.84 | 0.6 |
| **-0.3** | 91.52 | 71.49 | 81.51 | 0.64 |
| **-0.2** | 88.55 | 77.91 | 83.23 | 0.67 |
| **-0.1** | 85.87 | 83.1 | 84.49 | 0.69 |
| **0** | **82.59** | **87.51** | **85.05** | **0.7** |
| **0.1** | 79.76 | 91.25 | 85.5 | 0.71 |
| **0.2** | 76.53 | 94.02 | 85.27 | 0.72 |
| **0.3** | 73.8 | 95.55 | 84.67 | 0.71 |
| **0.4** | 70.78 | 97.01 | 83.9 | 0.7 |
| **0.5** | 67.32 | 97.76 | 82.54 | 0.68 |
| **0.6** | 63.74 | 98.17 | 80.96 | 0.66 |
| **0.7** | 59.45 | 98.7 | 79.08 | 0.63 |
| **0.8** | 54.5 | 99.1 | 76.8 | 0.6 |
| **0.9** | 47.39 | 99.47 | 73.43 | 0.55 |
| **1.0** | 31.36 | 99.72 | 65.54 | 0.43 |

**Table S13.** **Window size 7** (Kernel Parameters, t 2 g 0.1 j 1 c 10).

| **Threshold** | **Sensitivity (%)** | **Specificity (%)** | **Accuracy (%)** | **MCC** |
| --- | --- | --- | --- | --- |
| **-1.0** | 96.42 | 44.21 | 70.31 | 0.48 |
| **-0.9** | 95.73 | 49.89 | 72.81 | 0.51 |
| **-0.8** | 94.65 | 55.07 | 74.86 | 0.54 |
| **-0.7** | 93.49 | 60.24 | 76.86 | 0.57 |
| **-0.6** | 92.54 | 65.16 | 78.85 | 0.6 |
| **-0.5** | 91.19 | 69.33 | 80.26 | 0.62 |
| **-0.4** | 89.61 | 72.87 | 81.24 | 0.63 |
| **-0.3** | 87.94 | 76.45 | 82.2 | 0.65 |
| **-0.2** | 86.37 | 79.5 | 82.93 | 0.66 |
| **-0.1** | 84.4 | 82.41 | 83.41 | 0.67 |
| **0** | **82.39** | **84.67** | **83.53** | **0.67** |
| **0.1** | 80.7 | 87.19 | 83.95 | 0.68 |
| **0.2** | 78.26 | 89.24 | 83.75 | 0.68 |
| **0.3** | 76.08 | 91.13 | 83.6 | 0.68 |
| **0.4** | 73.76 | 92.58 | 83.17 | 0.68 |
| **0.5** | 71.39 | 93.86 | 82.63 | 0.67 |
| **0.6** | 68.86 | 94.81 | 81.83 | 0.66 |
| **0.7** | 65.75 | 95.83 | 80.79 | 0.65 |
| **0.8** | 61.81 | 96.68 | 79.24 | 0.62 |
| **0.9** | 57.8 | 97.21 | 77.5 | 0.6 |
| **1.0** | 48.67 | 97.82 | 73.24 | 0.53 |

**Table S14.** **Window size 9** (Kernel Parameters, t 2 g 0.1 j 1 c 10).

| **Threshold** | **Sensitivity (%)** | **Specificity (%)** | **Accuracy (%)** | **MCC** |
| --- | --- | --- | --- | --- |
| **-1.0** | 97.32 | 41.53 | 69.43 | 0.47 |
| **-0.9** | 96.42 | 48.51 | 72.47 | 0.51 |
| **-0.8** | 95.53 | 54.5 | 75.01 | 0.55 |
| **-0.7** | 94.26 | 59.93 | 77.09 | 0.58 |
| **-0.6** | 93.21 | 65.14 | 79.18 | 0.61 |
| **-0.5** | 92.19 | 69.47 | 80.83 | 0.63 |
| **-0.4** | 90.6 | 73.85 | 82.23 | 0.65 |
| **-0.3** | 89.02 | 77.34 | 83.18 | 0.67 |
| **-0.2** | 87.68 | 80.72 | 84.2 | 0.69 |
| **-0.1** | 86.01 | 83.47 | 84.74 | 0.7 |
| **0** | **84.18** | **86.13** | **85.16** | **0.7** |
| **0.1** | 82.65 | 88.37 | 85.51 | 0.71 |
| **0.2** | 80.72 | 90.26 | 85.49 | 0.71 |
| **0.3** | 78.65 | 91.66 | 85.16 | 0.71 |
| **0.4** | 76.31 | 93.11 | 84.71 | 0.7 |
| **0.5** | 73.7 | 94.16 | 83.93 | 0.69 |
| **0.6** | 71.02 | 95.38 | 83.2 | 0.68 |
| **0.7** | 67.74 | 96.26 | 82 | 0.67 |
| **0.8** | 64.27 | 97.03 | 80.65 | 0.65 |
| **0.9** | 59.93 | 97.74 | 78.83 | 0.62 |
| **1.0** | 49.22 | 98.23 | 73.73 | 0.54 |

**Table S15.** **Window size 11** (Kernel Parameters, t 2 g 0.1 j 1 c 10).

| **Threshold** | **Sensitivity (%)** | **Specificity (%)** | **Accuracy (%)** | **MCC** |
| --- | --- | --- | --- | --- |
| **-1.0** | 97.66 | 37.99 | 67.82 | 0.44 |
| **-0.9** | 97.11 | 45.9 | 71.5 | 0.5 |
| **-0.8** | 96.07 | 52.13 | 74.1 | 0.54 |
| **-0.7** | 95.22 | 57.84 | 76.53 | 0.57 |
| **-0.6** | 94.24 | 63.66 | 78.95 | 0.61 |
| **-0.5** | 92.96 | 68.88 | 80.92 | 0.64 |
| **-0.4** | 91.34 | 73.07 | 82.21 | 0.66 |
| **-0.3** | 89.95 | 76.82 | 83.39 | 0.67 |
| **-0.2** | 88.47 | 80.41 | 84.44 | 0.69 |
| **-0.1** | 87.09 | 83.55 | 85.32 | 0.71 |
| **0** | **85.28** | **86.25** | **85.77** | **0.72** |
| **0.1** | 83.42 | 88.27 | 85.84 | 0.72 |
| **0.2** | 81.84 | 90.44 | 86.14 | 0.73 |
| **0.3** | 79.76 | 92.07 | 85.91 | 0.72 |
| **0.4** | 77.51 | 93.57 | 85.54 | 0.72 |
| **0.5** | 74.92 | 94.96 | 84.94 | 0.71 |
| **0.6** | 72.18 | 96.14 | 84.16 | 0.7 |
| **0.7** | 68.54 | 97.01 | 82.78 | 0.68 |
| **0.8** | 64.9 | 97.64 | 81.27 | 0.66 |
| **0.9** | 60.44 | 98.25 | 79.34 | 0.63 |
| **1.0** | 47.87 | 98.68 | 73.27 | 0.54 |

**Table S16.** **Window size 13** (Kernel Parameters, t 2 g 0.1 j 1 c 10).

| **Threshold** | **Sensitivity (%)** | **Specificity (%)** | **Accuracy (%)** | **MCC** |
| --- | --- | --- | --- | --- |
| **-1.0** | 98.25 | 34.8 | 66.53 | 0.43 |
| **-0.9** | 97.46 | 43.08 | 70.27 | 0.48 |
| **-0.8** | 96.75 | 49.91 | 73.33 | 0.53 |
| **-0.7** | 95.79 | 55.81 | 75.8 | 0.56 |
| **-0.6** | 94.94 | 61.75 | 78.35 | 0.6 |
| **-0.5** | 93.51 | 67.66 | 80.58 | 0.63 |
| **-0.4** | 91.91 | 72.52 | 82.22 | 0.66 |
| **-0.3** | 90.52 | 76.18 | 83.35 | 0.67 |
| **-0.2** | 89.2 | 80.07 | 84.64 | 0.7 |
| **-0.1** | 87.37 | 83.87 | 85.62 | 0.71 |
| **0** | **85.7** | **86.52** | **86.11** | **0.72** |
| **0.1** | 83.83 | 89 | 86.42 | 0.73 |
| **0.2** | 81.86 | 91.07 | 86.46 | 0.73 |
| **0.3** | 79.8 | 92.68 | 86.24 | 0.73 |
| **0.4** | 77.2 | 94.39 | 85.8 | 0.73 |
| **0.5** | 75.07 | 95.57 | 85.32 | 0.72 |
| **0.6** | 72.24 | 96.56 | 84.4 | 0.71 |
| **0.7** | 69.13 | 97.5 | 83.32 | 0.69 |
| **0.8** | 65.1 | 98.23 | 81.66 | 0.67 |
| **0.9** | 59.89 | 98.72 | 79.3 | 0.64 |
| **1.0** | 46.11 | 98.96 | 72.54 | 0.53 |

**Table S17.** **Window size 15** (Kernel Parameters, t 2 g 0.1 j 1 c 10).

| **Threshold** | **Sensitivity (%)** | **Specificity (%)** | **Accuracy (%)** | **MCC** |
| --- | --- | --- | --- | --- |
| **-1.0** | 98.29 | 30.93 | 64.61 | 0.4 |
| **-0.9** | 97.4 | 39.62 | 68.51 | 0.45 |
| **-0.8** | 96.38 | 47.33 | 71.86 | 0.5 |
| **-0.7** | 95.61 | 53.67 | 74.64 | 0.54 |
| **-0.6** | 94.69 | 60.18 | 77.43 | 0.58 |
| **-0.5** | 93.41 | 66.14 | 79.78 | 0.62 |
| **-0.4** | 92.21 | 71.18 | 81.69 | 0.65 |
| **-0.3** | 90.71 | 76.14 | 83.43 | 0.68 |
| **-0.2** | 89.28 | 79.99 | 84.64 | 0.7 |
| **-0.1** | 87.65 | 83.51 | 85.58 | 0.71 |
| **0** | **85.36** | **86.31** | **85.84** | **0.72** |
| **0.1** | 83.61 | 88.9 | 86.26 | 0.73 |
| **0.2** | 81.78 | 91.32 | 86.55 | 0.73 |
| **0.3** | 79.6 | 93.21 | 86.41 | 0.73 |
| **0.4** | 77.41 | 94.73 | 86.07 | 0.73 |
| **0.5** | 75.11 | 95.87 | 85.49 | 0.73 |
| **0.6** | 71.79 | 96.73 | 84.26 | 0.71 |
| **0.7** | 68.8 | 97.48 | 83.14 | 0.69 |
| **0.8** | 64.29 | 98.01 | 81.15 | 0.66 |
| **0.9** | 58.94 | 98.47 | 78.7 | 0.62 |
| **1.0** | 44.01 | 98.9 | 71.45 | 0.51 |

**Table S18.** **Window size 17** (Kernel Parameters, t 2 g 0.1 j 1 c 10).

| **Threshold** | **Sensitivity (%)** | **Specificity (%)** | **Accuracy (%)** | **MCC** |
| --- | --- | --- | --- | --- |
| **-1.0** | 99.19 | 17.81 | 58.5 | 0.29 |
| **-0.9** | 98.46 | 27.21 | 62.83 | 0.37 |
| **-0.8** | 97.71 | 35.43 | 66.57 | 0.42 |
| **-0.7** | 96.57 | 44.25 | 70.41 | 0.48 |
| **-0.6** | 95.35 | 53.03 | 74.19 | 0.53 |
| **-0.5** | 93.9 | 60.52 | 77.21 | 0.58 |
| **-0.4** | 92.19 | 67.18 | 79.68 | 0.61 |
| **-0.3** | 89.92 | 73.6 | 81.76 | 0.64 |
| **-0.2** | 87.86 | 79.43 | 83.64 | 0.68 |
| **-0.1** | 85.68 | 84.61 | 85.14 | 0.7 |
| **0** | **83.69** | **87.99** | **85.84** | **0.72** |
| **0.1** | 80.95 | 90.86 | 85.9 | 0.72 |
| **0.2** | 78.36 | 92.94 | 85.65 | 0.72 |
| **0.3** | 75.57 | 94.65 | 85.11 | 0.72 |
| **0.4** | 72.21 | 95.95 | 84.08 | 0.7 |
| **0.5** | 69.21 | 96.83 | 83.02 | 0.69 |
| **0.6** | 65.64 | 97.41 | 81.52 | 0.66 |
| **0.7** | 61.64 | 97.94 | 79.79 | 0.64 |
| **0.8** | 55.6 | 98.57 | 77.08 | 0.6 |
| **0.9** | 48.02 | 98.93 | 73.47 | 0.55 |
| **1.0** | 34.9 | 99.29 | 67.09 | 0.45 |

**Table S19.** **Window size 19** (Kernel Parameters, t 2 g 0.1 j 1 c 10).

| **Threshold** | **Sensitivity (%)** | **Specificity (%)** | **Accuracy (%)** | **MCC** |
| --- | --- | --- | --- | --- |
| -1.0 | 98.88 | 25.65 | 62.27 | 0.36 |
| -0.9 | 98.23 | 35.41 | 66.82 | 0.43 |
| -0.8 | 97.44 | 42.87 | 70.16 | 0.48 |
| -0.7 | 96.48 | 50.48 | 73.48 | 0.53 |
| -0.6 | 95.55 | 57.94 | 76.75 | 0.58 |
| -0.5 | 94.43 | 64.21 | 79.32 | 0.62 |
| -0.4 | 93.19 | 70.43 | 81.81 | 0.65 |
| -0.3 | 91.78 | 75.72 | 83.75 | 0.68 |
| -0.2 | 89.97 | 80.37 | 85.17 | 0.71 |
| -0.1 | 88.08 | 84.64 | 86.36 | 0.73 |
| **0** | **86.13** | **88.37** | **87.25** | **0.75** |
| 0.1 | 83.53 | 90.69 | 87.11 | 0.74 |
| 0.2 | 81.55 | 92.56 | 87.05 | 0.75 |
| 0.3 | 79.15 | 94.1 | 86.62 | 0.74 |
| 0.4 | 76.65 | 95.49 | 86.07 | 0.73 |
| 0.5 | 73.87 | 96.48 | 85.18 | 0.72 |
| 0.6 | 70.61 | 97.4 | 84.01 | 0.71 |
| 0.7 | 66.38 | 98.23 | 82.3 | 0.68 |
| 0.8 | 61.66 | 98.72 | 80.19 | 0.65 |
| 0.9 | 55.66 | 99.15 | 77.41 | 0.61 |
| 1.0 | 39.84 | 99.47 | 69.65 | 0.49 |

**Table S20.** **Window size 21** (Kernel Parameters, t 2 g 0.1 j 1 c 10).

| **Threshold** | **Sensitivity (%)** | **Specificity (%)** | **Accuracy (%)** | **MCC** |
| --- | --- | --- | --- | --- |
| **-1.0** | 99.11 | 24.49 | 61.8 | 0.35 |
| **-0.9** | 98.5 | 33.17 | 65.84 | 0.42 |
| **-0.8** | 97.66 | 41.41 | 69.54 | 0.47 |
| **-0.7** | 96.85 | 49.32 | 73.09 | 0.52 |
| **-0.6** | 95.63 | 56.74 | 76.19 | 0.57 |
| **-0.5** | 94.43 | 63.39 | 78.91 | 0.61 |
| **-0.4** | 92.82 | 69.37 | 81.09 | 0.64 |
| **-0.3** | 91.13 | 74.98 | 83.05 | 0.67 |
| **-0.2** | 89.53 | 80.19 | 84.86 | 0.7 |
| **-0.1** | 87.57 | 84.38 | 85.97 | 0.72 |
| **0** | **85.52** | **87.33** | **86.43** | **0.73** |
| **0.1** | 83.14 | 90.12 | 86.63 | 0.73 |
| **0.2** | 80.7 | 92.68 | 86.69 | 0.74 |
| **0.3** | 78.12 | 94.51 | 86.32 | 0.74 |
| **0.4** | 75.76 | 95.53 | 85.65 | 0.73 |
| **0.5** | 72.85 | 96.6 | 84.72 | 0.71 |
| **0.6** | 69.57 | 97.36 | 83.46 | 0.7 |
| **0.7** | 65.87 | 98.15 | 82.01 | 0.68 |
| **0.8** | 61.3 | 98.6 | 79.95 | 0.65 |
| **0.9** | 54.75 | 99.1 | 76.92 | 0.6 |
| **1.0** | 39.33 | 99.41 | 69.37 | 0.48 |

**3]** Performance of SVM model developed using **amino acid sequence (binary pattern)** at different window lengths. SVM models were trained and tested on a dataset having **Real number of positive and negative data.** Bold row shows the performance of best SVM model.

**Table S21.** **Window size 15** (Kernel Parameters, t=2 (Radial) g=0.1 j=1 c=10).

--------------------------------------------------------------------------

**Thr SN SP ACC MCC**

---------------------------------------------------------------------------

-1.0 86.23 49.04 51.73 0.18

-0.9 78.46 63.70 64.77 0.22

-0.8 69.78 75.88 75.44 0.27

-0.7 61.34 85.18 83.45 0.31

-0.6 52.62 91.35 88.55 0.35

-0.5 44.01 95.25 91.54 0.38

**-0.4 36.67 97.44 93.05 0.40**

-0.3 30.24 98.70 93.75 0.41

-0.2 23.91 99.31 93.86 0.40

-0.1 18.78 99.63 93.79 0.37

0.0 14.52 99.78 93.62 0.33

0.1 11.00 99.88 93.45 0.30

0.2 8.36 99.92 93.30 0.26

0.3 6.37 99.94 93.18 0.23

0.4 4.46 99.95 93.05 0.19

0.5 3.06 99.97 92.96 0.16

0.6 2.07 99.98 92.90 0.13

0.7 1.34 99.99 92.86 0.10

0.8 0.78 100.00 92.82 0.08

0.9 0.42 100.00 92.80 0.06

1.0 0.27 100.00 92.79 0.05

SN- % Sensitivity; SP- % Specificity, ACC- % Accuracy, MCC- Matthew’s correlation coefficient

**Table S22.** **Window size 17** (Kernel Parameters, t=2 (Radial) g=0.1 j=5 c=1).

-----------------------------------------------------

**Thr SN SP ACC MCC**

-----------------------------------------------------

-1.0 89.54 43.15 46.51 0.17

-0.9 81.29 60.60 62.10 0.22

-0.8 72.28 75.46 75.23 0.28

-0.7 62.15 86.06 84.33 0.33

-0.6 51.51 92.56 89.59 0.37

-0.5 42.39 96.16 92.28 0.40

**-0.4 34.07 98.14 93.51 0.42**

-0.3 26.74 99.09 93.86 0.41

-0.2 21.27 99.54 93.88 0.39

-0.1 16.14 99.74 93.70 0.35

0.0 12.18 99.85 93.52 0.31

0.1 9.12 99.89 93.33 0.27

0.2 6.52 99.92 93.17 0.23

0.3 4.59 99.94 93.05 0.19

0.4 3.27 99.96 92.97 0.16

0.5 2.03 99.98 92.90 0.13

0.6 1.47 99.99 92.87 0.11

0.7 0.96 100.00 92.84 0.09

0.8 0.54 100.00 92.81 0.07

0.9 0.29 100.00 92.79 0.05

1.0 0.13 100.00 92.78 0.03

**Table S23.** **Window size 19** (Kernel Parameters, t=2 (Radial) g=0.1 j=1 c=100).

-------------------------------------------

**Thr SN SP ACC MCC**

---------------------------------------------

-1.0 91.97 37.10 41.06 0.16

-0.9 84.16 57.82 59.73 0.22

-0.8 73.32 75.23 75.10 0.28

-0.7 61.65 87.06 85.22 0.34

-0.6 49.85 93.90 90.72 0.39

**-0.5 39.50 97.27 93.10 0.42**

-0.4 30.39 98.79 93.84 0.42

-0.3 22.95 99.45 93.92 0.40

-0.2 16.89 99.73 93.74 0.36

-0.1 12.15 99.84 93.50 0.31

0.0 8.61 99.89 93.30 0.26

0.1 6.08 99.92 93.14 0.22

0.2 4.17 99.96 93.03 0.18

0.3 2.64 99.98 92.94 0.15

0.4 1.74 99.99 92.89 0.12

0.5 1.19 100.00 92.86 0.10

0.6 0.65 100.00 92.82 0.08

0.7 0.46 100.00 92.81 0.07

0.8 0.25 100.00 92.79 0.05

0.9 0.15 100.00 92.78 0.04

1.0 0.04 100.00 92.78 0.02

**4]** Performance of SVM model developed using **evolutionary information in the form of PSSM profile generated by PSI-BLAST** for different window lengths. SVM models were trained and tested on a dataset having **Real number of positive and negative data**. Bold row shows the performance of best SVM model.

**Table S24.** **Window size 15** (Kernel Parameters, t=2 (Radial) g=0.1 j=1 c=10).

--------------------------------------------

**Thr SN SP ACC MCC**

-------------------------------------------

-1.0 88.69 79.32 80.03 0.41

-0.9 85.68 87.55 87.41 0.50

-0.8 83.36 91.42 90.81 0.57

-0.7 81.29 94.08 93.11 0.62

-0.6 79.54 95.85 94.61 0.67

-0.5 77.32 97.01 95.51 0.70

-0.4 75.47 97.79 96.10 0.72

**-0.3 74.07 98.32 96.48 0.74**

-0.2 72.26 98.67 96.67 0.75

-0.1 70.55 98.92 96.77 0.75

0.0 68.54 99.11 96.79 0.75

0.1 67.03 99.26 96.81 0.75

0.2 65.18 99.37 96.78 0.75

0.3 63.27 99.46 96.72 0.74

0.4 60.97 99.53 96.61 0.73

0.5 58.45 99.59 96.47 0.72

0.6 55.48 99.65 96.30 0.70

0.7 51.82 99.71 96.08 0.68

0.8 48.28 99.75 95.85 0.66

0.9 42.87 99.81 95.50 0.62

1.0 27.58 99.88 94.40 0.50

**Table S25.** **Window size 17** (Kernel Parameters, t=2 (Radial) g=0.1 j=1 c=10).

---------------------------------------------

**Thr SN SP ACC MCC**

---------------------------------------------

-1.0 89.93 77.64 78.57 0.40

-0.9 86.52 86.85 86.83 0.50

-0.8 83.93 91.17 90.62 0.56

-0.7 81.70 93.96 93.03 0.62

-0.6 79.91 95.86 94.65 0.67

-0.5 77.85 97.07 95.61 0.71

-0.4 75.96 97.87 96.21 0.73

-0.3 73.81 98.33 96.47 0.74

**-0.2 72.14 98.69 96.68 0.75**

-0.1 70.45 98.93 96.77 0.75

0.0 68.86 99.11 96.82 0.75

0.1 66.97 99.26 96.81 0.75

0.2 64.98 99.36 96.76 0.75

0.3 63.07 99.46 96.70 0.74

0.4 60.77 99.52 96.59 0.73

0.5 57.78 99.60 96.43 0.71

0.6 55.05 99.66 96.28 0.70

0.7 51.92 99.70 96.08 0.68

0.8 47.79 99.76 95.82 0.65

0.9 42.12 99.83 95.45 0.62

1.0 26.03 99.91 94.31 0.48

**Table S26.** **Window size 19** (Kernel Parameters, t=2 (Radial) g=0.1 j=1 c=10).

--------------------------------------------

**Thr SN SP ACC MCC**

--------------------------------------------

-1.0 90.81 76.32 77.42 0.39

-0.9 87.63 86.21 86.32 0.49

-0.8 84.67 90.91 90.44 0.56

-0.7 82.00 93.98 93.07 0.62

-0.6 79.87 95.89 94.68 0.67

-0.5 77.87 97.12 95.66 0.71

-0.4 75.84 97.93 96.25 0.73

-0.3 73.93 98.43 96.57 0.75

**-0.2 72.14 98.77 96.75 0.76**

-0.1 70.37 98.98 96.81 0.76

0.0 68.44 99.11 96.79 0.75

0.1 66.54 99.26 96.78 0.75

0.2 64.59 99.37 96.73 0.74

0.3 62.60 99.45 96.66 0.74

0.4 60.18 99.53 96.55 0.73

0.5 57.23 99.60 96.39 0.71

0.6 54.34 99.67 96.23 0.69

0.7 50.50 99.73 96.00 0.67

0.8 46.49 99.78 95.74 0.65

0.9 40.88 99.82 95.36 0.61

1.0 24.28 99.91 94.18 0.47

**Table S27. Performance of BLAST on 6 independent proteins**

We obtained the below mentioned 6 NAD binding proteins from the PDB which were not used in the training or model building process. NAD interacting residues (NIRs) are known for these proteins. For BLAST we used our database of 195 NAD binding proteins. NCBI-BLAST was run locally for each query protein sequence e.g. 2g5c_B against database**.** Top hit was considered for prediction and a separate Global pair-wise alignment was performed using EMBOSS-needle at EBI between query and top hit. Alignment details (BLAST as well as separate pair-wise Global alignment) of each protein are shown here. Each NAD interacting residues is mapped on the alignment. Overlapped True positive residues are highlighted, counted and equation for the calculation of Sensitivity and PPV is also mentioned.

| **Query** | **BLAST Hit** | **E-value** | **NAD interacting residues in query** | **NAD interacting residues in target** | **Blast Local Alignment** | | | **Global alignment**  **[EBI-Emboss Needle; Default parameters]** | | |
| --- | --- | --- | --- | --- | --- | --- | --- | --- | --- | --- |
|  |  |  |  |  | **TP** | **Sen %** | **PPV %** | **TP** | **Sen %** | **PPV %** |
| **2g5c_B** | 2pv7_B | 5e-05 | 26 | 24 | 12 | 46.2 | 50 | 12 | 46.2 | 50 |
| **1kqn_A** | 1k4m_A | 6e-06 | 31 | 30 | 19 | 61.3 | 63.3 | 21 | 67.7 | 70 |
| **2qjo_B** | 1m8k_A | 1e-09 | 29 | 23 | 12 | 41.4 | 52.2 | 16 | 55.2 | 69.6 |
| **4mdh_A** | 1guz_A | 3e-12 | 28 | 33 | 17 | 60.7 | 51.5 | 24 | 85.7 | 72.7 |
| **1p1h_C** | 3cin_A | 2e-14 | 35 | 35 | 16 | 45.7 | 45.7 | 23 | 65.7 | 65.7 |
| **2d37_A** | 1rz1_A | 1e-16 | 14 | 14 | 06 | 42.9 | 42.9 | 09 | 64.3 | 64.3 |
| **Average** |  |  |  |  |  | **49.7** | **50.9** |  | **64.1** | **65.4** |

**TP** = NAD interacting residues common in both

**Sen % =** % Sensitivity

**PPV %** = % Probability of correct positive prediction

**Definitions:**

- NIRs = NAD interacting Residues
- True Positive= number of common NIRs (highlighted yellow); NIRs which were actually positive and predicted as positive in target alignment
- False Negative= NIRs which were actually positive but predicted as negative in target alignment
- True Negative= NIRs which were actually negative and predicted as negative in target alignment
- False Positive= NIRs which were actually negative but predicted as positive in target alignment
- (true positive + false negative)= actual NIRs in query
- (true positive + false positive)= total positive prediction in query = total NIRs in target
- **Calculation of Sensitivity:**

Sensitivity = True Positive / (true positive + false negative)

Or sensitivity = number of common residues/NIRs in query

**% sensitivity** = (12/26)*100 = **46.2**

- **Calculation of Probability of correct positive prediction (PPV):**

PPV = True Positive/ (true positive + false positive)

= True Positive/ total positive prediction

= number of common residues/NIRs in target

**% PPV** = (12/24)*100 = **50**

***Some alignments are shown in smaller zoom to adjust into the same page. Please increase the zoom to visualize more clearly.***

**1]** Query= 2g5c_B (280 letters)

>2g5c_B

QNVLIvgVgfXGGSFAKSLRRSGFKGKIYGydinPEsISKAVDLGIIDEGTTSIAKVEDFSPDFVXLsspvRtfREiAKKLSYILSEDATVTDqgsVKGKLVYDLENILGKRFVGGhPIAGteKsgVEYSLDNLYEGKKVILTPTKKTDKKRLKLVKRVWEDVGGVVEYXSPELHDYVFGVVSHLPHAVAFALVDTLIHXSTPEVDLFKYPGGGFKDFTRIAKSdPIXWRDiFLENKENVXKAIEGFEKSLNHLKELIVREAEEELVEYLKEVKIKRXEI

Top Hit= 2pv7_B; Length= 280

>2pv7_B

GFKTINSDIHKIVIvgGygklGGLFARYLRASGYPISILdrEDwAVAESILANADVVIVsvpiNlTLEtIErLKPYLTENXLLADLtsVKREPLAKXLEVHTGAVLGLhPXFgADIASXAKQVVVRCDGRFPERYEWLLEQIQIWGAKIYQTNATEHDHNXTYIQALRHFSTFANGLHLSKQPINLANLLALSSPIYRLELAXIGRLFAqdAElYADIIXDKSENLAVIETLKQTYDEALTFFENNDRQGFIDAFHKVRDWFGDYSEQFLKESRQLLQQy

Score = 37.4 bits (85), Expect = 5e-05

Identities = 30/121 (24%), Positives = 52/121 (42%), Gaps = 18/121 (14%)

Query: 3 VLIVGVGFXGGSFAKSLRRSGFKGKIYGYDINPESISKAVDLGIIDEGTTSIAKVEDFSP 62

V++ G G GG FA+ LR SG+ + I+D ++A+ +

Sbjct: 13 VIVGGYGKLGGLFARYLRASGY------------------PISILDREDWAVAESILANA 54

Query: 63 DFVXLSSPVRTFREIAKKLSYILSEDATVTDQGSVKGKLVYDLENILGKRFVGGHPIAGT 122

D V +S P+ E ++L L+E+ + D SVK + + + +G HP G

Sbjct: 55 DVVIVSVPINLTLETIERLKPYLTENXLLADLTSVKREPLAKXLEVHTGAVLGLHPXFGA 114

Query: 123 E 123

+

Sbjct: 115 D 115

**actual NIRs in query** (small red) = 26

**NIRs in target** (small green) = 24

**Common NIRs (True positive residues in query which were actually NIRs and also predicted as NIRs in alignment)** (highlighted in yellow) = 12

**Global alignment by Needle [EBI-EMBOSS]**

Length: 335

# Identity: 64/335 (19.1%) # Similarity: 113/335 (33.7%) # Gaps: 110/335 (32.8%) # Score: 119.0

#=======================================

2g5c_B 1 QNVLIVGVGFXGGSFAKSLRRSGFKGKIYGYDINPESISK 40

:.|::.|.|. ||.||:.||.||:

2pv7_B 1 GFKTINSDIHKIVIVGGYGKLGGLFARYLRASGY---------------- 34

2g5c_B 41 AVDLGIIDEGTTSIAKVEDFSPDFVXLSSPVRTFREIAKKLSYILSEDAT 90

.:.|:|....::|:....:.|.| :|.|:....|..::|...|:|:..

2pv7_B 35 --PISILDREDWAVAESILANADVVIVSVPINLTLETIERLKPYLTENXL 82

2g5c_B 91 VTDQGSVKGK-LVYDLENILGKRFVGGHPIAGTEKSGVEYSLDNLYEGKK 139

:.|..|||.: |...||...| ..:|.||..|. |.....|:

2pv7_B 83 LADLTSVKREPLAKXLEVHTG-AVLGLHPXFGA---------DIASXAKQ 122

2g5c_B 140 VILTPTKKTDKKRLKLVK--RVWEDVGGVVEYXSPELHDYVFGVVSHLPH 187

|::....:..::...|:: ::| |..:.. :...||:....:..|.|

2pv7_B 123 VVVRCDGRFPERYEWLLEQIQIW---GAKIYQTNATEHDHNXTYIQALRH 169

2g5c_B 188 AVAFALVDTLIHXSTPEVDLFKYPGGGFKDFTRIAKSDPI---------- 227

...|| ..:| |...::|... :|.|.||

2pv7_B 170 FSTFA---NGLHLSKQPINLANL----------LALSSPIYRLELAXIGR 206

2g5c_B 228 -------XWRDIFLENKENVX----------KAIEGFEKS---------- 250

:.||..:..||: :|:..||.:

2pv7_B 207 LFAQDAELYADIIXDKSENLAVIETLKQTYDEALTFFENNDRQGFIDAFH 256

2g5c_B 251 -----LNHLKELIVREAEEELVEYLKEVKIKRXEI 280

.....|..::|:.:.|.:|

2pv7_B 257 KVRDWFGDYSEQFLKESRQLLQQY 280

Number of Common residues (highlighted in yellow) =12

**2]** Query= 1kqn_A (232 letters)

>1kqn_A

KTEVVLLacgsfNPITNmhLRlFELAKDYMNGTGRYTVVKGIISPvGDAyKkKGLIPAYHRVIMAELATKNSKWVEVDTWeSLQKEwKetLKVLRHHQEKLEAAVPKVKLLcgAdlLEsFAVPNlwKSEdITQIVANYGLICVtrAGNDAQKFIYESDVLWKHRSNIHVVNeWIANdIssTKIRRALRRGQSIRYLVPDLVQEYIEKHNLYSSESEDRNAGVILApLQRnTA

Top Hit= 1k4m_A; Length = 213

>1k4m_A MKSLQALfggtfDPVhYghLKpVETLANLIGLTRVTIIPnNVpphrPQPEANSVQRKHMLELAIADKPLFTLDEReLKRNAPSytAQTLKEWRQEQGPDVPLAfIigQdsLLtFPtwyEYETILDNAHLIVcRrPGYPLEMAQPQYQQWLEDHLTHNPEDLHLQPAGKIYLAETPWfNIsATIIRERLQNGESCEDLLPEPVLTYINQQGLYR

Score = 40.0 bits (92), Expect = 6e-06

Identities = 53/216 (24%), Positives = 91/216 (42%), Gaps = 27/216 (12%)

Query: 10 GSFNPITNMHLRLFELAKDYMNGTGRYTVVKGIISPVGDAYKKKGLIPAYHRVIMAELAT 69

G+F+P+ HL+ E + + G R T++ + P ++ + + R M ELA

Sbjct: 10 GTFDPVHYGHLKPVETLANLI-GLTRVTIIPNNVPP----HRPQPEANSVQRKHMLELAI 64

Query: 70 KNSKWVEVDTWESLQKEWKETLKVLRHHQEKLEAAVPKVKLLCGADLLESFAVPNLWKSE 129

+ +D E + T + L+ +++ VP + + G D L +F P ++ E

Sbjct: 65 ADKPLFTLDERELKRNAPSYTAQTLKEWRQEQGPDVP-LAFIIGQDSLLTF--PTWYEYE 121

Query: 130 DITQIVANYGLICVTRAGN-----DAQKFIYESDVLWKHRSNIHVV---------NEWIA 175

I N LI R G Q + D L + ++H+ W

Sbjct: 122 TILD---NAHLIVCRRPGYPLEMAQPQYQQWLEDHLTHNPEDLHLQPAGKIYLAETPWF- 177

Query: 176 NDISSTKIRRALRRGQSIRYLVPDLVQEYIEKHNLY 211

+IS+T IR L+ G+S L+P+ V YI + LY

Sbjct: 178 -NISATIIRERLQNGESCEDLLPEPVLTYINQQGLY 212

**actual NIRs in query** (small red) = 31

**NIRs in target** (small green)= 30

**Common NIRs (True positive residues in query which were actually NIRs and also predicted as NIRs in alignment)** (highlighted in yellow) =19

**Global alignment by Needle [EBI-EMBOSS]**

# Length: 248

# Identity: 54/248 (21.8%) # Similarity: 96/248 (38.7%) # Gaps: 51/248 (20.6%)# Score: 112.0

#=======================================

1kqn_A 1 KTEVVLLACGSFNPITNMHLRLFELAKDYMNGTGRYTVVKGIISPVGDAY 50

...:..|..|:|:|:...||:..|...:.: |..|.|::...:.| :

1k4m_A 1 MKSLQALFGGTFDPVHYGHLKPVETLANLI-GLTRVTIIPNNVPP----H 45

1kqn_A 51 KKKGLIPAYHRVIMAELATKNSKWVEVDTWESLQKEWKETLKVLRHHQEK 100

:.:....:..|..|.|||..:.....:|..|..:.....|.:.|:..:::

1k4m_A 46 RPQPEANSVQRKHMLELAIADKPLFTLDERELKRNAPSYTAQTLKEWRQE 95

1kqn_A 101 LEAAVPKVKLLCGADLLESFAVPNLWKSEDITQIVANYGLICVTRAG--- 147

....|| :..:.|.|.|.:| |..::.| .|:.|..||...|.|

1k4m_A 96 QGPDVP-LAFIIGQDSLLTF--PTWYEYE---TILDNAHLIVCRRPGYPL 139

1kqn_A 148 ----NDAQKFIYESDVLWKHRSNIHV---------VNEWIANDISSTKIR 184

...|::: .|.|..:..::|: ...|. :||:|.||

1k4m_A 140 EMAQPQYQQWL--EDHLTHNPEDLHLQPAGKIYLAETPWF--NISATIIR 185

1kqn_A 185 RALRRGQSIRYLVPDLVQEYIEKHNLYSSESEDRNAGVILAPLQRNTA 232

..|:.|:|...|:|:.|..||.:..||.

1k4m_A 186 ERLQNGESCEDLLPEPVLTYINQQGLYR 213

**Number of Common residues (highlighted in yellow) =21**

**3]** Query= 2qjo_B (336 letters)

>2qjo_B

KYQYGIyigrfQPFhLghLRtLNLALEKAEQVIIILgsHRVAADTrNPWRSPERMAMIEACLSPQILKRVHFLTVRdWlYSdNLwLAAVQQQVLKITGGSNSVVVLghRkDAssyylNLFPQWDYLEtGhyPDfSsTAIRGAYFEGKEGDYLDKVPPAIADYLQTFQKSERYIALCDEYQFLQAYKQAWATAPYAPTFITTDAVVVQAGHVLMVRRQAKPGLGLIALPGGFIKQNETLVEGMLRELKEETRLKVPLPVLRGSIVDSHVFDAPGRSLRGRTITHAYFIQLPGGELPAVKGGDDAQKAWWMSLADLYAQEEQIYEDHFQIIQHFVSKV

Top Hit= 1m8k_A; Length = 169

>1m8k_A

TMRGLlvgrMQPFhRGALQvIKSILEEVDELIICIgsAQLSHSIrDPFTAGERVMMLTKALSENGIPASRYYIIPVQdiECnALwVGHIKMLTPPFDRVYsGnPlvQRlFSEDGYEVTApPLfyRdRYsGTEVRRRMLDDGDWRSLLPESVVEVIDEINGVERIKHLAK

Score = 53.1 bits (126), Expect = 1e-09

Identities = 27/88 (30%), Positives = 53/88 (60%), Gaps = 3/88 (3%)

Query: 5 GIYIGRFQPFHLGHLRTLNLALEKAEQVIIILGSHRVAADTRNPWRSPERMAMIEACLSP 64

G+ +GR QPFH G L+ + LE+ +++II +GS +++ R+P+ + ER+ M+ LS

Sbjct: 4 GLLVGRMQPFHRGALQVIKSILEEVDELIICIGSAQLSHSIRDPFTAGERVMMLTKALSE 63

Query: 65 QIL--KRVHFLTVRDWLYSDNLWLAAVQ 90

+ R + + V+D + + LW+ ++

Sbjct: 64 NGIPASRYYIIPVQD-IECNALWVGHIK 90

**actual NIRs in query** (small red) = 29

**NIRs in target** (small green) = 23

**Common NIRs (True positive residues in query which were actually NIRs and also predicted as NIRs in alignment)** (highlighted in yellow) = 12

**Global alignment by Needle [EBI-EMBOSS]**

Length: 359

# Identity:45/359 (12.5%)# Similarity: 83/359 (23.1%)# Gaps: 213/359 (59.3%)# Score: 142.0

#=======================================

2qjo_B 1 KYQYGIYIGRFQPFHLGHLRTLNLALEKAEQVIIILGSHRVAADTRNPWR 50

...|:.:||.||||.|.|:.:...||:.:::||.:||.:::...|:|:.

1m8k_A 1 TMRGLLVGRMQPFHRGALQVIKSILEEVDELIICIGSAQLSHSIRDPFT 49

2qjo_B 51 SPERMAMIEACLSPQIL--KRVHFLTVRDWLYSDNLWLAAVQQQVLKITG 98

:.||:.|:...||...: .|.:.:.|:| :..:.||

1m8k_A 50 AGERVMMLTKALSENGIPASRYYIIPVQD-IECNALW------------- 85

2qjo_B 99 GSNSVVVLGHRKDASSYYLNLFPQWDYLETGH------------------ 130

:||.| .|.|.:|.:.:|:

1m8k_A 86 -------VGHIK-------MLTPPFDRVYSGNPLVQRLFSEDGYEVTAPP 121

2qjo_B 131 --YPD-FSSTAIRGAYFEGKEGDYLDKVPPAIADYLQTFQKSERYIALCD 177

|.| :|.|.:|....: :||:...:|.::.:.:......||...|..

1m8k_A 122 LFYRDRYSGTEVRRRMLD--DGDWRSLLPESVVEVIDEINGVERIKHLAK 169

2qjo_B 178 EYQFLQAYKQAWATAPYAPTFITTDAVVVQAGHVLMVRRQAKPGLGLIAL 227

1m8k_A 170 169

2qjo_B 228 PGGFIKQNETLVEGMLRELKEETRLKVPLPVLRGSIVDSHVFDAPGRSLR 277

1m8k_A 170 169

2qjo_B 278 GRTITHAYFIQLPGGELPAVKGGDDAQKAWWMSLADLYAQEEQIYEDHFQ 327

1m8k_A 170 169

2qjo_B 328 IIQHFVSKV 336

1m8k_A 170 169

Number of Common residues (highlighted in yellow)=16

**4]** Query= 4mdh_A(333 letters)

>4mdh_A

SEPIRVLVtgAagqiAYSLLYSIGNGSVFGKDQPIILVLldiTPmMGVLDGVLMELQDCALPLLKDVIATDKEEIAFKDLDVAILvgsMprRDGMERKDLlKANVKiFKCqGAALDKYAKKSVKVIVvgnPaNTNCLTASKSAPSIPKENFSClTRlDHNRAKAQIALKLGVTSDDVKNVIIWGNhSSTQYPDVNHAKVKLQAKEVGVYEAVKDDSWLKGEFITTVQQRGAAVIKARKLssAMSaAKAICDHVRDIWFGTPEGEFVSMGIISDGNSYGVPDDLLYSFPVTIKDKTWKIVEGLPINDFSREKMDLTAKELAEEKETAFEFLSSA

Top Hit= 1guz_A; Length = 305

>1guz_A

MKITVigagnvGATTAFRLAEKQLARELVLldvvEGiPQGKALDMYESGPVGLFDTKVTGSNDyADTANSDIVIItaglpRKPGMTREDLLMKnAGiVKevTDNIMKHSKNPIIIVvsnPlDIMTHVAWVRSGLPKERVIGmaGVlDAArFRSFIAMELGVSMQDINACVLGGhGDAMVPVVKYTTVAGIPISDLLPAETIDKLVERTRNGGAEIVEHLKQGsaFYApASSVVEMVESIVLDRKRVLPCAVGLEGQYGIDKTFVGVPVKLGRNGVEQIYEINLDQADLDLLQKSAKIVDENCKML

Score = 61.6 bits (148), Expect = 3e-12

Identities = 63/227 (27%), Positives = 102/227 (44%), Gaps = 22/227 (9%)

Query: 37 LVLLDITPMMGVLDGVLMELQDCALPLLKDVIATDKEEIA-FKDLDVAILVGSMPRRDGM 95

LVLLD+ G+ G +++ + L D T + A + D+ I+ +PR+ GM

Sbjct: 28 LVLLDVVE--GIPQGKALDMYESGPVGLFDTKVTGSNDYADTANSDIVIITAGLPRKPGM 85

Query: 96 ERKDLLKANVKIFKCQGAALDKYAKKSVKVIVVGNPANTNCLTASKSAPSIPKENFSCLT 155

R+DLL N I K + K++K + +IVV NP + A + +PKE +

Sbjct: 86 TREDLLMKNAGIVKEVTDNIMKHSKNPI-IIVVSNPLDIMTHVAWVRS-GLPKERVIGMA 143

Query: 156 R-LDHNRAKAQIALKLGVTSDDVKNVIIWGNHSSTQYPDVNHAKVKLQAKEVGVYEAVKD 214

LD R ++ IA++LGV+ D+ N + G H P V + V +

Sbjct: 144 GVLDAARFRSFIAMELGVSMQDI-NACVLGGHGDAMVPVVKYTTV----------AGIPI 192

Query: 215 DSWLKGEFITTVQQR----GAAVIKARKLSSAMSA-AKAICDHVRDI 256

L E I + +R GA +++ K SA A A ++ + V I

Sbjct: 193 SDLLPAETIDKLVERTRNGGAEIVEHLKQGSAFYAPASSVVEMVESI 239

**actual NIRs in query** (small red) = 28

**NIRs in target** (small green) = 33

**Common NIRs (True positive residues in query which were actually NIRs and also predicted as NIRs in alignment)** (highlighted in yellow) = 17

**Global alignment by Needle [EBI-EMBOSS]**

# Length: 361

# Identity: 86/361 (23.8%) # Similarity: 138/361 (38.2%)# Gaps: 84/361 (23.3%) # Score: 171.0

4mdh_A 1 SEPIRVLVTGAAGQIAYSLLYSIGNGSVFGKDQPIILVLLDITPMMGVLD 50

:::.|.| ||.:..:..:.:.. |.....|||||: :.|:..

1guz_A 1 MKITVIG-AGNVGATTAFRLAE-----KQLARELVLLDV--VEGIPQ 39

4mdh_A 51 GVLMELQDCALPLLKDVIATDKEEIA-FKDLDVAILVGSMPRRDGMERKD 99

|..:::.:.....|.|...|...:.| ..:.|:.|:...:||:.||.|:|

1guz_A 40 GKALDMYESGPVGLFDTKVTGSNDYADTANSDIVIITAGLPRKPGMTRED 89

4mdh_A 100 LLKANVKIFKCQGAALDKYAKKSVKVIVVGNPANTNCLTASKSAPSIPKE 149

||..|..|.|.....:.|::|..: :|||.||.:.....|...: .:|||

1guz_A 90 LLMKNAGIVKEVTDNIMKHSKNPI-IIVVSNPLDIMTHVAWVRS-GLPKE 137

4mdh_A 150 NFSCLTR-LDHNRAKAQIALKLGVTSDDVKNVIIWGNHSSTQYPDVNHAK 198

....:.. ||..|.::.||::|||:..|: |..:.|.|.....|.|.:..

1guz_A 138 RVIGMAGVLDAARFRSFIAMELGVSMQDI-NACVLGGHGDAMVPVVKYTT 186

4mdh_A 199 VKLQAKEVGVYEAVKDDSWLKGEFITTVQQR----GAAVIKARKLSSAMS 244

| ..:.....|..|.|..:.:| ||.:::..|..||..

1guz_A 187 V----------AGIPISDLLPAETIDKLVERTRNGGAEIVEHLKQGSAFY 226

4mdh_A 245 A-AKAICDHVRDIWFGTP---------EGEFVSMGIISDGNSYGVPDDL- 283

| |.::.:.|..|..... ||:: || |....|||..|

1guz_A 227 APASSVVEMVESIVLDRKRVLPCAVGLEGQY---GI--DKTFVGVPVKLG 271

4mdh_A 284 ------LYSFPVTIKD-----KTWKIVEGLPINDFSREKMDLTAKELAEE 322

:|...:...| |:.||| |...|.|

1guz_A 272 RNGVEQIYEINLDQADLDLLQKSAKIV-------------DENCKML 305

4mdh_A 323 KETAFEFLSSA 333

1guz_A 306 305

Number of Common residues (highlighted in yellow) =24

**5]** Query= 1p1h_C(517 letters)

>1p1h_C

TSVKVVTDKCTYKDNELLTKYSYENAVVTKTASGRFDVTPTVQDYVFKLDLKKPEKLGIMLigLGgnnGSTLVASVLANKHNVEFQTKEGVKQPNYFGSMTQCSTLKLGIDAEGNDVYAPFNSLLPMVSPNDFVVSGWdiNNADLYEAMQrSQVLEYDLQQRLKAKMSLVKPLPsIyYPDFiAANqDErANNCINLDEKGNVTTRGKWTHLQRIRRDIQNFKEENALDKVIVLwtanteRYVEVSPGVNDTMENLLQSIKNDHEEIApSTIfAAASILEGVPYINGspQNTFVPGLVQLAEHEGTFIAGDDlKsGQTKLKSVLAQFLVDAGIKPVSIASYNHLGnndGYnLSAPKQFRSkEISKSSVIDDIIASNDILYNDKLGKKVDHCIVIKYMKPVGdSKVAMDEYYSELMLGGHNRISIHNVCEdsLLaTPLIIDLLVMTEFCTRVSYKKVDKFENFYPVLTFLSYWLkAPLTRPGFHPVNGLNKQRTALENFLRLLIGLPSQNELRFEERLL

Top Hit= 3cin_A; Length = 382

>3cin_A

HMVKVLILgQgyvASTFVAGLEKLRKGEIEPYGVPLARELPIGFEDIKIVGSydvdRAkIGKKLSEVVKQyWNDVDSLTSDPEIRKGVhLGsvRNLPiEAEGLEDSMTLKEAVDTLVKEWTELDPDVIVNtcttEAFVPFGNKEDLLKAIENNDKERLTaTQVyAYAAALYANKRGGAAFVNvipTFIANDPAFVELAKENNLVVFGDdgAtGATPFTADVLSHLAQRNRYVKDVAQFNIGGnmdfLALTDDGKNKSKEFTKSSIVKDILGYDAPHYIKPTGYLEPLGDKkFIAIHIEYVSFNGATDELMINGRINdsPAlGGLLVDLVRLGKIALDRKEFGTVYPVNAFYMkNPGPAEEKNIPRIIAYEKMRIWAGLKPKW

Score = 69.7 bits (169), Expect = 2e-14

Identities = 68/257 (26%), Positives = 112/257 (43%), Gaps = 35/257 (13%)

Query: 222 KEENALDKVIVLWTANTERYVEVSPGVNDTMENLLQSIKN-DHEEIAPSTIFAAASILE- 279

KE LD +++ T TE +V E+LL++I+N D E + + ++A A+ L

Sbjct: 118 KEWTELDPDVIVNTCTTEAFVPFG-----NKEDLLKAIENNDKERLTATQVYAYAAALYA 172

Query: 280 ----GVPYINGSPQNTFV---PGLVQLAEHEGTFIAGDDLKSGQTKLKSVLAQFLVDAGI 332

G ++N P TF+ P V+LA+ + GDD +G T + + L

Sbjct: 173 NKRGGAAFVNVIP--TFIANDPAFVELAKENNLVVFGDDGATGATPFTADVLSHLAQRNR 230

Query: 333 KPVSIASYNHLGNNDGYNLSAPKQFRSKEISKSSVIDDIIASNDILYNDKLGKKVDHCIV 392

+A +N GN D L+ + +SKE +KSS++ DI+ + Y G

Sbjct: 231 YVKDVAQFNIGGNMDFLALTDDGKNKSKEFTKSSIVKDILGYDAPHYIKPTG-------- 282

Query: 393 IKYMKPVGDSKVAMDEYYSELMLGGHNRISIHNVCEDSLLATPLIIDLLVMTEFCTRVSY 452

Y++P+GD K G + + I+ DS L++DL+ R+

Sbjct: 283 --YLEPLGDKKFIAIHIEYVSFNGATDELMINGRINDSPALGGLLVDLV-------RLGK 333

Query: 453 KKVDK--FENFYPVLTF 467

+D+ F YPV F

Sbjct: 334 IALDRKEFGTVYPVNAF 350

**actual NIRs in query** (small red) = 35

**NIRs in target** (small green) = 35

**Common NIRs (True positive residues in query which were actually NIRs and also predicted as NIRs in alignment)** (highlighted in yellow) = 16

**Global alignment by Needle [EBI-EMBOSS]**

# Length:540 # Identity: 109/540 (20.2%)# Similarity:187/540(34.6%) # Gaps: 181/540(33.5%) # Score: 211.5

1p1h_C 1 TSVKVVTDKCTYKDNELLTKYSYENAVVTKTASGRFDVTPTVQDYVFKLD 50

3cin_A 1 0

1p1h_C 51 LKKPEKLGIMLIGLGGNNGSTLVASVLANKHNVEFQTKEGVKQPNYFGSM 100

..:.::::| .|...||.||.: .:.::|..:| :|

3cin_A 1 HMVKVLILG-QGYVASTFVAGL--------EKLRKGEIEP--YG-- 33

1p1h_C 101 TQCSTLKLGIDAEGNDVYAPFNSLLPMVSPNDFVVSGWDINNADLYEAMQ 150

.|....||:...:..:|..:|::.|.:.:.:

3cin_A 34 ------------------VPLARELPIGFEDIKIVGSYDVDRAKIGKKL- 64

1p1h_C 151 RSQVLEYDLQQRLKAKMSLVKPLPSIYYPDFIAANQDERANNCINLDEKG 200

|:|::. |:.|..:...|......::|....

3cin_A 65 -SEVVKQ-------------------YWNDVDSLTSDPEIRKGVHLGSVR 94

1p1h_C 201 NVTTRGKWTHLQRIRRDIQN--FKEENALDKVIVLWTANTERYVEVSPGV 248

|:....:........::..: .||...||..:::.|..||.:|...

3cin_A 95 NLPIEAEGLEDSMTLKEAVDTLVKEWTELDPDVIVNTCTTEAFVPFG--- 141

1p1h_C 249 NDTMENLLQSIK-NDHEEIAPSTIFAAASIL-----EGVPYINGSPQNTF 292

..|:||::|: ||.|.:..:.::|.|:.| .|..::|..| ||

3cin_A 142 --NKEDLLKAIENNDKERLTATQVYAYAAALYANKRGGAAFVNVIP--TF 187

1p1h_C 293 V---PGLVQLAEHEGTFIAGDDLKSGQTKLKSVLAQFLVDAGIKPVSIAS 339

: |..|:||:.....:.|||..:|.|...:.:...|.........:|.

3cin_A 188 IANDPAFVELAKENNLVVFGDDGATGATPFTADVLSHLAQRNRYVKDVAQ 237

1p1h_C 340 YNHLGNNDGYNLSAPKQFRSKEISKSSVIDDIIASNDILYNDKLGKKVDH 389

:|..||.|...|:...:.:|||.:|||::.|| ||....|

3cin_A 238 FNIGGNMDFLALTDDGKNKSKEFTKSSIVKDI-----------LGYDAPH 276

1p1h_C 390 CI-VIKYMKPVGDSK-VAMD-EYYS------ELMLGGHNRISIHNVCEDS 430

.| ...|::|:||.| :|:. ||.| |||:.| ||: ||

3cin_A 277 YIKPTGYLEPLGDKKFIAIHIEYVSFNGATDELMING--RIN------DS 318

1p1h_C 431 LLATPLIIDLLVMTEFCTRVSYKKVDK--FENFYPVLTFLSYWLKAPLTR 478

.....|::||: |:....:|: |...|||..|. :..

3cin_A 319 PALGGLLVDLV-------RLGKIALDRKEFGTVYPVNAFY-------MKN 354

1p1h_C 479 PGFHPVNGLNKQRTALENFLRLLIGL-PSQNELRFEERLL 517

|| |....|..|......:|:..|| |..

3cin_A 355 PG--PAEEKNIPRIIAYEKMRIWAGLKPKW 382

Number of Common residues (highlighted in yellow) =23

**6]** Query= 2d37_A(155 letters)

>2d37_A

MAEVikSImrKFPlGVAIVTTNWKGELVGMTVntFNSLSLNPPLVSFfAdRMkGNDIPYKESKYFVVNFTDNEELFNIFALKPVKERFREIKYKEGIGGCPILYDSYAYIEAKLYDTIDVGdhSIIVGEVIDGYQIRDNFTPLVyMNrKYYKLSS

Top Hit= 1rz1_A; Length = 153

>1rz1_A

XDDRLfrnAXgKFATGVTVITTELNGAVHGXTAnaFXsVSlNPKLVLVSIGEKAKXLEKIQQSKKYAVNILSQDQKVLSXNFaGqLEKPVDVQFEELGGLPVIKDALAQISCQVVNEVQAGdhTLFIGEVTDIKITEQDPLLfFSgKYHQLAQ

Score = 74.7 bits (182), Expect = 1e-16

Identities = 50/149 (33%), Positives = 78/149 (52%), Gaps = 13/149 (8%)

Query: 11 KFPLGVAIVTTNWKGELVGMTVNTFNSLSLNPPLVSFFADRMKGNDIPYKESKYFVVNF- 69

KF GV ++TT G + G T N F S+SLNP LV ++SK + VN

Sbjct: 12 KFATGVTVITTELNGAVHGXTANAFXSVSLNPKLVLVSIGEKAKXLEKIQQSKKYAVNIL 71

Query: 70 -TDNEELFNIFA---LKPVKERFREIKYKEGIGGCPILYDSYAYIEAKLYDTIDVGDHSI 125

D + L FA KPV +F E +GG P++ D+ A I ++ + + GDH++

Sbjct: 72 SQDQKVLSXNFAGQLEKPVDVQFEE------LGGLPVIKDALAQISCQVVNEVQAGDHTL 125

Query: 126 IVGEVIDGYQIRDNFTPLVYMNRKYYKLS 154

+GEV D +I + PL++ + KY++L+

Sbjct: 126 FIGEVTD-IKITEQ-DPLLFFSGKYHQLA 152

**actual NIRs in query** (small red) = 14

**NIRs in target** (small green)= 14

**Common NIRs (True positive residues in query which were actually NIRs and also predicted as NIRs in alignment)** (highlighted in yellow) =06

**Global alignment by Needle [EBI-EMBOSS]**

# Length: 162

# Identity: 51/162 (31.5%) # Similarity: 85/162 (52.5%)# Gaps:16/162 ( 9.9%) # Score: 193.0

#=======================================

2d37_A 1 MAEVIKSIMRKFPLGVAIVTTNWKGELVGMTVNTFNSLSLNPPLVSF-F 48

...:.::...||..||.::||...|.:.|.|.|.|.|:||||.||.. .

1rz1_A 1 XDDRLFRNAXGKFATGVTVITTELNGAVHGXTANAFXSVSLNPKLVLVSI 50

2d37_A 49 ADRMKGNDIPYKESKYFVVNF--TDNEELFNIFA---LKPVKERFREIKY 93

.::.|..: ..::||.:.||. .|.:.|...|| .|||..:|.|

1rz1_A 51 GEKAKXLE-KIQQSKKYAVNILSQDQKVLSXNFAGQLEKPVDVQFEE--- 96

2d37_A 94 KEGIGGCPILYDSYAYIEAKLYDTIDVGDHSIIVGEVIDGYQIRDNFTPL 143

:||.|::.|:.|.|..::.:.:..|||::.:|||.| .:|.:. .||

1rz1_A 97 ---LGGLPVIKDALAQISCQVVNEVQAGDHTLFIGEVTD-IKITEQ-DPL 141

2d37_A 144 VYMNRKYYKLSS 155

::.:.||::|:.

1rz1_A 142 LFFSGKYHQLAQ 153

Number of Common residues (highlighted in yellow) =09

**Table S28: Calculation of the rate of false Positive Prediction by the NADbinder server**

In order to demonstrate the rate of false positive prediction, we evaluate our method on a dataset of NAD binding and non-NAD binding proteins. Our dataset contain our original data of NAD binding proteins and 137 non-NAD binding (negative) proteins (non-redundant at 40% CDHIT; data provided in the supplemental file1) which do not bind to any ligands extracted from the Protein Data Bank (PDB). Combined positive and negative data was divided into five sets for 5 fold cross validation. 4 sets were trained on the optimized parameter of the SVM and 5th set was tested. So by this way we test the positive proteins for the sensitivity and result of negative proteins gave the specificity and ultimately accuracy of the prediction.

Increasing the prediction threshold definitely reduces the false positive prediction and increases the specificity but on the other hand sensitivity decreases. The question arises whether we can discriminate NAD and non-NAD binding proteins based on percent of NAD interacting residues (NIRs) prediction. For each protein we calculate the percentage of predicted NIRs over length i.e. **(TP+FP)/length** at threshold 0, 0.1, 0.2 and 0.3. At the threshold of 0.3, we find a balance between sensitivity and specificity where accuracy is achievable up to 72% if used 10% prediction cutoff. In short if any user submits an unknown protein of 100 residues and 10 or more residues are predicted to be NIRs by the server at threshold 0.3 then the accuracy of prediction will be 72% otherwise the prediction could be considered as false positive.

**Definitions:**

Thr = cut off % **(TP + FP)/length** above which Prediction was considered as positive and protein as NAD binding protein otherwise False positive prediction

TP = True Positive, FN=False Negative, TN=True Negative, FP=False Positive, SEN=Sensitivity, SPE=Specificity, ACC= Accuracy

**Prediction at Threshold 0**

Thr TP FN TN FP SEN(%) SPE(%) ACC(%)

5 181 0 1 136 100.00 0.73 57.23

10 180 1 16 121 99.45 11.68 61.64

11 179 2 20 117 98.90 14.60 62.58

12 178 3 29 108 98.34 21.17 65.09

13 175 6 39 98 96.69 28.47 67.30

14 170 11 42 95 93.92 30.66 66.67

15 161 20 47 90 88.95 34.31 65.41

16 142 39 52 85 78.45 37.96 61.01

17 130 51 59 78 71.82 43.07 59.43

18 115 66 66 71 63.54 48.18 56.92

19 88 93 73 64 48.62 53.28 50.63

20 73 108 75 62 40.33 54.74 46.54

21 54 127 81 56 29.83 59.12 42.45

22 38 143 87 50 20.99 63.50 39.31

23 27 154 91 46 14.92 66.42 37.11

24 21 160 97 40 11.60 70.80 37.11

25 10 171 101 36 5.52 73.72 34.91

**Prediction Threshold 0.1**

Thr TP FN TN FP SEN(%) SPE(%) ACC(%)

5 181 0 8 129 100.00 5.84 59.43

10 178 3 44 93 98.34 32.12 69.81

11 171 10 50 87 94.48 36.50 69.50

12 166 15 57 80 91.71 41.61 70.13

13 152 29 62 75 83.98 45.26 67.30

14 131 50 67 70 72.38 48.91 62.26

15 115 66 73 64 63.54 53.28 59.12

16 102 79 82 55 56.35 59.85 57.86

17 78 103 91 46 43.09 66.42 53.14

18 54 127 93 44 29.83 67.88 46.23

19 39 142 101 36 21.55 73.72 44.03

20 27 154 105 32 14.92 76.64 41.51

21 16 165 112 25 8.84 81.75 40.25

22 7 174 115 22 3.87 83.94 38.36

23 6 175 118 19 3.31 86.13 38.99

24 5 176 124 13 2.76 90.51 40.57

25 3 178 125 12 1.66 91.24 40.25

**Prediction Threshold 0.2**

Thr TP FN TN FP SEN(%) SPE(%) ACC(%)

5 180 1 26 111 99.45 18.98 64.78

10 162 19 69 68 89.50 50.36 72.64

11 152 29 77 60 83.98 56.20 72.01

12 133 48 82 55 73.48 59.85 67.61

13 115 66 91 46 63.54 66.42 64.78

14 90 91 96 41 49.72 70.07 58.49

15 67 114 104 33 37.02 75.91 53.77

16 50 131 111 26 27.62 81.02 50.63

17 32 149 117 20 17.68 85.40 46.86

18 19 162 122 15 10.50 89.05 44.34

19 8 173 122 15 4.42 89.05 40.88

20 6 175 125 12 3.31 91.24 41.19

21 3 178 128 9 1.66 93.43 41.19

22 1 180 130 7 0.55 94.89 41.19

23 0 181 131 6 0.00 95.62 41.19

24 0 181 131 6 0.00 95.62 41.19

25 0 181 132 5 0.00 96.35 41.51

**Prediction Threshold 0.3**

Thr TP FN TN FP SEN(%) SPE(%) ACC(%)

5 179 2 49 88 98.90 35.77 71.70

**10 134 47 96 41 74.03 70.07 72.33**

11 118 63 99 38 65.19 72.26 68.24

12 95 86 114 23 52.49 83.21 65.72

13 69 112 120 17 38.12 87.59 59.43

14 48 133 124 13 26.52 90.51 54.09

15 33 148 125 12 18.23 91.24 49.69

16 20 161 129 8 11.05 94.16 46.86

17 11 170 131 6 6.08 95.62 44.65

18 5 176 134 3 2.76 97.81 43.71

19 1 180 136 1 0.55 99.27 43.08

20 0 181 136 1 0.00 99.27 42.77

21 0 181 137 0 0.00 100.00 43.08

22 0 181 137 0 0.00 100.00 43.08

23 0 181 137 0 0.00 100.00 43.08

24 0 181 137 0 0.00 100.00 43.08

25 0 181 137 0 0.00 100.00 43.08
