# Supplementary material for: Identification of NAD interacting residues in proteins
Source: BMC Bioinformatics. 2010 Mar 30;11:160. doi: 10.1186/1471-2105-11-160 (PMC2853471; doi:10.1186/1471-2105-11-160)
Supplement: Additional file 2 — Data used in the development of server. File contains data including protein sequences with NAD interacting residues and PDB IDs with and without redundancy. [file 1471-2105-11-160-S2.DOC]

**SUPPLEMENTARY MATERIAL-**

**Additional File 2**

# Identification of NAD interacting residues in proteins

# Hifzur R Ansari, Gajendra PS Raghava^§^

Institute of Microbial Technology, Sector- 39A, Chandigarh, India-160036.

^§^Corresponding author

Email: HRA - hrahman@imtech.res.in; GPSR - raghava@imtech.res.in

| **List of NAD binding PDB IDs (555) as obtained from SuperSite Database** | | | | | | | | | |
| --- | --- | --- | --- | --- | --- | --- | --- | --- | --- |
| 1A4Z  1A5Z  1A71  1A7A  1A7K  1A9Y  1A9Z  1AD3  1AGN  1AHH  1AHI  1ARZ  1AXE  1AXG  1B14  1B3R  1B8U  1B8V  1BDB  1BI9  1BMD  1BPW  1BTO  1BVR  1BW9  1BXG  1BXK  1BXS  1C14  1C1D  1C1X  1CDO  1CER  1CW3  1CWU  1D1S  1D1T  1D4F  1D7O  1D8A  1DBV  1DC6  1DEH  1DFG  1DFH  1DFI  1DHR  1DHS  1DIR  1DLI  1DO8  1DQS  1DRU  1DSS  1DXY  1E3E | 1E3L  1E3S  1E3W  1E6W  1EBF  1EE2  1EE9  1EFK  1EFL  1EJ2  1EK5  1EMD  1ENO  1ENP  1ENY  1ENZ  1EVJ  1EVZ  1EZ4  1F0Y  1F3P  1F8F  1F8G  1FDV  1FK8  1FMC  1G1A  1G6K  1GAD  1GAE  1GCO  1GD1  1GEE  1GEG  1GEU  1GGA  1GIQ  1GPD  1GR0  1GRB  1GT2  1GUY  1GUZ  1GV0  1GY8  1GYP  1H2H  1H94  1HDG  1HDR  1HDX  1HDY  1HDZ  1HET  1HEU  1HEX | 1HKU  1HL3  1HLD  1HLP  1HSO  1HSZ  1HT0  1HTB  1HWY  1HYH  1HZJ  1HZZ  1I24  1I2B  1I2C  1I2Z  1I30  1I3K  1I3L  1I3M  1I3N  1IB0  1IB6  1ICI  1IE3  1IL0  1IY8  1J0X  1J49  1J5P  1JKF  1JQ5  1JU9  1K0U  1K4M  1K6X  1KAE  1KEP  1KER  1KET  1KEU  1KEW  1KOL  1KQN  1KVQ  1KVR  1KVS  1KVT  1KVU  1KY4  1KYQ  1LA2  1LC3  1LDE  1LDG  1HF3 | 1LDY  1LI4  1LJ8  1LLD  1LLQ  1LLU  1LRJ  1LRK  1LRL  1LSJ  1LSO  1LSS  1LTH  1LVL  1LW7  1LX6  1LXC  1M2W  1M75  1M76  1M8F  1M8G  1M8J  1M8K  1M9H  1MA0  1MC5  1MEW  1MFP  1MG0  1MG5  1MGO  1MI3  1MJT  1ML3  1MP0  1MUU  1MV8  1MX3  1N2S  1NAH  1NAI  1NBO  1NFB  1NFF  1NFR  1NHG  1NHW  1NM5  1NNU  1NPD  1NPT  1NQ5  1NQA  1NQO  1E3I | 1NUU  1NVB  1NVE  1NVM  1NXG  1NZW  1NZX  1NZZ  1O00  1O01  1O02  1O04  1O5I  1O6Z  1O9B  1O9J  1OBB  1OC2  1OC4  1OG3  1OJS  1OJZ  1OMO  1ORR  1OWB  1P1H  1P1I  1P44  1P45  1P9L  1PJ3  1PJC  1PJL  1PJS  1PL6  1PL8  1PSD  1PZH  1QAX  1QAY  1QG6  1QLH  1QR6  1QRR  1QS2  1QSG  1QV6  1QV7  1QXS  1RKX  1RLZ  1ROZ  1RQD  1LDM  1LDN  1NRX | 1RWB  1RZ1  1S20  1S7G  1SB8  1SB9  1SBY  1SC6  1SG6  1SM9  1SOW  1SZJ  1T24  1T2D  1T2F  1T90  1TAE  1TEH  1TOX  1U1I  1U28  1U2D  1U3T  1U3U  1U3V  1U3W  1U5C  1U7H  1U7T  1U8F  1U8X  1UDA  1UDB  1UDC  1UH5  1UP6  1UP7  1UR5  1UWK  1UWL  1UXG  1UXH  1UXI  1UXJ  1UXK  1UXT  1V59  1V8B  1V9L  1VBI  1VC2  1VI2  1VJT  1VKO  1VM6 | 1W1U  1WDK  1WDL  1WDM  1WNB  1WPQ  1WWK  1WXH  1WZE  1X0X  1X14  1X15  1X1T  1X31  1X7D  1X87  1XAG  1XAH  1XAJ  1XAL  1XCB  1XEL  1XLT  1XWF  1Y3I  1Y9E  1YBA  1YC2  1YE4  1YE6  1YL7  1YWG  1Z0Z  1Z2I  1Z45  1Z9A  1ZBQ  1ZEM  1ZJZ  1ZK1  1ZMC  1ZNQ  1ZPT  1ZRQ  1ZSN  1ZW1  1ZXB  1ZXL  2A5F  2A9K  2AG5  2AQ8  2AQH  2AQI  2AQK | 2B37  2B4R  2B4T  2B69  2BHP  2BI4  2BJA  2BJK  2BKJ  2BL4  2BRU  2C20  2C54  2C59  2C5A  2C5E  2C8C  2C8F  2C8H  2CFC  2CNB  2CZC  2D1Y  2D37  2D3T  2D4E  2D4V  2D8A  2DC1  2DFD  2DFV  2DLD  2DPH  2DT5  2DVM  2E37  2ED4  2EER  2EHU  2EII  2EIT  2EJV  2EKL  2EP7  2EWM  2FKN  2FM3  2FN7  2FNZ  2FOI  2FR8  2FRD  2FSV  2FZW  2G5C | 2G8Y  2GAG  2GAH  2GDZ  2GR9  2GRU  2GSD  2GWL  2H4F  2H4H  2H5L  2H7I  2H7L  2H7M  2H7N  2H7P  2HAE  2HDH  2HSD  2HU2  2HUN  2I29  2I2F  2I65  2I9P  2IXA  2IXB  2IZZ  2J40  2J5N  2JHF  2JHG  2LDB  2NAD  2NPX  2NSD  2NSY  2O23  2O2S  2O2Y  2O2Z  2O4C  2OHX  2OL4  2OME  2ONM  2ONP  2OOR  2OOS  2OP0  2OP1  2OXI  2P5U  2P5Y  2PD3 | 2PH5  2PI1  2PLA  2PV7  2PZJ  2PZK  2PZL  2PZM  2Q1T  2Q1U  2Q1W  2Q2Q  2Q2V  2Q3E  2QG4  2QJO  2RAB  2RC3  2UDP  2V7G  2V7P  2VHX  2VOJ  2YVF  2YVG  3B4W  3B6J  3BTO  3BTS  3CEA  3CIN  3DBV  3GPD  3HAD  3HDH  3HUD  3LDH  4MDH  5MDH  6ADH  9LDB  9LDT  1R37  1R66  1R6D  1RFM  2PD4  2PD6  2G76  2G82  2B35  2B36  1VRQ  1VRW  1NR5 |

| **List of NAD binding PDB IDs after 90% sequence redundancy cutoff (CD-HIT)** | | | | | |
| --- | --- | --- | --- | --- | --- |
| 1a5z_A  1a71_A  1a7a_A  1a7k_A  1a9y_A  1ad3_A  1agn_A  1ahi_A  1arz_C  1b14_A  1b8u_A  1bdb_A  1bi9_A  1bmd_A  1bpw_A  1bvr_A  1bw9_A  1bxk_B  1bxs_A  1cdo_A  1d7o_A  1dbv_O  1dc6_A  1deh_A  1dhr_A  1dhs_A  1dli_A  1do8_A  1dss_G  1dxy_A  1e3e_A  1e3w_D  1ebf_A  1ee9_A  1emd_A  1evj_A  1evz_A  1ez4_B  1f3p_A  1f8f_A  1f8g_A  1fdv_A  1fk8_A  1g6k_A  1geg_E  1geu_B | 1h94_A  1hdg_O  1hex_A  1hku_A  1hlp_A  1hwy_A  1hyh_A  1hzz_C  1i24_A  1i3k_A  1ib0_A  1ici_A  1iy8_A  1j49_A  1j5p_A  1jkf_A  1jq5_A  1k4m_A  1k6x_A  1kae_A  1ker_B  1keu_A  1kol_A  1kqn_A  1kyq_B  1lc3_A  1ldm_A  1ldn_A  1lj8_A  1lld_A  1llq_A  1llu_A  1lss_A  1lvl_A  1lw7_A  1m8k_A  1m9h_A  1ma0_A  1mew_A  1mg5_A  1mi3_A  1mjt_B  1ml3_A  1muu_A  1n2s_A  1nbo_A | 1obb_A  1og3_A  1ojs_A  1ojz_A  1omo_A  1orr_A  1p1h_C  1p1i_B  1p9l_A  1pjc_A  1pjs_B  1pl6_A  1pzh_A  1qax_A  1qs2_A  1qsg_G  1r37_A  1r66_A  1rfm_A  1rkx_C  1rz1_A  1s20_G  1s7g_A  1sb8_A  1sc6_D  1sow_B  1t2d_A  1t2f_A  1t90_A  1tae_A  1tox_A  1u1i_A  1u28_B  1u5c_A  1u7h_A  1u7t_A  1u8f_O  1u8x_X  1up6_A  1up6_E  1uwk_A  1uxg_A  1uxt_A  1v59_A  1v8b_A  1gy8_C | 1x15_A  1x1t_A  1x31_A  1x31_C  1x31_D  1x87_A  1xag_A  1xah_A  1y3i_A  1y9e_A  1yba_A  1ywg_O  1z0z_A  1z2i_A  1z45_A  1zbq_A  1zem_A  1zjz_A  1zmc_D  1zrq_B  2a5f_A  2a5f_B  2a9k_A  2ag5_A  2b35_C  2b69_A  2bi4_A  2bkj_A  2bru_C  2c20_A  2c59_A  2c8h_C  2cfc_A  2czc_A  2d1y_C  2d37_A  2d4e_C  2d4v_A  2dc1_A  2dfd_A  2dfv_A  2dld_A  2dph_A  2dt5_B  2dvm_A  1gv0_A | 2g8y_A  2gag_A  2gag_B  2gag_C  2gag_D  2gdz_A  2gr9_A  2gru_A  2gsd_A  2gwl_A  2h4f_A  2hae_A  2hdh_A  2hsd_A  2hun_A  2i2f_A  2i65_B  2i9p_B  2ixa_A  2nad_A  2npx_A  2nsy_A  2o2s_A  2o2y_C  2o2z_A  2o4c_A  2ome_A  2p5u_A  2pd3_A  2pd6_D  2ph5_A  2pi1_A  2pla_A  2pv7_B  2pzm_A  2q1u_A  2q1w_A  2q2q_D  2qg4_B  1nfb_A  1nff_A  1npd_B  1nuu_A  1nvb_B  1nvm_A  1giq_A | 2ewm_B  2fkn_B  2fnz_B  2g5c_B  2g76_A  1wdl_A  1wnb_A  1wwk_A  1wxh_A  1x0x_A  1nvm_B  1nxg_A  1o01_C  1o5i_A  1o9j_A  2qjo_B  2rab_A  2rc3_C  2vhx_E  3b4w_A  3b6j_A  3bts_B  3cea_A  3cin_A  3gpd_G  3ldh_A  4mdh_A  9ldb_A  2e37_C  2ed4_A  2eer_B  2ehu_A  2ejv_A  2ekl_A  2ep7_B  1v9l_A  1vbi_A  1vc2_A  1vjt_A  1vko_A  1vm6_A  1wdk_C  1gr0_A  1grb_A  1guz_A |

| **List of NAD binding PDB IDs and chains after 60% sequence redundancy cutoff (CD-HIT)** | | | | | | |
| --- | --- | --- | --- | --- | --- | --- |
| 1a5z_A  1a71_A  1a7a_A  1a9y_A  1ad3_A  1ahi_A  1arz_C  1b8u_A  1bdb_A  1bpw_A  1bvr_A  1bw9_A  1cdo_A  1dbv_O  1dhr_A  1dhs_A  1dli_A  1do8_A  1dxy_A  1e3e_A  1e3w_D  1ebf_A  1ee9_A  1emd_A  1evj_A  1evz_A  1ez4_B  1f3p_A  1f8f_A  1f8g_A  1fdv_A  1fk8_A  1g6k_A  1geg_E | 2g8y_A  2gag_A  2gag_B  2gdz_A  2gr9_A  2gru_A  2gsd_A  2gwl_A  2h4f_A  2hae_A  2hdh_A  2hsd_A  2hun_A  2i2f_A  2i65_B  1xag_A  1y3i_A  1y9e_A  1yba_A  1ywg_O  1z0z_A  1wdl_A  1wnb_A  1wwk_A  1wxh_A  1x0x_A  1x15_A  1x1t_A  1x31_C  1x31_D  1x87_A  1vjt_A  1vko_A  1vm6_A | 1gr0_A  1grb_A  1guz_A  1gy8_C  1h94_A  1hex_A  1hku_A  1hlp_A  1hwy_A  1hyh_A  1hzz_C  1i24_A  1i3k_A  1ib0_A  1ici_A  1iy8_A  1j5p_A  1jq5_A  1k4m_A  1k6x_A  1kae_A  1ker_B  1keu_A  1kol_A  1kqn_A  1kyq_B  1lc3_A  1ldn_A  1lj8_A  1lld_A  1llq_A  1llu_A  1lss_A  1lvl_A | 1ojs_A  1ojz_A  1omo_A  1orr_A  1p1h_C  1p9l_A  1pjc_A  1pjs_B  1pl6_A  1pzh_A  1qax_A  1qs2_A  1qsg_G  1r37_A  1r66_A  1rfm_A  1rkx_C  1rz1_A  1s20_G  1s7g_A  1sb8_A  1t2d_A  1t2f_A  1t90_A  1tae_A  1tox_A  1u1i_A  1u7h_A  1u8x_X  1up6_A  1uwk_A  1uxg_A  1uxt_A  1geu_B | 1z2i_A  1z45_A  1zbq_A  1zem_A  1zjz_A  1zmc_D  1zrq_B  2a5f_A  2a5f_B  2a9k_A  2ag5_A  2b69_A  2bi4_A  2bkj_A  2bru_C  2c20_A  2c59_A  2c8h_C  2cfc_A  2czc_A  2d1y_C  2d37_A  2d4e_C  2d4v_A  2dc1_A  2dfd_A  2dfv_A  2dld_A  2dph_A  2dt5_B  2dvm_A  2e37_C  1wdk_C  1m8k_A | 2i9p_B  2ixa_A  2npx_A  2nsy_A  2o2s_A  2o2y_C  2o2z_A  2o4c_A  2p5u_A  2pd3_A  2pd6_D  2ph5_A  2pi1_A  2pv7_B  2pzm_A  2q1u_A  2q1w_A  2q2q_D  2qg4_B  2qjo_B  2rab_A  2rc3_C  2vhx_E  3b4w_A  3b6j_A  3bts_B  3cea_A  3cin_A  3ldh_A  4mdh_A  2ep7_B  2ewm_B  1lw7_A  1giq_A | 1m9h_A  1mew_A  1mg5_A  1mi3_A  1mjt_B  1ml3_A  1muu_A  1n2s_A  1nbo_A  1nfb_A  1nff_A  1npd_B  1nuu_A  1nvb_B  1nvm_A  1nvm_B  1nxg_A  1o01_C  1o5i_A  1obb_A  1og3_A  2fnz_B  2fr8_A  2g5c_B  2g76_A  2ed4_A  2ehu_A  2ejv_A  2ekl_A  1v59_A  1v8b_A  1v9l_A  1vbi_A |

| **List of NAD binding PDB IDs and chains after 40% sequence redundancy cutoff (CD-HIT)** | | | | | |
| --- | --- | --- | --- | --- | --- |
| 1a7a_A  1ad3_A  1ahi_A  1arz_C  1bdb_A  1bpw_A  1bvr_A  1bw9_A  1dhr_A  1dhs_A  1dli_A  1dxy_A  1e3e_A  1e3w_D  1ebf_A  1ee9_A  1evj_A  1evz_A  1ez4_B  1f3p_A  1f8f_A  1f8g_A  1fdv_A  1fk8_A  1g6k_A  1geg_E  1giq_A  1gr0_A  1grb_A  1guz_A  1gy8_C  1h94_A  1hex_A | 1lvl_A  1lw7_A  1m8k_A  1m9h_A  1mew_A  1mg5_A  1mi3_A  1mjt_B  1ml3_A  1muu_A  1n2s_A  1nfb_A  1npd_B  1nvb_B  1nvm_A  1nvm_B  1nxg_A  1o5i_A  1o9j_A  1obb_A  1og3_A  1ojs_A  1ojz_A  1omo_A  1orr_A  1p1h_C  1p9l_A  1pjs_B  1pl6_A  1qax_A  1qs2_A  1r37_A  1rfm_A | 1x0x_A  1x14_B  1x31_C  1x31_D  1xag_A  1y3i_A  1y9e_A  1yba_A  1z0z_A  1z2i_A  1z45_A  1zbq_A  1zem_A  1zjz_A  1zrq_B  2a5f_A  2a5f_B  2a9k_A  2ag5_A  2b69_A  2bi4_A  2bkj_A  2c20_A  2c59_A  2c8h_C  2czc_A  2d1y_C  2d37_A  2d4e_C  2d4v_A  2dc1_A  2dfd_A  2dfv_A | 2i65_B  2i9p_B  2ixa_A  2npx_A  2nsy_A  2o2s_A  2o2z_A  2o4c_A  2p5u_A  2pd3_A  2pd6_D  2ph5_A  2pi1_A  2pv7_B  2pzm_A  2q1u_A  2q2q_D  2qg4_B  2qjo_B  2rc3_C  2vhx_E  3b4w_A  3b6j_A  3bts_B  3cea_A  3cin_A  4mdh_A  1wdk_C  1wdl_A  1wnb_A  2hsd_A  2hun_A  2i2f_A | 1hwy_A  1hyh_A  1hzz_C  1i24_A  1ib0_A  1iy8_A  1j5p_A  1jq5_A  1k4m_A  1k6x_A  1kae_A  1keu_A  1kqn_A  1kyq_B  1lc3_A  1lj8_A  1llq_A  1llu_A  1lss_A  2gag_A  2gag_B  2gdz_A  2gr9_A  2gru_A  2gsd_A  2gwl_A  2hdh_A  2dph_A  2dt5_B  2dvm_A  2e37_C  2dld_A  1hlp_A | 1s20_G  1s7g_A  1sb8_A  1t2d_A  1t2f_A  1t90_A  1tae_A  1tox_A  1u1i_A  1u7h_A  1u8x_X  1up6_A  1uwk_A  1uxt_A  1v59_A  1v8b_A  1v9l_A  1vbi_A  1vm6_A  2ed4_A  2ehu_A  2ekl_A  2ewm_B  2fm3_A  2g5c_B  2g76_A  2g8y_A  1rkx_C  1rz1_A  1hku_A |

**NAD binding protein sequences in FASTA format at 40% sequence redundancy WITH NAD INTERACTING RESIDUES LABELLED IN lower case letters.**

>1a7a_A

SDKLPYKVADIGLAAWGRKALDIAENEXPGLXRXRERYSASKPLKGARIAGCLHXTVETAVLIETLVTLGAEVQWSSCNIFSTQNHAAAAIAKAGIPVYAWKGETDEEYLWCIEQTLYFKDGPLNXILDDGGDLTNLIHTKYPQLLPGIRGISEEtttGVHNLYKXXANGILKVPAINVNDSVTkSKFdnLYGcRESLIDGIKRATDVXIAGKVAVVagYgdvGKGCAQALRGFGARVIIteidPInALQAAXEGYEVTTXDEACQEGNIFVTTtgcIDiILGRHFEQXKDDAIVCNighFDVeIDVKWLNENAVEKVNIKPQVDRYRLKNGRRIILLAEGRlVnLGCAXGhPSFVXSNSFTNQVXAQIELWTHPDKYPVGVHFLPKKLDEAVAEAHLGKLNVKLtKlTEKqAQYlGXSCDGPfkPDHyRY

>1ad3_A

SISDTVKRAREAFNSGKTRSLQFRIQQLEALQRMINENLKSISGALASDLGKNEWTSYYEEVAHVLEELDTTIKELPDWAEDEPVAKTRQTQQDDLYIHSEPLGVVLVIGawnYPFNlTIQPMVGAVAAGNAVILkPsevSGHMADLLATLIPQYMDQNLYLVVKGGvPEttELlKERFDHIMytgsTAvGKivMAAAAKHLTPVTLELgGKSPCYVDKDCDLDVACRRIAWGKFMNSGQTcVAPDYILCDPSIQNQIVEKLKKSLKDFYGEDAKQSRDYGRIInDrhFQrVKGLIDNQKVAHGGTWDQSSRYIAPTILVDVDPQSPVMQeeIfGPVMPIVCVRSLEEAIQFINQREKPLALYVFSNNEKVIKKMIAETSSGGVTANDVIVHITVPTLPFGGVGNSGMGAYHGKKSFETFSHRRSCLVKSLLNEEAHKARYPPSPA

>1ahi_A

MFNSDNLRLDGKCAIITgAgagiGKEIAITFATAGASVVVsdinADaANHVVDEIQQLGGQAFACRCditSEQELSALADFAISKLGKVDILVNnaggGGPKPFDMPMADFRrAYElNVFSFFHLSQLVAPEMEKNGGGVILTiTsMAAENKNINMTSyASSkAAASHLVRNMAFDLGEKNIRVNGIApGaiLtdalKSVITPEIEQKMLQHTPIRRLGQPQDIANAALFLCSPAASWVSGQILTVSGGGVQELN

>1arz_C

DANIRVAIAgAGgrmGRQLIQAALALEGVQLGAALerEGSSLLGSDAGELAGAGKTGVTVQSSLDAVKDDFDVFIDftrPegTLnhLAFCRQHGKGMVIgttGFDEAGKQAIRDAAADIAIVFAAnfSVGVNVMLKLLEKAAKVMGDYTDIEIIEAHhRHkVdAPSGTALAMGEAIAHALDKDLKDCAVYSREGHTGERVPGTIGFATVRAGDIVGEHTAMFADIGERLEITHKASSrMTfANGAVRSALWLSGKESGLFDMRDVLDLNNL

>1bdb_A

MKLKGEAVLITgGasglGRALVDRFVAEGAKVAVLdkSAErLAELETDHGDNVLGIVGdvrSLEDQKQAASRCVARFGKIDTLIPnagiWDYSTALVDLPEESLDAAFDEVFHiNVKGyIHAVKACLPALVASRGNVIFtIsnAGFYPNGGGPLyTAAkHAIVGLVRELAFELAPYVRVNGVGsGgiNsdlrGPSSLGPlADMLKSVLPIGRMPEVEEYTGAYVFFATRGDAAPATGALLNYDGGLGVRGFFSGAGGNDLLEQLNIH

>1bpw_A

AQLVDSMPSASTGSVVVTDDLNYWGGRRIKSKDGATTEPVFEPATGRVLCQMVPCGAEEVDQAVQSAQAAYLKWSKMAGIERSRVMLEAARIIRERRDNIAKLEVINNGKTITEAEYDIDAAWQCIEYYAGLAPTLSGQHIQLPGGAFAYTRREPLGVCAGiLawnyPFMiAAWKCAPALACGNAVVFkPspmTPVTGVILAEIFHEAGVPVGLVNVVQGgaETgsLLcHHPNVAKVSftgsVPtGKkvMEmSAKTVKHVTLeLgGKSPLLIFKDCELENAVRGALMANFLTQGQVcTNGTRVFVQREIMPQFLEEVVKRTKAIVVGDPLLTETRMGGLISKPqLDKVLGFVAQAKKEGARVLCGGEPLTPSDPKLKNGYFMSPCVLDNCRDDMTCVKEeIfGPVMSVLPFDTEEEVLQRANNTTFGlASGVFTRDISRAHRVAANLEAGTCYINTYSISPVEVPfGGYKMSGFGReNGQATVDYYSQLKTVIVEMGDVDSL

>1bvr_A

TGLLDGKRILVSgIiTDSsiAFHIARVAQEQGAQLVLTGfDRLRLiQRITDRLPAKAPLLEldvqNEEHLASLAGRVTEAIGAGNKLDGVVHsigfMPQTGMGINPFFDAPYADVSKGIHiSAYSYASMAKALLPIMNPGGSIVGmDfDPSRAMPAyNWmTVAkSALESVNRFVAREAGKYGVRSNLVAaGpiRtLAmSAIVGGALGEEAGAQIQLLEEGWDQRAPIGWNMKDATPVAKTVCALLSDWLPATTGDIIYADGGAHTQLL

>1bw9_A

SIDSALNWDGEMTVTRFDSMTGAHFVIRLDSTQLGPAAGGTRAAQYSNLADALTDAGKLAGAMTLkMAVSNLPMGGGkSVIALPAPRHSIDPSTWARILRIHAENIDKLSGNYWTGpdvNTNSADMDTLNDTTEFVFGRSLERGGAGSSAFTtAVGVFEAMKATVAHRGLGSLDGLTVLVqgLgavGGSLASLAAEAGAQLLVadtdTErVAHAVALGHTAVAlEDvLSTPCDVFAPcamGGVITTEVARTLDCSVVAGaanNVIADEAASDILHARGILYAPDFVAnAGGAIHLVGREVLGWSESVVHERAVAIGDTLNQVFEISDNDGVTPDEAARTLAGRRAREAST

>1dhr_A

EARRVLVygGrgalGSRCVQAFRARNWWVASIdvvENEEASASVIvKMTDSFTEqADQVTAEVGKLLGDQKVDAILCvaggwAGGNAKSKSLFKNCDLMWkqSIWtSTISSHLATKHLKEGGLLTLaGaKAALDGTPGMIGyGMAkGAVHQLCQSLAGKNSGMPSGAAAIAVLpVtlDtPMnRKSMPEADFSSWTPLEFLVETFHDWITGNKRPNSGSLIQVVTTDGKTELTPAYF

>1dhs_A

APAGALAAVLKHSSTLPPESTQVRGYDFNRGVNYRALLEAFGTTGFQATNFGRAVQQVNAMIEKKLEPLTSCTIFLGYtsnlIsSGIRETIRYLVQHNMVDVLVTtAgGVeeDLIKCLAPTYLGEFSLRGKELRENGINRiGNLLVPNENyCKFEDWLMPILDQMVMEQNTEGVKWTPSKMIARLGKEINNPESVYYWAQKNHIPVFSPaLTdgSLGDMIFFHSYKNPGLVLDIVEDLRLINTQAIFAKCTGMIILgggvVKHHIANANLMRNGADYAVYIntaQEFDGSDsGARPDEAVSWGKIRVDAQPVKVYAdasLVFPLLVAETFAQKMDAFMHEKNED

>1dli_A

MKIAVAgSgyvGLSLGVLLSLQNEVTIvdilPSkVDKINNGLSPiQDEYIEYYLKSKQLSIKATLDsKAAyKEAELVIIatptNYNSRINYFDTQhVEtvIKeVLSVNSHATLIIKstIPIGFITEMRQKFQTDRIIFSPeFlReSKALYDNLYPSRIIVSCEENDSPKVKADAEKFALLLKSAAKKNNVPVLIMGASEAEAVkLFANTYLALRVAYFNELDTYAESRKLNSHMIIQGISYDDRIGMHYNNPSFGYGGycLPkDTKQLLANYNNIPQTLIEAIVSSNNVRKSYIAKQIINVLKEQESPVKVVGVYRLIMKSNSDNFrESAIKDVIDILKSKDIKIIIYEPMLNKLESEDQSVLVNDLENFKKQANIIVTNRYDNELQDVKNKVYSRDIFGRD

>1dxy_A

MKIIAYGARVDEIQYFKQWAKDTGNTLEYHTEFLDENTVEWAKGFDGINSLQTTPYAAGVFEKMHAYGIKFLTIRNvGTDNIDMTAMKQYGIRLSNVPAySPAAiAEFALTDTLYLLRNMGKVQAQLQAGDYEKAGTFIGKELGQQTVGVmgtghiGQVAIKLFKGFGAKVIAydpyPMKGDHPDFDYVSLEDLFKQSDVIDLhvpGiEqnTHiINEAAFNLMKPGAIVINTarPNLIDTQAMLSNLKSGKLAGVGIdtYEYETEDLLNLAKHGSFKDPLWDELLGMPNVVLSPhIayYTETAVHNMVYFSLQHLVDFLTKGETSTEVTG

>1e3e_A

GTQGKVIKCKAAIAWKTGSPLCIEEIEVSPPKACEVRIQVIATCVcptDINATDPKKKALFPVVLGHECAGIVESVGPGVTNFKPGDKVIPFfAPQCKRCKLCLSPLTNLCGKLRNFKYPTIDQELMEDRTSRFTCKGRSIYHFMGVSSFSQYTVVSEANLARVDDEANLERVCLIGcgFSsGYGAAINTAKVTPGSTCAVfgLgcvGLSAIIGCKIAGASRIIAidinGEkFPKAKALGATDCLNpRELDKPVQDVITELTAGGVDYSLDCaGtAqtLKAAVDCTVLGWGSCTVvgAkVDEMTIPTVDViLGRSINGtffGGWKSVDSVPNLVSDYKNKKFDLDLLVTHALPFESINDAIDLmKEGKSirTILTF

>1e3w_D

SVKGLVAVITgGasglGLSTAKRLVGQGATAVLldvPNsEGETEAKKLGGNCIFAPAnvtSEKEVQAALTLAKEKFGRIDVAVNcagiAVAIKTYHEKKNQVHTLEDFQRVINvNLIGTFNVIRLVAGVMGQNEPDQGGQRGVIINtAsVAAFEGQVGQAAySASkGGIVGMTLPIARDLAPIGIRVVTIApGlfAtpllTTLPDKVRNFLASQVPFPSRLGDPAEYAHLVQMVIENPFLNGEVIRLDGAIRMQP

>1ebf_A

STKVVNVAVigAgvvGSAFLDQLLAMKSTITYNLVLLaeaeRSLISKDFSPLNVGSDWKAALAASTTKtLPlDDLIAHLKTSPKPVILVDNtssAyiAGFYTKFVENGISIATpnkKAFSSDLATWKALFSNKPTNGFVYHEATVGaGLPIISFLREIIQTGDEVEKIEGIFSGTLSYIFNEFSTSQANDVKFSDVVKVAKKLGYTePDPRDDLNGLDVARkVTIVGRISGVEVESPTSFPVQSLIPKPLESVKSADEFLEKLSDYDKDLTQLKKEAATENKVLRFIGKVDVATKSVSVGIEKYDYSHPFASLKGSDNVISIKTKRYTNPVVIQGAGagAAVtAAGVLGDVIKIAQRL

>1ee9_A

KPGRTILASKVAETFNTEIINNVEEYKKTHNGQGPLLVGFLANNDPAAKMyATWTQKTSESMGFRYDLRVIEDKDFLEEAIIQANGDDSVNGIMVYFpVFGNAQDQYLQQVVCKEKDVEGLNHVYYQNLYHNVRYLDKENRLKSILPCtPLAIVKILEFLKIYNNLLPEGNRLYGKKCIVinRsEivGRPLAALLANDGATVYSvdvNNIQKFTRGESLKLNKHHVEDLGEySEDLlKKCSLDSDVVITGvpSENyKFPTEYIKEGAVCINfactKNFSDDVKEKASLYVPmtgKVtIAMLLRNMLRLVRNVELSKE

>1evj_A

RRFGYAIVgLgkyALNQILPGFAGCQHSRIEALVdgNAEKAKIVAAEYGVDPRKIYDySNFDKIAKDPKIDAVYIilpnSlhAEFAIRAFKAGKHVMCekpMATSVADCQRMIDAAKAANKKLMIGYrCHYDPMNRAAVKLIRENQLGKLGMVTTDNSDVMDQNDPAQqwrLRRElAGGGSLMdIGIyGLNGTRYLLGEEPIEVRAYTYSDPNDERFVEVEDRIIWQMRFRSGALSHGASSYSTTTTSRFSVQGDKAVLLMDPATGyYQNLISVQTPMPANNQFSAQLDHLAEAVINNKPVRSPGEEGMQDVRLIQAIYEAARTGRPVNTDWGYVRQGGY

>1evz_A

KDELLYLNKAVVFgsgafGTALAMVLSKKCREVCVwhmNEEEVRLVNEKRENVLfLKGVQLASNITFTSDVEKAYNGAEIILFvipTQfLRGFFEKSGGNLIAYAKEKQVPVLVCtkGIERSTLKFPAEIIGEFLPSPLLSVLAGPsfaIEVATGVFTCVSIASADINVARRLQRIMSTGDRSFVCWATTDTVGCEVASAVknVLAIGSGVANGLGMGLNARAALIMRGLLEIRDLTAALGGDGSAVFGLAGLGDLQLTCSSELSRNFTVGKKLGKGLPIEEIQRAVAeGVATADPLMRLAKQLKVKMPLCHQIYEIVYKKKNPRDALADLLSCGLQDEGLPPLFK

>1ez4_B

SMPNHQKVVLvgDgavGSSYAFAMAQQGIAEEFVIVdvvKDrTKGDALDLEDAQAFTAPKKIYSGEySDCKDADLVVItagapQKPGESRLDLVNKNLNiLSsiVKPVVDSGFDGIFLVaanPvDILTYATWKFSGFPKERVIGsGTSlDSSrLRVALGKQFNVDPRSVDAYIMGEhGDSEFAAYSTATIGTRPVRDVAKEQGVSDDDLAKLEDGVRNKAYDIINLKGAtfYGiGTALMRISKAILRDENAVLPVGAYMDGQYGLNDIYIGTPAIIGGTGLKQIIESPLSADELKKMQDSAATLKKVLNDGLAELENK

>1f3p_A

ALKAPVVVLGAGLASVSFVAELRQAGYQGLITVVGDEAERPYDRPPLSKDFMAHGDAEKIRLDCKRAPEVEWLLGVTAQSFDPQAHTVALSDGRTLPYGTLVLATGAAPrAlPTLQGATMPVHTLrTLEDARRIQAGLRPQSRLLIvgGgviGLeLAATARTAGVHVSLvetqPRLmsrAAPATLADFVARYHAAQGVDLRFERsVTGSVDGVVLLDDGTRIAADMVVVgigvLANDALARAAGLACDDGIFVDAYGRTTCPDVYALGdVTRQRNPLSGRFERIetWSNAQNQGIAVARHLVDPTAPGYAELPWyWsDQGALRIQVAGLASGDEEIVRGEVSLDAPKFTLIELQKGRIVGATCVNNARDFAPLRRLLAVGAKPDRAALADPATDLRKLAAA

>1f8f_A

LKDIIAAVTPCKGADFELQALKIRQPQGDEVLVKVVATGMchtDLiVRDQKYPVPLPAVLGHEGSGIIEAIGPNVTELQVGDHVVLSYGYCGKCTQCNTGNPAYCSEFFGRNFSGADSEGNHALCVNDHFFAQSSFATYALSRENNTVKVTKDVPIELLGPLGcgIQtGAGACINALKVTPASSFVTwgAgavGLSALLAAKVCGASIIIAvdivESrLELAKQLGATHVINsKTQDPVAAIKEITDGGVNFALEstGsPeiLKQGVDALGILGKIAVvgaPQLGTTAQFDVNDLLLGGKTILGVveGSGSPKKFIPELVRLYQQGKFPFDQLVKFYAFDEINQAAIDsRKGITLkPIIKIA

>1f8g_A

XKIAIPKERRPGEDRVAISPEVVKKLVGLGFEVIVEQGAGVGASITDDALTAAGATIASTAAQALSQADVVWKVQRPXTAEEGTDEVALIKEGAVLXCHLGALTNRPVVEALTKRKITAYAXELXPrISRAqSXdIlsSQsNLAGYRAVIDGAYEFARAFPXXXTAAGTVPPARVLVFgVgvaGlQAIATAKRLGAVVXAtdvrAATKEqVESLGGKFITVDDEATAeTAGgyaKEXGEEfRKKqAEAVLKELVKTDIAITtalipGKPApVlITEEXVTKXKPGSVIIDLAVEAGGNCPLSEPGKIVVKHGVKIVGHTNVPSRVAADASPLFAKNLLNFLTPHVDKDTKTLVXKLEDETVSGTCVTRDGAIVHPALTGQGA

>1fdv_A

ARTVVLItgCssgiGLHLAVRLASDPSQSFKVYATLrDLKTQGRLWEAARALACPPGSLETLQLdvrDSKSVAAARERVTEGRVDVLVCnaglGLLGPLEALGEDAVASVLDvNVVGTVRMLQAFLPDMKRRGSGRVLVtGsVGGLMGLPFNDVyCASkFALEGLCESLAVLLLPFGVHLSLIEcGpvHtAfMEkVLGSPEEVLDRTDIHTFHRFYQYLALSKQVfREAAQNPEEVAEVFLTALRAPKPTLRYFTTERFLPLLRMRLDDPSGSNYVTAMHREVFG

>1fk8_A

MSIIVISgCatgiGAATRKVLEAAGHQIVGidirDAEVIAdlsTAEGRKQAIADVLAKCSKGMDGLVLcaglgPQTKVLGNVVSvNYFGATELMDAFLPALKKGHQPAAVViSsvASAHLAFDKNPLALALEAGEEAKARAIVEHAGEQGGNLAyAGSkNALTVAVRKRAAAWGEAGVRLNTIApGatEtPFVPPMGRRAEPSEMASVIAFLMSPAASYVHGAQIVIDGGIDAVMRPTQF

>1g6k_A

MYKDLEGKVVVITgSstglGKSMAIRFATEKAKVVVnYrSKEDeANSVLEEIKKVGGEAIAVKGdvtVESDVINLVQSAIKEFGKLDVMINnaglANPVSSHEMSLSDWNKVIDtNLTGAFLGSREAIKYFVENDIKGTVINmSsvHEKIPWPLFVHyAASkGGMKLMTETLALEYAPKGIRVNNIGpGaiNtpinAEKfADPEQRADVESMIPMGYIGEPEEIAAVAAWLASSEASYVTGITLFADGGMTQYPSFQAGRG

>1geg_E

MKKVALVTgAgqgiGKAIALRLVKDGFAVAIAdynDAtAKAVASEINQAGGHAVAVKvdvSDRDQVFAAVEQARKTLGGFDVIVNnagvAPSTPIESITPEIVDKVYNiNVKGVIWGIQAAVEAFKKEGHGGKIINaCsQAGHVGNPELAVySSSkFAVRGLTQTAARDLAPLGITVNGyCpGivKtpmwAEIDRQVSEAAGKPLGYGTAEFAKRITLGRLSEPEDVAACVSYLASPDSDYMTGQSLLIDGGMVFN

>1giq_A

IERPEDFLKDKENAIQWEKKEAERVEKNLDTLEKEALELYKKDSEQISNYSQTRQYFYDYQIESNPREKEYKNLRNAISKNKIDKPINVYYFESPEKFAFNKEIRTENQNEISLEKFNELKETIQDKLFKQDGFKDVSLYEPGNGDEKPTPLLIHLKLPKNTGMLPYINSNDVKTLIEQDYSIKIDKIVRIVIEGKQYIKAEASIVNSLDFKDDVSKGDLWGKENYSDWSNKLTPNELADVNDYMRGGyTAInNYLiSNGPLNNPNPELDSKVNNIENALKLTPIPSNLIVYrrSgPqeFGLTLTSPEYDFNKIENIDAFKEKWEGKVITypnFIstsIGSVNmSAfAKrKIILRINIPKDSPGAYLSAIPGyAGeYeVLLNHGSKFKINKVDSYKDGTVTKLILDATLIN

>1gr0_A

TEVRVAIvgVgncASSLVQGVEYYYNADDTSTVPGLMHVRFGPYHVRDVKFVAAFdvdAKkVGFDLSDAIFASEnNTIKIADVAPTNVIVQRGPtLDgiGKYyADTiELSDAEPVDVVQALKEAKVDVLVSylpvGSEEaDKFyAQCAIDAGVAFVNalpVFIASDPVWAKKFTDARVPIVGDdiKsQVGATITHRVLAKLFEDRGVQLDRTMQLNVGGnmdFLNmLEDVHIGPSDHVGWLDDRKWAYVRLEGRAFGDVPLNLEYKLEVWdsPNsAGVIIDAVRAAKIAKDRGIGGPVIPASAYLMkSPPEQLPDDIARAQLEEFIIG

>1grb_A

VASYDYLVIGGGSGGLASARRAAELGARAAVVESHKLGGTCVNVGCVPKKVMWNTAVHSEFMHDHADYGFPSCEGKFNWRVIKEKRDAYVSRLNAIYQNNLTKSHIEIIRGHAAFTSDPKPTIEVSGKKYTAPHILIATGGMPSTpHESqiPGASLGITSDGFFQLEELPGRSVIvgAgyiAVeMAGILSALGSKTSLMIrHDKVLRSFDSMISTNCTEELENAGVEVLKFSqVKEVKKTLSGLEVSMVTAVPGRLPVMTMIPDVDCLLwAigrVPNTKDLSLNKLGIQTDDKGHIIVDEFQNTNVKGIYAVGdVCGKAllTPVAIAAGRKLAHRLFEYKEDSKLDYNNIPTVVfSHPPIGTVGLTEDEAIHKYGIENVKTYSTSFTPMYHAVTKRKTKCVMKMVCANKEEKVVGIHMQGLGCDEMLQGFAVAVKMGATKADFDNTVAIHPTSSEELVTLR

>1guz_A

MKITVigagnvGATTAFRLAEKQLARELVLldvvEGiPQGKALDMYESGPVGLFDTKVTGSNDyADTANSDIVIItaglpRKPGMTREDLLMKnAGiVKevTDNIMKHSKNPIIIVvsnPlDIMTHVAWVRSGLPKERVIGmaGVlDAArFRSFIAMELGVSMQDINACVLGGhGDAMVPVVKYTTVAGIPISDLLPAETIDKLVERTRNGGAEIVEHLKQGsaFYApASSVVEMVESIVLDRKRVLPCAVGLEGQYGIDKTFVGVPVKLGRNGVEQIYEINLDQADLDLLQKSAKIVDENCKML

>1gy8_C

HMRVLVCgGAgyiGSHFVRALLRDTNHSVVIVdsLvgTHGKSDHVETRENVARKLQQSDGPKPPWADRYAALEVGdvrNEDFLNGVFTRHGPIDAVVHMcAflAVGESVRDPLKYYDnNVVGILRLLQAMLLHKCDKIIFsSsAAIFGNPTMNAEPIDINAKKSPESPyGESkLIAERMIRDCAEAYGIKGICLRyFnaCGAHEDGDIGeHYQGSThLIPIILGRVMSDIAPDASTDKRMPIFGTDYPTPDGTCVRDYVHVCDLASAHILALDYVEKLGPNDKSKYFSVFNLGTSRGYSVREVIEVARKTTGHPIPVRECGRREGDPAYLVAASDKAREVLGWKPKYDTLEAIMETSWKFQRTHPNGYA

>1h94_A

VSEIKTLVTFFgGtgdlAKRKLYPSVFNLYKKGYLQKHFAIVGTarqALNDDEFKQLVRDSIKDFTDDQAQAEAFIEHFSYRAHdvtDAASYAVLKEAIEEAADKFDIDGNRIFYMsvapRffGTIAKYLKSEGLLADTGYNRLMIekpFGTSYDTAAELQNDLENAFDDNQLFRIDHYLGKEMVQNIAALRFGNPIFDAAWNKDYIKNVQVTLCEVLGVEErAGyYDTAGALLdMIQNHTMQIVGWLAMEKPESFTDKDIRAAKNAAFNALKIYDEAEVNKYFVRAQYGAGDSADFKPYLEELDVPADSKNNTFIAGELQFDLPRWEGVPFYVRSGKRLAAKQTRVDIVFKAGTFNFGSEQEAQEAVLSIIIDPKGAIELKLNAKSVEDAFNTRTIDLGWTVSDEDKKNTPEPyERMIHDTMNGDGSNFADWNGVSIAWKFVDAISAVYTADKAPLETYKSGSMGPEASDKLLAANGDAWVFKG

>1hex_A

MKVAVLPGDGiGPEvTEAALKVLRALDEAEGLGLAYEVFPFGGAAIDAFGEPFPEPTRKGVEEAEAVLLGSVggpKWdGLPRKIrPetGLLSLRKSQDLFANLRPAKVFPGLERLSPLKEEIARGVDVLIVRELTGGIYFGEPRGMSEAEAWNTERYSKPEVERVARVAFEAARKRRKHVVSVDKANVLEVGEFWRKTVEEVGRGYPDVALEHQYVDAMAMHLVRSPARFDVVVTGNIFGDILSDLASVLPGSlgLLPSASLGRGTPVFEPVhgsapdiAGKGianPTAAILSAAMMLEHAFGLVELARKVEDAVAKALLETPPPdLGGSAGTEAFTATVLRHLA

>1hku_A

PRPLVALLDGRDXTVEMPILKDVATVAFCDAQSTQEIHEKVLNEAVGALMYHTITLTREDLEKFKALRIIVRIGsgFdNIDIKSAGDLGIAVCNVPAAsvEEtADSTLCHILNLYRRTTWLHQALREGTRVQSVEQIREVASGAARIRGETLGIigLgrvGQAVALRAKAFGFNVLFydpylSDGIERALGLQRVSTLQDLLFHSDCVTLhcgLnEHnHHlINDFTVKQMRQGAFLVNTarGGLVDEKALAQALKEGRIRGAALdvHESEPFSFSQGPLKDAPNLICTPhAawYSEQASIEMREEAAREIRRAITGRIPDSLKNCVNKDHL

>1hlp_A

TKVSVvgAgtvGAAAGYNIALRDIADEVVFVdikEDDTVGQAADTNHGIAYDSNTRVRQGGyEDTAGSDVVVITagipRQPGQTRIDlAGDNAPiMEdiQSSLDEHNDDYISLTtsnpvDLLNRHLYEAGDRSREQVIGfgGRlDSARFRYVLSEEFDAPVQNVEGTILGEhGDAQVPVFSKVRVDGTDPEFSGDEKEQLLGDLQESAMDVIERKGatEWGpARGVAHMVEAILHDTGEVLPASVKLEGEFGHEDTAFGVPVRLGSNGVEEIVEWDLDDYEQDLMADAAEKLSDQYDKIS

>1hwy_A

ADREDDPNFFKMVEGFFDRGASIVEDKLVEDLKTRQTQEQKRNRVRGILRIIKPCNHVLSLSFPIRRDDGSWEVIEGYRAQHSHqrtpCKGGIrYSTDVSVDEVKALASLMTYKCaVVdVpFGGAKAGVKINPkNYTDEDLEKITRRFTMELAKKGFIGPGVDVPApnmsTGEREMSWIADTYASTIGHYDINAhACVTGKPisqgGIhGrISAtGRGVFHGIENFIENASYMSILGMTPGFGDKTFAVqgFgnvGLHSMRYLHRFGAKCVAVGesDGSIWNPDGIDPKELEDFkLQHGTILGFPKAKIYEGSILEVDCDILIPAaseKqLTKSNAPRVKAKIIAEGanGPTTPQADKIFLERNIMVIPDLYLnAGgVTVSyFQILknLNhvsYGrLTFKYERDSNYHLLMSVQESLERKFGKHGGTIPIVPTAEFQDRISGASeKDIVHSGLAYTMErSARqIMRTAMKYNLGLDLRTAAYVNAIEkvFrvYNEAGVTFT

>1hyh_A

ARKIGIigLgnvGAAVAHGLIAQGVADDYVFidanEAkVKADQIDFQDAMANLEAHGNIVINDwAALADADVVISTlgniKLqQFAeLKftSSmVQsvGTNLKESGFHGVLVVisnPvDVITALFQHVTGFPAHKVIGtGTLlDTARMQRAVGEAFDLDPRSVSGYNLGEhGNSQFVAWSTVRVMGQPIVTLIDLAAIEEEARKGGFTVLNGKGytSYGvATSAIRIAKAVMADAHAELVVSNRRDDMGMYLSYPAIIGRDGVLAETTLDLTTDEQEKLLQSRDYIQQRFDEIVDTL

>1i24_A

SRVMVigGdgycGWATALHLSKKNYEVCIVdnlvrRLfDHQLGLESLTPIASIHDRISRWKALTGKSIELYVGdicDFEFLAESFKSFEPDSVVHfgeqrSAPYSMIDRSRAVyTQHnNVIGTLNVLFAIKEFGEECHLVKlGTMGEYGTPNIDIEEGYITITHNGRTDTLPYPKQASSFyHLSkVHDSHNIAFTCKAWGIRATDLNqGvvYGVKTDETEMHEELRNrLDYDAVfGtALNRFCVQAAVGHPLTVYGKGGQTRGYLDIRDTVQCVEIAIANPAKAGEFRVFNQFTEQFSVNELASLVTKAGSKLGLDVKKMTVPNPRVEAEEHYYNAKHTKLMELGLEPHYLSDSLLDSLLNFAVQFKDRVDTKQIMPSVSWKKIGVKTKSM

>1ib0_A

HHHMITLENPDIKYPLRLIDKEILSHDTRRFRFALPSPQHILGLPIGQHIYLSTRIDGNLVIRPYTPVSSDDDKGFVDLVVkVyFKETHPKFPAGGKMSQYLENMNIGDTIEFRGPNGLLVYQGKGKFAIRADKKSNPVVRTVKSVGMIAggtgITPMLQVIRAVLKDPNDHTVCYLLFanqSEKdILLRPELEELRNEHSSRFKLWYtVdKAPDAWDYSQGfvNEEMIRDHLPPPGEETLILMcgpppmiQfaCLPNLERVGHPKERCFtf

>1iy8_A

RFTDRVVLITgGGsglGRATAVRLAAEGAKLSLVdvsSEGLEASKAAVLETAPDAEVLTTVAdvSDEAQVEAYVTATTERFGRIDGFFNnagiEGKQNPTESFTAAEFDKVVSiNLRGVFLGLEKVLKIMREQGSGMVVNtAsvGGIRGIGNQSGyAAAkHGVVGLTRNSAVEYGRYGIRINAIApgAiWtpmvENSMKQLDPENPRKAAEEFIQVNPSKRYGEAPEIAAVVAFLLSDDASYVNATVVPIDGGQSAAY

>1j5p_A

HMTVLIigMgniGKKLVELGNFEKIYAydriSkDIPGVVRLDEFQVPSDVSTVVEcaspeaVKeySLQILKNPVNYIIistSAFADEVFRERFFSELKNSPARVFFPSgaIGGLDVLSSIKDFVKNVRIETIKPPKSLGLDLKGKTVVFEGSVEEASKLFPRNInvAStIGLIVGFEKVKVTIVADPAMDHNIHIVRISSAIGNYEFKIENIsMLtVYSILRTLRNLESKIIFG

>1jq5_A

AAERVFISPAKYVQGKNVITKIANYLEGIGNKTVVIAdEivWKiAGHTIVNELKKGNIAAEEVVFSGEaSRNEVERIANIARKAEAAIVIGVGggktLdTAKAVADELDAYIVIVPtaAstdaPTsAlsvIySDDGVFESYRFYKKNPDLVLVDTKiIANapPRlLAsGIADALATWVEARSVIKSGGKTMAGGIPTIAAEAIAEKCEQTLFKYGKLAYESVKAKVVTPALEAVVEANTLLSGLGfESGGLAAAHAIhNGFTALEGEIHHLthGEKVAFGTLVQLALEEHSQQEIERYIELYLCLDLPVTLEDIKLKDASREDILKVAKAATAEGETIHNAFNVTADDVADAIFAADQYAKAYKEK

>1k4m_A

MKSLQALfggtfDPVhYghLKpVETLANLIGLTRVTIIPnNVpphrPQPEANSVQRKHMLELAIADKPLFTLDEReLKRNAPSytAQTLKEWRQEQGPDVPLAfIigQdsLLtFPtwyEYETILDNAHLIVcRrPGYPLEMAQPQYQQWLEDHLTHNPEDLHLQPAGKIYLAETPWfNIsATIIRERLQNGESCEDLLPEPVLTYINQQGLYR

>1k6x_A

QQKKTIAVVnAtgrqaASLIRVAAAVGHHVRAQVhSLKGLIAEELQAIPNVTLFQGPLLNNVPLMDTLFEGAHLAFInTtSqaGDeIAIGKDLADAAKRAGTIQHYIYsSmPDHSLYGPWPAVPmwAPkFTVENYVRQLGLPSTFVYaGiyNNnFTSLPYPLFQMELMPDGTFEWHAPFDPDIPLPWLDAEHDVGPALLQIFKDGPQKWNGHRIALTFETLSPVQVCAAFSRALNRRVTYVQVPKVEIKVNIPVGYREqLEAIEVVFGEHKAPyFPLPEFSGGVISQRVTDEARKLWSGWRDMEEYAREVFPIEEEANGLDWML

>1kae_A

XSFNTIIDWNSCTAEQQRQLLXRPAISASESITRtVNDiLDNVKARGDEALREYSAKfdKTTVTALKVSAEEIAAASERLSDELKQAXAVAVKNIETFHTAQKLPPVDVETQPGVRCQQVTRPVASVGLyIpGGSAPlFStVLXLATPASIAGCKKVVLCSpPPIADEILYAAQLCGVQDVFNVggAqAIAALAFGTESVPKVDKIFGpgnAfvTEAKRQVSQRLDGAAIDXPAGpsEVLVIADSGATPDFVASDLLSQAEhgPDSQVILLTPAADXARRVAEAVERQLAELPRAETARQALNASRLIVTKDLAQCVEISNQYGPEHLIIQTRNARELVDSITSAGSVFLGDWSPESAGDYASGTNhvLPTYGYTATCSSLGLADFQKRXTVQELSKEGFSALASTIETLAAAERLTAHKNAVTLRVNALKEQA

>1keu_A

MKILITgGAgfiGSAVVRHIIKNTQDTVVNIdkLtyagNLESLSDISESNRYNFEHAdicDSAEITRIFEQYQPDAVMHlaaesHVDRSITGPAAFIEtNIVGTYALLEVARKYWSALGEDKKNNFRFHHiStDEVYGDLPHPDEVENSVTLPLFTETTAYAPSSPySASkASSDHLVRAWRRTYGLPTIVTNcSnnYGPYhfPekLIPLVILNALEGKPLPIYGKGDQIRDWLYVEDHARALHMVVTEGKAGETYNIGGHNEKKNLDVVFTICDLLDEIVPKATSYREQITYVADRPGHDRRYAIDAGKISRELGWKPLETFESGIRKTVEWYLANTQWVNNVKSGAYQSWIEQNYEGRQ

>1kqn_A

KTEVVLLacgsfNPITNmhLRlFELAKDYMNGTGRYTVVKGIISPvGDAyKkKGLIPAYHRVIMAELATKNSKWVEVDTWeSLQKEwKetLKVLRHHQEKLEAAVPKVKLLcgAdlLEsFAVPNlwKSEdITQIVANYGLICVtrAGNDAQKFIYESDVLWKHRSNIHVVNeWIANdIssTKIRRALRRGQSIRYLVPDLVQEYIEKHNLYSSESEDRNAGVILApLQRnTA

>1kyq_B

XVKSLQLAHQLKDKRILLigGgevGLTRLYKLXPTGCKLTLVspdlHKSIIPKFGKFIQNKDQPDYREDAKRFINPNWDPTKNEIYEYIRSdfKDEYLDLENENDAWYIIXTcipdHPeSARIYHLCKERFGKQQLVNVadkPDLCDFYFGANLEIGDRLQILISTNGLSPRFGALVRDEIRNLFTQXGDLALEDAVVKLGELRRGIRLLAPDDKDVKYRXDWARRCTDLFGIQHCHNIDVKRLLDLFKVXFQEQNCSLQFPPRERLLSEYCS

>1lc3_A

miTNSGKFGVVVVGVgraGSVRLRDLKDPRSAAFLNLIGFVSRRELGSLDEVrQISLEDALRsQeIDVAYICSesSShEDYIRQFLQAGKHVLVeypMTLSFAAAQELWELAAQKGRVLHEeHvELLMEEFEFLRREVLGKELLKGSLRfTASPlEEErfGFPAFsGISrLTWLVSLFGELSLISATLEERKEDQYMKMTVQLETQNKGLLSWIEEkGPGLKrNRYVNFQFTSGSLEEVPSVGVNKNIfLKDQDIFVQKLLDQVSAEDLAAEKKRIMHCLGLASDIQKLCHQ

>1lj8_A

XKLNKQNLTQLAPEVKLPAYTLADTRQGIAHigVggfHRAHQAYYTDALXNTGEGLDWSICGVGlrSedRKARDDLAGQDYLFTLYELGDTDDTEVRVIGSISDXLLAEDSAQALIDKLASPEIRIVSLtiteGgYCIDDSNGEFXAHLPQIQHDLAHPSSPKtvFGFICAALTQRRAAGIPAFTVXSCdnLPHNGAVTRKALLAFAALHNAELHDWIKAHVSFPNAXvdRitPXTSTAHRLQLHDEHGIDDAwPVVCEPFVQWVLEDKFVNGRPAWEKVGVQFTDDVTPYEEXkIGLLnGSHLALTYLGFLKGYRFVHETXNDPLFVAYXRAYXDLDVTPNLAPVPGIDLTDYKQTLVDRFSNQAIADQLErVCSDGSSkFPKFTVPTINRLIADGRETERAALVVAAWALYLKGVDENGVSYTIPDPRAEFCQGLVSDDALISQRLLAVEEIFGTAIPNSPEFVAAFERCYGSLRDNGVTTTLKHLLKKP

>1llq_A

SVAHHEDVYSHNLPPMDEKEMALYKLYRPERVTPKKRSAELLKEPRLNKGMGFSLYERQYLGLHGLLPPAFMTQEQQAYRVITKLREQPNDLARYIQLDGLQDRNEKLFYRVVCDHVKELMPIVYTPTVGLACQNFGYIYRKPKGLYITINDNSVSKIYQILSNWHEEDVRAIVVTDGErIlGLGDLGAYGIGIPVGKLALYVALGGVQPKWCLPVLLDVGTNNMDLLNDPFYIGLRHKRVRGKDYDTLLDNFMKACTKKYGQKTLIQFEDFAnPNAFRLLDKYQDKYTMFNDdIQGtASviVAGLLTCTRVTKKLVSQEKYLFfgagaaSTGIAEMIVHQMQNEGISKEEACNRIYLMdiDGLVTKNRKEMNPRhVQFAKDMPETTSILEVIRAARPGALIGastvRGAFNEEVIRAMAEINERPIIFAlsnPTSkAECTAEEAYTFTNGAALYASgSPFPNFELNGHTYKPGQgnnAYIFPGVALGTILFQIRHVDNDLFLLAAKKVASCVTEDSLKVGRVYPQLKEIREISIQIAVEMAKYCYKNGTANLYPQPEDLEKYVRAQVYNTEYEELINATYDWPEQDMRHGFPVPVVRH

>1llu_A

TLPQTMKAAVVHAYGAPLRIEEVKVPLPGPGQVLVKIEASGVchtDLhAAEGDwPVKPPLPFIPGHEGVGYVAAVGSGVTRVKEGDRVGIPWLYTACGCCEHCLTGWETLCESQQNTGYSVNGGYAEYVLADPNYVGILPKNVEFAEIAPILcAGVtVYKGLKQTNARPGQWVAIsgIgglGHVAVQYARAMGLHVAAidiDDAkLELARKLGASLTVNarQEDPVEAIQRDIGGAHGVLVtavsNsaFGqAIGMARRGGTIALvglPPGDFPTPIFDVvLKGLHIAGSivGTRADLQEALDFAGEGLVKATIHPGKLDDINQILDQmRAgQiEGrIVLEM

>1lss_A

MYIIIAgIgrvGYTLAKSLSEKGHDIVLIdidKDiCKKASAEIDALVINGdctkIKTLEDAGIEDADMYIAVtgkEevNLMSSLLAKSYGINKTIArIseIEYKDVFERLGVDVVVSPELIAANYIEKLIER

>1lvl_A

QQTIQTTLLIIGGGPGGYVAAIRAGQLGIPTVLVEGQALGGTALNIGAIPSKALIHVAEQFHQASRFTEPSPLGISVASPRLDIGQSVAWKDGIVDRLTTGVAALLKKHGVKVVHGWAKVLDGKQVEVDGQRIQCEHLLLATGSSSVElPMLPLGGPVISSTEALAPKALPQHLVVvgGgyiGLELGIAYRKLGAQVSVVearERILptYDSELTAPVAESLKKLGIALHLGHsvEGYENGCLLANDGKGGQLRLEADRVLVavgrRpRTKGFNLECLDLKMNGAAIAIDERCQTSMHNVWAIGdVAgEpmlAHRAMAQGEMVAEIIAGKARRFEPAAIAAVCFTDPEVVVVGKTPEQASQQGLDCIVAQFPFAANGRAMSLESKSGFVRVVARRDNHLILGWQAVGVAVSELSTAFAQSLEMGACLEDVAGTIHAHPTLGEAVQEAALRALGHALHI

>1lw7_A

EKKVGVifgkfYPVhTghINXIYEAFSKVDELHVIVcSDTVrDLKLFYDSKXKRXPTVQDRLRWXQQIFKYQKNQIFIHHLVeDGiPSyPNgwQSwSEAVKTLFHEKHFEPSIVFssePQdKAPyEKYLGLEVSLvdPDrTFfNVsaTKIRTTPFQYWKFIPKEARPFFAKTVAILGGESSGKSVLVNKLAAVFNTtsawEyGREFVFEKLGGDEQAMQYSDYPQXALGHQRyIDyaVRHSHKIAFIDTDFITTQAFCIQYEGKAHPFLDSXIKEYPFDVTILLKNNTEQKQRQQFQQLLKKLLDKYKVPYIEIESPSYLDRYNQVKAVIEKVLNEEEISELQN

>1m8k_A

TMRGLlvgrMQPFhRGALQvIKSILEEVDELIICIgsAQLSHSIrDPFTAGERVMMLTKALSENGIPASRYYIIPVQdiECnALwVGHIKMLTPPFDRVYsGnPlvQRlFSEDGYEVTApPLfyRdRYsGTEVRRRMLDDGDWRSLLPESVVEVIDEINGVERIKHLAK

>1m9h_A

TVPSIVLNDGNSIPQLGYgVykVPPADTQRAVEEALEVGYRHIdTAAIyGNEEGVGAAIAASGIARDDLFITTkLWNDRHDGDEPAAAIAESLAKLALDQVDLYLVhwPTPAADNYVHAWEKMIELRAAGLTRSIGVsnHLVPHLERIVAATGVVPAVNqIELHPAYQQREITDWAAAHDVKIESwgplGqgKYDLfGAEPVTAAAAAHGKTPaQAVLRWHLQKGFVVfpgsVRREhLEenLDVFDFDLTDTEIAAIDAMDPGDGSGRVSGHPDEVD

>1mew_A

AKYYNEPCHTFNEYLLIPGLSTVDAIPSNVNLSTPLVKFQKGQQSEINLKIPLVSAIMQSVSGEKMAIALAREGGISFIFGSQSIESQAAMVHAVKNFKAHNELVDSQKRYLVGAGIntrDFRERVPALVEAGADVLCIdssDGFSEwQKITIGWIREKYGDKVKVGAGnIVDGEGFRYLADAGADFIkIGigGGSREQKGiGRGQATAVIDVVAERNKYFEETGIYIPVCSDGGIVYDYHMTLALAMGADFIMLGRYFARFEESPTRKVTINGSVMKEYWGegSSRArNWeGVDSYVPYAGKLKDNVEASLNKVKSTMANCGALTIPQLQSKAKITLVSSVSI

>1mg5_A

SFTLTNKNVIFvaGLggiGLDTSKELLKRDLKNLVILdriENPAAIAELKAINPKVTVTFYPYdvtVPIAETTKLLKTIFAQLKTVDVLINGagiLDDHQIErTIAvNYTGLVNTTTAILDFWDKRKGGPGGIICNiGsVTGFNAIYQVPVySGTkAAVVNFTSSLAKLAPITGVTAYTVNpGitRttlvHKFNSWLDVEPQVAEKLLAHPTQPSLACAENFVKAIELNQNGAIWKLDLGTLEAIQWTKHWDSGI

>1mi3_A

SIPDIKLSSGHLMPSIGFgCwkLANATAGEQVYQAIKAGYRLFdGAEDyGNEKEVGDGVKRAIDEGLVKREEIFLTSkLWNNYHDPKNVETALNKTLADLKVDYVDLFLIhfPIAFKFVPIEEKYPPGFYCGDGNNFVYEDVPILETWKALEKLVAAGKIKSIGVsnFPGALLLDLLRGATIKPAVLqVEHHPYLQQPKLIEFAQKAGVTITAyssfGPqsfVeMNQGRALNTPTLfAHDTIKAIAAKYNKtPaeVLLRWAAQRGIAVipksnLPErLVqnRSFNTFDLTKEDFEEIAKLDIGLRFnDPWDWDNIPIFV

>1mjt_B

HHLFKEAQAFIENMYKECHYETQIINKRLHDIELEIKETGTYTHTEEELIYGAKMAWRNSNRCIGRLFWDSLNVIDARDVTDEASFLSSITYHITQATNEGKLKPYITIYAPKDGPKIFNNQLIRYAGYDNCGDPAEKEVTRLANHLGWKGKGTNFDVLPLIYQLPNESVKFYEYPTSLIKEVPIEHNHYPKLRKLNLKWYAVPIISNMDLKIGGIVYPTAPFNGWYMVTEIGVrNFIDDYRYNLLEKVADAFEFDTLKNNSFNKDRALVELNYAVYHSFKKEGVSIVDHLTAAKQFELFERNEAQQGRQVTGkwswLAPPLSPTLTSNyhhgYDNTVKDPNFFYKK

>1muu_A

XRISIfgLgyvGAVCAGCLSARGHEVIGvdvsSTkIDLINQGKSPIVEPGLEALLQQGRQTGRLSGTTDFKKAVLDSDVSFIcvgtPSKKNGDLDLGyIEtvCReIGFAIREKSERHTVVVRstVLPGTVNNVVIPLIEDCSGKKAGVDFGVGTNPeFLReSTAIKDYDFPPXTVIGELDKQTGDLLEEIYRELDAPIIRKTVEVAEXIKYTCNVWHAAKVTFANEIGNIAKAVGVDGREVXDVICQDHKLNLSRYYXRPGFAFGGscLPkdVRALTYRASQLDVEHPXLGSLXRSNSNQVQKAFDLITSHDTRKVGLLGLSFKAGTDDLrESPLVELAEXLIGKGYELRIFDRNVEYARVHGANKEYIESKIPHVSSLLVSDLDEVVASSDVLVLGNGDELFVDLVNKTPSGKKLVDLVGFXPHTTTAQAEGICW

>1n2s_A

MNILLFgKtgqvGWELQRSLAPVGNLIALdvHSKEFCGdfsNPKGVAETVRKLRPDVIVNaaahtAVDKAESEPElAQLlNATSVEAIAKAANETGAWVVhyStDyVFPGTGDIPWQETDATSPLNVyGKTkLAGEKALQDNCPKHLIFRtswvYAGKGNNFAKTMLRLAKERQTLSVINDQYGAPTGAELLADCTAHAIRVALNKPEVAGLYHLVAGGTTTWHDYAALVFDEARKAGITLALTELNAVPTSAYPTPASRPGNSRLNTEKFQRNFDLILPQWELGVKRMLTEMFTTTT

>1nfb_A

TSYVPDDGLTAQQLFNCGDGLTYNDFLILPGYIDFTADQVDLTSALTKKITLKTPLVSSPMDTVTEAGMAIAMALTGGIGFIHhNCTPEFQANEVRKVKKYEQGFITDPVVLSPGIPITDTGRMGSAPAGITLKEANEILQRSKKGKLPIVNEDDEVAIIANRDYPLASKDAKKQLLCGAAIgtheDDKYRLDLLAQAGVDVVVLdssqGNsIfQINMIKYIKDKYPNLQVIGGnVVTAAQAKNLIDAGVDALrVGmGSGSIcitQeVLACGRPQATAVYKVSEYARRFGVPVIADGGIQNVGHIAKALALGASTVMMGSLLAATTEAPGEYFFSDGIRLKKYRGmDKGSIHKFVPYLIAGIQHSCQDIGAKSLTQVRAMMYSGELKFEKRTSSAQ

>1npd_B

XDVTAKYELIGLXAYPIRHSLSPEXQNKALEKAGLPFTYXAFEVDNDSFPGAIEGLKALKXRGTGVSXPNkQLACEYVDELTPAAKLVGAINTIVNDDGYLRGYNTdGTGHIRAIKESGFDIKGKTXVLLGAggaSTAIGAQGAIEGLKEIKLFnrRdEfFDKALAFAQRVNENTDCVVTVTDlADQQAFAEALASADILTNGtkvGXKPlENeSlVNDISLLHPGLLVTEcvynPHXTKLLQQAQQAGCKTIDGYGXlLWqGAEQFTLWTGKDFPLEYVKQVXGFGA

>1nvm_B

MNQKLKVAIigsgniGTDLMIKVLRNAKYLEMGAMvgidAAsDGLARAQRMGVTTTYAgvEGLIKLPEFADIDFVFDatsASaHVQnEALLRQAKPGIRLIDLtpAAIGPYCVPVVNLEEHLGKLNVNMVtcGGQATIPMVAAVSRVAKVHYAEIVASiSSKSagpgtRAnIDEfTETTSKAIEVIGGAAKGKAIIIMNPAEPPLIMRDTVYVLSAAADQAAVAASVAEMVQAVQAYVPGYRLKQQVQFDVIPESAPLNIPGLGRFSGLKTSVFLEVEGAAHYLPAYAGnlDImTSAALATAERMAQSMLNA

>1nxg_A

ADTKAKLTLNGDTAVELDVLKGTLGQDVIDIRTLGSKGVFTFDPGFTSTASCESKITFIDGDEGILLHRGFPIDQLATDSNYLEVCYILLNGEKPTQEQYDEFKTtVTrhtmIhEQITRLFHAFRRDSHPMAVMCGITGALAAFyHdSLDVNNPRHReiAAfrLLSkMPTMAAMCYKYSIGQPFVYpRnDLSYAGNFLNMMFSTPCEPYEVNPILERAMDRILILHADHEQNASTSTVRTAGSSGANPFACIAAGIASLWGPAHGGANEAALKMLEEISSVKHIPEFFRRAKDKNDSFRLMGFGHRVYKNYDPRATVMRETCHEVLKELGTKDDLLEVAMELENIALNDPYFIEKKLYPNVDFYSGIILKAMGIPSSMFTVIAAMARTVGWIAHWSEMHSDGMKIARPRQLYTGYEKRDFKSDIKR

>1o9j_A

DLPAPLTNIKIQHTKLFINNEWHESVSGKTFPVFNPATEEKICEVEEADKEDVDKAVKAAREAFQMGSPWRTMDASERGQLIYKLADLIERDRLLLATLESINAGKVFASAYLMDLDYCIKALRYCAGWADKIQGRTIPVDGEFFSYTRHEPIGVCGLiFpwnAPMIlLACKIGPALCCGNTVIVkPaeqTPLTALHVASLIKEAGFPPGVVNIVPGYgpTAgaAIsSHMDVDKVAftgsTEvGKmiQEAAAKSNLKRVTLeLgAKNPCIVFADADLDSAVEFAHQGVFTNQGQScIAASKLFVEEAIYDEFVQRSVERAKKYVFGNPLTPGVNHGPQINKAqHNKIMELIESGKKEGAKLECGGGPWGNKGYFIQPTVFSNVTDDMRIAKEeIfGPVQQIMKFKSLDEVIKRANNTYYGlVAGVFTKDLDKAVTVSSALQAGTVWVNCYLAASAQSPAGGFKMSGHGReMGEYGIHEYTEVKTVTMKISEKNS

>1obb_A

PSVKIGIigAgsAVfSLRLVSDLCKTPGLSGSTVTLmdidEErLDAILTIAKKYVEEVGADLKFEKTMNLDDVIIDADFVINtamvGghTYLEKVRQIGEKYGYYRGIDAQEFNMVSdYYTFSNYNqLKyFVDIARKIEKLSPKAWYLQaanPiFEGTTLVTRTVPIKAVGFXhGHYGVMEIVEKLGLEEEKVDWQVAGVNHGIWLNRFRYNGGNAYPLLDKWIEEKSKDWKPENPFNDQLSPAAIDMYRFYGVMPIGDTVRNSSWRYHRDLETKKKWYGEPWGGADSEIGWKWyQDTlGKVTEITKKVAKFIKENPSVRLSDLGSVLGKDLSEKQFVLEVEKILDPERKSGEQHIPFIDALLNDNKARFVVNIPNKGIIHGIDDDVVVEVPALVDKNGIHPEKIEPPLPDRVVKYYLRPRIMRMEMALEAFLTGDIRIIKELLYRDPRTKSDEQVEKVIEEILALPENEEMRKHYLK

>1og3_A

PlMLDTAPNAFDDQYEGAVNKMEEKAPLLLQEDFNMNAKLKVAWEEAKKRWNNIKPSRSYPKGFNDFHGTALVAytGSIavDFnRAVreFKENPGQFHYKAFHYYLTRALQLLSNGDBHSVYrGtKtRfHYTGAGSVRFGqFtsSsLSKKvaQSQEfFSDHGTLFIIKTBLGVYIKEFsFrPDqEiVLIPGYEVYQKVRTQGYNEIFLDSPKRKKSNYNALYS

>1ojs_A

MKLGFvgAgrvGSTSAFTCLLNLDVDEIALVdiaEDlAVGEAMDLAHAAIDKYPKIVGGySLLKGSEIIVVtaglaRKPGMTRLDLAHKNAGiIKdiAKKIVENASKILVvtnPmDVMTYIMWKESGKPRNEVFGmGNQlDSQRLKERLYNAGARNRAWIIGEhGDSMFVAKSLADFDGEVDWEAVENDVRFVAAEVIKRKGatiFGpAVAIYRMVKAVVEDTGEIIPTSMILQGEYGIENVAVGVPAKLGKNGAEVADIKLSDEEIEKLRNSAKILRERLEELGY

>1ojz_A

AETKNFTDLVEATKWGNSLIKSAKYSSKDKMAIYNytKNsSPInTPLrSANGDVNKLSENIQEQVRQLDSTISKSVTPDSVYVYrllnLdyLssITGFTREDLHMLQQTNNGQYNEALVSKLNNLMNSRIYReNGYSsTqLVSGAalAGrPIELKLELPKGTKAAYIDSKELtAyPGqQeVLLPRGTEYAVGSVKLSDNKRKIIITAVVFKK

>1omo_A

METLILTQEEVESLISMDEAMNAVEEAFRLYALGKAQMPPKVYLEFEKGDLRAMPAHLMGYAGLKWVNSHPGNPDKGLPTvMALMILNSPETGFPLAVMDATYTtsLrtGAAGGIAAKYLARKNSSVFGFigCgtqAYFQLEALRRVFDIGEVKAydvrEKaAKKFVSYCEDRGISASVQPAEEASRCDVLVTttpsRkPvVKAEWVEEGTHINAigAdGPGkQELDVEILKKAKIVVDDLEQAKHGGEINVAVSKGVIGVEDVHATIGEVIAGLKDGRESDEEITIFDstgLAIQDVAVAKVVYENALSKNVGSKIKFF

>1orr_A

AKLLITgGCgflGSNLASFALSQGIDLIVfdnlsrKGaTDNLHWLSSLGNFEFVHGdirNKNDVTRLITKYMPDSCFHlagQvAMTTSIDNPCMDFEiNVGGTLNLLEAVRQYNSNCNIIysStNKVYGDLEQYKYNETETRYTCVDKPNGYDESTQLDFHSPyGCSkGAADQYMLDYARIFGLNTVVFRhSsmYGGRqFATYDqgWVGWFCQKAVEIKNGINKPFTISGNGKQVRDVLHAEDMISLYFTALANVSKIRGNAFNIGGTIVNSLSLLELFKLLEDYCNIDMRFTNLPVRESDQRVFVADIKKITNAIDWSPKVSAKDGVQKMYDWTSSI

>1p1h_C

TSVKVVTDKCTYKDNELLTKYSYENAVVTKTASGRFDVTPTVQDYVFKLDLKKPEKLGIMLigLGgnnGSTLVASVLANKHNVEFQTKEGVKQPNYFGSMTQCSTLKLGIDAEGNDVYAPFNSLLPMVSPNDFVVSGWdiNNADLYEAMQrSQVLEYDLQQRLKAKMSLVKPLPsIyYPDFiAANqDErANNCINLDEKGNVTTRGKWTHLQRIRRDIQNFKEENALDKVIVLwtanteRYVEVSPGVNDTMENLLQSIKNDHEEIApSTIfAAASILEGVPYINGspQNTFVPGLVQLAEHEGTFIAGDDlKsGQTKLKSVLAQFLVDAGIKPVSIASYNHLGnndGYnLSAPKQFRSkEISKSSVIDDIIASNDILYNDKLGKKVDHCIVIKYMKPVGdSKVAMDEYYSELMLGGHNRISIHNVCEdsLLaTPLIIDLLVMTEFCTRVSYKKVDKFENFYPVLTFLSYWLkAPLTRPGFHPVNGLNKQRTALENFLRLLIGLPSQNELRFEERLL

>1p9l_A

MRVGVlgAkgkvGTTMVRAVAAADDLTLSAEldaGDPlSLLTDGNTEVVIDfthpDvVMGnLEFLIDNGIHAVVgttGFTAERFQQVESWLVAKPNTSVLIAPnfAIGAVLSMHFAKQAARFFDSAEVIELHhPHkAdAPSGTAARTAKLIAEARKGLPPNPDATSTSLPGARGADVDGIPVHAVRLAGLVAHQEVLFGTEGETLTIRHDSLDrTSfVPGVLLAVRRIAERPGLTVGLEPLLDLH

>1pjs_B

MDHLPIFCQLRDRDCLIvgGgdvAErKARLLLEAGARLTVNaltFiPQFTVWANEGMLTLVEGpFDETLLDSCWLAIAAtddDTvNQrVSDAAESRRIFCNVvdAPKAASFIMPSIIDRSPLMVAVSXGGTSPVLARLLREKLESLLPQHLGQVARYAGQLRARVKKQFATMGERRRFWEKFFVNDRLAQSLANADEKAVNATTERLFSEPLDHRGEVVLVGAGPGDAGLLTLKGLQQIQQADIVVYDRLVSDDIMNLVRRDADRVFVGKRAGYHCVPQEEINQILLREAQKGKRVVRLKGGDPFIFGRGGEELETLCHAGIPFSVVPGITAASGCSAYSGIPLTHRDYAQSVRLVTGHGGELDWENLAAEKQTLVFYMGLNQAATIQEKLIAFGMQADMPVALVENGTSVKQRVVHGVLTQLGELAQQVESPALIIVGRVVALRDKLNWFSNH

>1pl6_A

AAAAKPNNLSLVVHGPGDLRLENYPIPEPGPNEVLLRXHSVGIcgSDVHYWEYGRIGNFIVKKPXVLGHEASGTVEKVGSSVKHLKPGDRVAIEPGAPRENDEFCKXGRYNLSPSIFFCATPPDDGNLCRFYKHNAAFCYKLPDNVTFEEGALIEPLSvGIHACRRGGVTLGHKVLVCgAgpiGXVTLLVAKAXGAAQVVVtdlsATrLSKAKEIGADLVLQiSKESPQEIARKVEGQLGCKPEVTIECtGaEasIQAGIYATRSGGTLVLvglGSEXTTVPLLHAAIREVDIKGvfrYCNTWPVAISXLASKSVNVKPLVTHRFPLEKALEAFETfKKGLGLKIXLKCDPSDQNP

>1qax_A

DSRLPAFRNLSPAARLDHIGQLLGLSHDDVSLLANAGALPMDIANGMIENVIGTFELPYAVASNFQINGRDVLVPLVVeepSIVAAASYMAKLARANGGFTTSSSAPLMHAQVQIVGIQDPLNARLSLLRRKDEIIELANRKDQLLNSLGGGCRDIEVHTFADTPRGPMLVAHLIVDVRDAMGANTVNTMAEAVAPLMEAITGGQVRLRILSNLADLRLARAQVRITPQQLETAEFSGEAVIEGILDAYAFAAVDPYRAAtHNkGIMNGIDPLIVATGNDWRAVEAGAHAYAARSGHYGSLTTWEKDNNGHLVGTLEMPMPVGLVGGATKTHPLAQLSLRILGVKTAQALAEIAVAVGLAQNLGAMRALATEGiqRGhMALhARNiAVvAGARGDEVDWVARQLVEYHDVraDRAVALLKQKRGQ

>1qs2_A

KVEDFKEDKEKAKEWGKEKEKEWKLTATEKGKMNNFLDNKNDIKTNYKEITFSMAGSFEDEIKDLKEIDKMFDKTNLSNSIITYKNVEPTTIGFNKSLTEGNTINSDAMAQFKEQFLDRDIKFDSYLDTHLTAQQVSSKERVILKVTVPSGKGSTTPTKAGVILNNSEYKMLIDNGYMVHVDKVSKVVKKGVECLQIEGTLKKSLDFKNDINAEAHSWGMKNYEEWAKDLTDSQREALDGyaRQDyKEInNYLrnQGGSGNEKLDAQIKNISDALGKKPIPENITVYrwCgMpeFGYQISDPLPSLKDFEEQFLNTIkEdKGYmstsLSSERLAAfGSrKIILRLQVPKGSTGAYLSAIGGFAsEKeILLDKDSKYHIDKVTEVIIKGVKRYVVDATLLTN

>1r37_A

MRAVRLVEIGKPLSLQEIGVPKPKGPQVLIKVEAAGVchsDVhMRQGRFGNLRIVEDLGVKLPVTLGHEIAGKIEEVGDEVVGYSKGDLVAVNPWQGEGNCYYCRIGEEHLCDSPRWLGINFDGAYAEYVIVPHYKYMYKLRRLNAVEAAPLTcSGItTYRAVRKASLDPTKTLLVvgAGgglGTMAVQIAKAVSGATIIGvdvrEEAVEAAKRAGADYVINaSMQDPLAEIRRITESKGVDAVIDlnNsEktLSVyPKALAKQGKYVMvglfGADLHYHAPLItLSEIQFVGSlvGNQSDFLGIMRLAEAGKVKPMITKTMKLEEANEAIDNlENfKAIGrQVLIP

>1rfm_A

XILKPENEKKLIIDVLKKFGVPEEDAKITADVFVDADLKGfTShGIGRFPQYITALKLGNINPKPDIKIVKESPATAVIDGDLGLGQVVGKKAXELAIKKAKNVGVGVVATRNANhfGiaGYYSELAXNQDXIGITItNtEPAXAPfGGKEKILGtNpIAIAFKGNKYKFSldXaTASIaRGKILEALRKKIKIPEGCAVDKDGKPTTDPAKALEGCILPFGGpkGyGLALAIEXLSAIGGAEVGTKVKGtANPEERCTKGDLFIAINPEFFXGKEEFKRKVDELLDEIKNSEPAEGFEIlIpGEieERNKXKRKDGFEIDKNLYNQLKEICNELGLNIEDYIE

>1rkx_C

INNSFWQGKRVFVTgHtgfkGGWLSLWLQTMGATVKGYslTApTVPSLFETARVADGMQSEIGdirDQNKLLESIREFQPEIVFHmaaqpLVRLSYSEPVETYStNVMGTVYLLEAIRHVGGVKAVVNiTsdkCYDNKEWIWGYRENEAMGGYDPySNSkGCAELVTSSYRNSFFNPANYGQHGTAVATVRAgnvIGGGdWALDrIVPDILRAFEQSQPVIIRNPHAIRPWQHVLEPLSGYLLLAQKLYTDGAEYAEGWNFGPNDADATPVKNIVEQMVKYWGEGASWQLPHEAHYLKLDCSKAKMQLGWHPRWNLNTTLEYIVGWHKNWLSGTDMHEYSITEINNYMNTK

>1rz1_A

XDDRLfrnAXgKFATGVTVITTELNGAVHGXTAnaFXsVSlNPKLVLVSIGEKAKXLEKIQQSKKYAVNILSQDQKVLSXNFaGqLEKPVDVQFEELGGLPVIKDALAQISCQVVNEVQAGdhTLFIGEVTDIKITEQDPLLfFSgKYHQLAQ

>1s20_G

XKVTFEQLKAAFNRVLISRGVDSETADACAEXFARTTESGVYSHGVNRFPRFIQQLENGDIIPDAQPKRITSLGAIEQWDAQRSIGNLTAKKXXDRAIELAADHGIGLVALRNANHWXRGGSYGWQAAEKGYIGICWTNSIAVXPPWGAKECRIGTNPLIVAIPSTPITXVDXSXSXFSYGXLEVNRLAGRQLPVDGGFDDEGNLTKEPGVIEKNRRILPXGYWKGSGXSIVLDXIATLLSDGASVAEVTQDNSDEYGISQIFIAIEVDKLIDGPTRDAKLQRIXDYVTSAERADENQAIRLPGHEFTTLLAENRRNGITVDDSVWAKIQALLEH

>1s7g_A

MEDEIRKAAEILAKSKHAVVFTgagISaeSGIPtfRGeDGLwRKYDPEEVASISGFKRNPRAFWEFSMEMKDKLFAEPNPAHYAIAELERMGIVKAVITqnIDMLHQRAGSRRVLELhGSMDKLDCLDCHETYDWSEFVEDFNKGEIPRCRKCGSYYVKPRVVLfGEPLPQRTLFEAIEEAKHCDAFMVVgssLvvYPaAELPYIAKKAGAKMIIVnaePtMADPIFDVKIIGkaGEVLPKIVEEVKRLRSE

>1sb8_A

MMSRYEELRKELPAQPKVWLITgVAgfiGSNLLETLLKLDQKVVGldnFatgHQRNLDEVRSLVSEKQWSNFKFIQGdirNLDDCNNACAGVDYVLhqaalgSVPRSINDPITSNAtNIDGFLNMLIAARDAKVQSFTYaAsSSTYGDHPGLPKVEDTIGKPLSPyAVTkYVNELYADVFSRCYGFSTIGLRyFnvFGRRqdPNGAYAAVIPKWTSSMIQGDDVYINGDGETSRDFCYIENTVQANLLAATAGLDARNQVYNIAVGGRTSLNQLFFALRDGLAENGVSYHREPVYRDFREGDVRHSLADISKAAKLLGYAPKYDVSAGVALAMPWYIMFLK

>1t2d_A

APKAKIVLvgSgmiGGVMATLIVQKNLGDVVLfdivKNmPHGKALDTSHTNVMAYSNCKVSGSNTyDDLAGADVVIVtagftKAPGKSDKEwNRDDlLPLnNKiMIeiGGHIKKNCPNAFIIVvtnPvDVMVQLLHQHSGVPKNKIIGlgGVlDTSRLKYYISQKLNVCPRDVNAHIVGAhGNKMVLLKRYITVGGIPLQEFINNKLISDAELEAIFDRTVNTALEIVNLHAsPyVApAAAIIEMAESYLKDLKKVLICSTLLEGQYGHSDIFGGTPVVLGANGVEQVIELQLNSEEKAKFDEAIAETKRMKALA

>1t2f_A

ATLKEKLIAPVAEEEATVPNNKITVvgvgqvGMACAISILGKSLADELALVdvlEDkLKGEMMDLQHGSLFLQTPKIVADKDySVTANSKIVVVtagvrqQEGESRLNlVQRnVNvFKfiIPQIVKYSPDCIIIVvsnPvDILTYVTWKLSGLPKHRVIGsGCNlDSARFRYLMAEKLGIHPSSCHGWILGEhGDSSVAVWSGVNVAGVSLQELNPEMGTDNDSENWKEVHKMVVESAYEVIKLKGytNWAiGLSVADLIESMLKNLSRIHPVSTMVKGMYGIENEVFLSLPCILNARGLTSVINQKLKDDEVAQLKKSADTLWDIQKDLKF

>1t90_A

EIRKLKNYINGEWVESKTDQYEDVVNPATKEVLCQVPISTKEDIDYAAQTAAEAFKTWSKVAVPRRARILFNFQQLLSQHKEELAHLITIENGKNTKEALGEVGRGIENVEFAAGAPSLMMGDSLASIATDVEAANYRYPIGVVGGiApfnfPMMvPCwMFPMAIALGNTFILkPserTPLLTEKLVELFEKAGLPKGVFNVVYGahDVvnGILEHPEIKAISfvgsKPvGEyVYKKGSENLKRVQSlTgAKNHTIVLNDANLEDTVTNIVGAAFGSAGERcMACAVVTVEEGIADEFMAKLQEKVADIKIGNGLDDGVFLGPVIREDnKKRTLSYIEKGLEEGARLVCDGRENVSDDGYFVGPTIFDNVTTEMTIWKDeIfAPVLSVIRVKNLKEAIEIANKSEFAnGACLFTSNSNAIRYFRENIDAGMLGINLGVPAPMAFFPfSGWKSSFFGTLHANGKDSVDFYTRKKVVTARYPAPDF

>1tae_A

QPLTLTAATTRAQELRKQLNQysHEyyVKDQpSvEdYVydRLYKELVDIETEFPDLITPDSPTQRVGGKVLSGFEKAPHDIPMYslNdGFSKEDIFAFDERVRKAIGKPVAYCCeLkiDGLaISLRYENGVFVRGATrGDGTVGENITENLRTVRSVPMRLTEPISVEVRGeCYMPKQSFVALNEEREENGQDIFANPRNAAAGSLRQLDTKIVAKRNLNTFLytVADFGPMKAKTQFEALEELSAIGFRTNPERQLCQSIDEVWAYIEEYHEKRSTLPYEIdGIvIkVNEFALQDELGFTVKAPRWAIAYkFPPEEAETV

>1tox_A

GADDVVDSSKSFVMENFSSYhGtkPGyVDsiQKGiqKDDDWKGfyStDNKYDaAGySVDNENPLSGKAGGVVKVTYPGLTKVLALKVDNAETIKKELGLSLTEPLMEQVGTEEFIKRFGDGASRVVLSLPfAEGSSSVeYINNwEQAKALSVELEINFETRGKRGQDAMYEYMAQAASAINLDWDVIRDKTKTKIESLKEHGPIKNKMSESPNKTVSEEKAKQYLEEFHQTALEHPELSELKTVTGTNPVFAGANYAAWAVNVAQVIDSETADNLEKTTAALSILPGIGSVMGIADGAVHHNTEEIVAQSIALSSLMVAQAIPLVGELVDIGFAAYNFVESIINLFQVVHNSYNRPAYSPGHKTQPFLHDGYAVSWNTVEDSIIRTGFQGESGHDIKITAENTPLPIAGVLLPTIPGKLDVNKSKTHISVNGRKIRMRBRAIDGDVTFBRPKSPVYVGNGVHANLHVAFHRSSSEKIHSNEISSDSIGVLGYQKTVDHTKVNSKLSLFFEIKS

>1u1i_A

MKVWLvgAYgivSTTAMVGARAIERGIAPKIGLVSELPHFEGIEKYAPFSFEFGGHeirLLSNAYEAAKEhWELNRHFDREILEAVKSDLEGIVARKGTaLNcGSGiKELGDIKTLEGEGLSLAEMVSRIEEDIKSFADDETVVINvastePLPNYSEEYHGSLEGFERMIDEDRKEYASaSMLyAYAALKLGLPYANftPSPGSAIPALKELAEKKGVPHAGNdGKtGETLVKTTLAPMFAYRNMEVVGWMSYNILGdydGKVlSARDNKESkVLSKDKVLEKMLGYSPYSITEIQYFPSLVdNKTAFDFVHFKGFLGKLMKFYFIWDAIdAIVaAPLILDIARFLLFAKKKGVKGVVKEMAFFFKSPMDTNVINTHEQFVVLKEWYSNLK

>1u7h_A

TYFIDVPTXSDLVHDIGVAPFIGELAAALRDDFKRWQAFDKSARVASHSEVGVIELXPVADKSRYAFKYVNgHPANtARNLHtvXAFGVLADVDSGYPVLLSELTIAtALrtAATSLXAAQALARPNARKXALigNgaqSEFQALAFHKHLGIEEIVAydtdPLaTAKLIANLKEYSGLTIRRASSvAEAVKGVDIITTvtadKAYaTiITPDXLEPGXHLNAvgGdCPGkTELHADVLRNARVFVEYEPQTRIEGEIQQLPADFPVVDLWRVLRGETEGRQSDSQVTVFDsvgFALeDYTVLRYVLQQAEKRGXGTKIDLVPWVEDDPkDLFSHTRGRAG

>1u8x_X

KKSFSIVIAgGgstfTPGIVLXLLDHLEEFPIRKLKLydndKErQDRIAGACDVFIREKAPDIEFAATTDPEEAFTDVDFVXAhirvGkyAXRALDEQIPLKYGVVGQeTCGPGGIAyGXRSIGgVLeiLDYXEKYSPDAWXLNysnPaAIVAEATRRLRPNSKILNIcdXPVGIEDRXAQILGLSSRKEXKVRYYGLNHFGWWTSIQDQEGNDLXPKLKEHVSQYGYIPKTSWNDTFAKARDVQAADPDTLPNTYLQYYLFPDDXVKKSNPNHTRANEVXEGreAFIFSQCDXITREQSSENSEIKIDDHASyIVDLARAIAYNTGERXLLIVENNGAIANFDPTAXVEVPCIVGSNGPEPITVGTIPQFQKGLXEQQVSVEKLTVEAWAEKSFQKLWQALILSKTVPNARVARLILEDLVEANKDFWPELDQSP

>1up6_A

HXRIAVigGgsSyTPELVKGLLDISEDVRIDEVIFydidEEkQKIVVDFVKRLVKDRFKVLISDTFEGAVVDAKYVIFqfrpGglKGRENDEGIPLKYGLIGQeTTGVGGFSAALRAFPiVEEyVDTVRKTSNATIVNftnPsGHITEFVRNYLEYEKFIGLcnVPINFIREIAEXFSARLEDVFLKYYGLnHLSFIEKVFVKGEDVTEKVFENLKLKLPDEDFPTWFYDSVRLIVNPYLRYYLXEKKXFKKISTHELRAREVXKIeKELFEKYRTAVEIPEELTKRGgSXySTAAAHLIRDLETDEGKIHIVNTRNNGSIENLPDDYVLEIPCYVRSGRVHTLSQGKGDHFALSFIHAVKXYERLTIEAYLKRSKKLALKALLSHPLGPDVEDAKDLLEEILEANREYVKLG

>1uwk_A

NNKYRDVEIRAPRGNKLTAKSWLTEAPLRMLMNNLDPQVAeNPKELVVyggIGRAARNWECYDKIVETLTRLEDDETLLVQSGKPVGVFKTHSNAPRVLIANSNLVPHWANWEHFNELDAKGLAMYGqMTAGSWIYIGSQGiVQGTYETFVEAGRQHYGGSLKGKWVLTAgLgGmgGAQPLAATLAGACSLNiesqQSrIDFRLETRYVDEQATDLDDALVRIAKYTAEGKAISIALHGnaAEILPELVKRGVRPDMVTDqtsAhDPLNgylPAGWTwEQYRDRAQTEPAAVVKAAKQSMAVHVQAMLDFQKQGVPTFDygnNIRQMAKEEGVADAFDFPGfVPAYIRPLFCRGVGPFRWAALSGEAEDIYKTDAKVKELIPDDAHLHRWLDMARERISFQGLPARICWVGLGLRAKLGLAFNEMVRSGELSAPVVIGRDHlDSGSVSSPNaeTEAMRDGSDAVSDWPLLNALLNTAGGATWVSLHHGGgVGMGFSQHSGMVIVCDGTDEAAERIARVLTNDPGTGVMRHADAGYDIAIDCAKEQGLDLPMITG

>1uxt_A

AGLLEGVIKEKGGVPVYPSYLAGEWGGSGQEIEVKSPIDLATIAKVISPSREEVERTLDVLFKRGRWSARDMPGTERLAVLRKAADIIERNLDVFAEVLVMNAGKPKSAAVGEVKAAVDRLRLAELDLKKIGGDYIPGDWTYDTLETEGLVRREPLGVVAAiTpfnyPLFdAVNKITYSFIYGNAVVVkPsiSDPLPAAMAVKALLDAGFPPDAIALLNLPgkEaeKIvADDRVAAVSftgsTEvGErvVKVGGVKQYVMeLgGGDPAIVLEDADLDLAADKIARGIYSYAGQRcDAIKLVLAERPVYGKLVEEVAKRLSSLRVGDPRDPTVDVGPLISPSAVDEMMAAIEDAVEKGGRVLAGGRRLGPTYVQPTFVEAPADRVKDMVLYKReVfAPVALAVEVKDLDQAIELANGRPYGlDAAVFGRDVVKIRRAVRLLEVGAIYINDMPRhGIGYYPfGGRKKSGVFREGIGYAVEAVTAYKTIVFNYKGKGVWKYE

>1v59_A

TINKSHDVVIIGGGPAGYVAAIKAAQLGFNTACVEKRGKLGGTALNVGAIPSKALLNNSHLFHQMHTEAQKRGIDVNGDIKINVANFQKAKDDAVKQLTGGIELLFKKNKVTYYKGNGSFEDETKIRVTPVDGLEGTVKEDHILDVKNIIVATGSEVTPfPGIEIDEEKIVSSTGALSLKEIPKRLTIigGgiiGLEMGSVYSRLGSKVTVvefqPQIGaSMDGEVAKATQKFLKKQGLDFKLSTkvISAKRNDDKNVVEIVVEDTKTNKQENLEAEVLLVAvgRRPYIAGLGAEKIGLEVDKRGRLVIDDQFNSKFPHIKVVGDVTFGPMLAHKAEEEGIAAVEMLKTGHGHVNYNNIPSVMYSHPEVAWVGKTEEQLKEAGIDYKIGKFPFAANSRAKTNQDTEGFVKILIDSKTERILGAHIIGPNAGEMIAEAGLALEYGASAEDVARVCHAHPTLSEAFKEANMAAYDKAIHC

>1v8b_A

NKSKVKDISLAPFGKMQMEISENEMPGLMRIREEYGKDQPLKNAKITGCLHMTVECALLIETLQKLGAQIRWCSCNIYSTADYAAAAVSTLENVTVFAWKNETLEEYWWCVESALTWGDGDDNGPDMIVDDGGDATLLVHKGVEYEKLYEEKNILPDPEKAKNEEERCFLTLLKNSILKNPKKWTNIAKKIIGVSEEtttGVLRLKKMDKQNELLFTAINVNDAVTKQKYdnVYGcRHSLPDGLMRATDFLISGKIVVIcgYgdvGKGCASSMKGLGARVYIteidPIcAIQAVMEGFNVVTLDEIVDKGDFFITCtgnVDvIKLEHLLKMKNNAVVGNighFDDEIQVNELFNYKGIHIENVKPQVDRITLPNGNKIIVLARGRlLnLGCATGhPAFVMSFSFCNQTFAQLDLWQNKDTNKYENKVYLLPKHLDEKVALYHLKKLNASLtELDDNqCQFlGVNKSGPfkSNEyRY

>1v9l_A

TGFLEYVLNYVKKGVELGGFPEDFYKILSRPRRVLIVNIPVRLDGGGFEVFEGYRVQHCDVLGPYKGGVRFHPEVTLADDVALAILMTLkNSLAGLPYGGAKGAVRVDPKKLSQRELEELSRGYARAIAPLIGDVVDIPAPDVGTNAQIMAWMVDEYSKIKGYNVPGVFTSKPPELWGNPVREYAtGFGVAVATREMAKKLWGGIEGKTVAIqgMgnvGRWTAYWLEKMGAKVIAVsdiNGVAYRKEGLNVELIQKNKGLTgpALVELFTTKDNAEFVKNpdAIFKLDVDIFVPAaieNvIRGDNAGLVKARLVVEGanGPTTPEAERILYERGVVVVPDILAnAGgvIMSYLEWVENLQWYIWDEEETRKRLENIMVNNVERVYKRWQREKGWTMRDAAIVTALERIYNAMKIRGWI

>1vbi_A

MRWRADFLSAWAEALLRKAGADEPSAKAVAWALVEADLRGvGshGLLRLPVYVRRLEAGLVNPSPTLPLEERGPVALLDGEHGFGPRVALKAVEAAQSLARRHGLGAVGVRRSThfGmaGLYAEKLAREGFVAWVTtNaEPDVVPFGGREKALGtNpLAFAAPAPQGILVadlaTSESAMGkVFLAREKGERIPPSWGVDREGSPTDDPHRVYALRPLGGPKGYALALLVEVLSGVLTGAGVAHGIGRMYDEWDRPQDVGHFLLALDPGRFVGKEAFLERMGALWQALKATPPAPGhEEVfLpGEleARRRERALAEGMALPERVVAELKALGERYGVPW

>1vm6_A

HMKYGIvgYsgrmGQEIQKVFSEKGHELVLKvdvNGVEELDSPDVVIDfsspeaLPkTVDLCKKYRAGLVLgttALKEEHLQMLRELSKEVPVVQAYnfSIGINVLKRFLSELVKVLEDWDVEIVETHhRFkkdAPSGTAILLESALGKSVPIHSLRVGGVPGDHVVVFGNIGETIEIKHRAISrTVfAIGALKAAEFLVGKDPGMYSFEEVIFG

>1wdl_A

MIYEGKAITVTALESGIVELKFDLKGESVNKFNRLTLNELRQAVDAIKADASVKGVIVSSGKDVFIVGADITEFVENFKLPDAELIAGNLEANKIFSDFEDLNVPTVAAINGIALGGGLEMCLAADFRVMADSAKIGLPEVKLGIYPGFGGTVRLPRLIGVDNAVEWIASGKENRAEDALKVSAVDAVVTADKLGAAALDLIKRAISGELDYKAKRQPKLEKLKLNAIEQMMAFETAKGFVAGQAGPNYPAPVEAIKTIQKAANFGRDKALEVEAAGFAKLAKTSASNCLIGLFLNDQELKKKAKVYDKIAKDVKQAAVlgAgimGGGIAYQSASKGTPILMkdinEHgIEQGLAEAAKLLVGRVDKGRMTPAKMAEVLNGIRPTLSyGDFGNVDLVVeavveNPKvkQAvLAeVENHVREDAILASntsTISISLLAKALKRPENFVGMhFFnPVHMMPLVEVIRGEKSSDLAVATTVAYAKKMGKNPIVVNDCPGFLVNRVLFPYFGGFAKLVSAGVDFVRIDKVMEKFGWPMGPAYLMDVVGIDTGHHGRDVMAEGFPDRMKDDRRSAIDALYEAKRLGQKNGKGFYAYEADKKGKQKKLVDSSVLEVLKPIVYEQRDVTDEDIINWMMIPLCLETVRCLEDGIVETAAEADMGLVYGIGFPLFRGGALRYIDSIGVAEFVALADQYAELGALYHPTAKLREMAKNGQSFFG

>1wnb_A

MQHKLLINGELVSGEGEKQPVYNPATGDVLLEIAEASAEQVDAAVRAADAAFAEWGQTTPKVRAECLLKLADVIEENGQVFAELESRNCGKPLHSAFNDEIPAIVDVFRFFAGAARCLNGLAAGEYLEGHTSMIRRDPLGVVASiApwnYPLMMAAWKLAPALAAGNCVVLkPseiTPLTALKLAELAKDIFPAGVVNILFGRgkTVgdPLtGHPKVRMVSltgsIAtGEhiISHTASSIKRTHMeLgGKAPVIVFDDADIEAVVEGVRTFGYYNAGQDcTAACRIYAQKGIYDTLVEKLGAAVATLKSGAPDDESTELGPLSSLAhLErVGKAVEEAKATGHIKVITGGEKRKGNGYYYAPTLLAGALQDDAIVQKeVfGPVVSVTPFDNEEQVVNWANDSQYGLASSVWTKDVGRAHRVSARLQYGCTWVNTHFMLVSEMPHGGQKLSGYGKDMSLYGLEDYTVVRHVMVKH

>1x0x_A

SMASKKVCIVGsgnwgSAIAKIVGGNAAQLAQFDPRVTMwvfEeDIGGKKlTEIINTQHENVKyLPGHKLPPNVVAVPDVVQAAEDADILIFvvpHQfIGKiCDQLKGHLKANATGISLikGVDEGPNGLKLISEVIGERLGIPMSVLMGAniaSEVADEKFCETTIGCKDPAQGQLLKELMQTPNFRITVVQEVDTVEICGALknVVAVGAGFCDGLGFGDNTKAAVIRLGLMEMIAFAKLFCSGPVSSATFLESCGVADLITTCYGGrNRKVAEAFARTGKSIEQLEKELLNGqkLqGPETARELYSILQHKGLVDKFPLFMAVYKVCYEGQPVGEFIHCLQNHPEHM

>1xag_A

MKLQTTYPSNNYPIYVEHGAIKYIGTYLNQFDQSFLLIdEyvNQyfANKFDDILSYENVHKVIIPageKTkTFEQYQETLEYILSHHVTRNTAIIAVGggaTGdFAGFVAATLLRGVHFIQVPttIlAHdsSVGGkvGINSKQGknLIGAFYRPTAVIYDLDFLKtlpFKqILsGYAEVYKHALLNGESATQDIEQHFKDREILQSLNGMDKYIAKGIETkLDIVVADEKEQGVRKFLNLGHTFGHAVEYYHKIPhGHAVMVGIIYQFIVANALFDSKHDISHYIQYLIQLGYPLDMITDLDFETLYQYMLSDKKNDKQGVQMVLMRQFGDIVVQHVDQLTLQHACEQLKTYF

>1y3i_A

VLLVVhLVLVLGGdgTFlRAAELARNASIPVLGVNLGrigflAEAEAEAIDAVLEHVVAQDYRVEDRLTLDVVVRQGGRIVNRGWALneVSLeKGPRLGVLGVVVEIDGRPVSAFGCDGVLVSTPTGStayAFsAGGPVLWPDLEAILVVPNNAHALFGRPMVTSPEATIAIEIEADGHDALVFCDgrREMLIPAGSRLEVTRCVTSVKWARLDSAPFTDRLVRKfRLPVT

>1y9e_A

STLFQALQAEKNADDVSVHVKTISTEDLPKDGVLIKVAYSGINYKDGLAGKAGGNIVREYPLILGIDAAGTVVSSNDPRFAEGDEVIATSYELGVSRDGGLSEYASVPGDWLVPLPQNLSLKEAXVYGTAGFTAAlSVHrLEQNgLSPEKGSVLVTGATGGVGGIAVSXLNKRGYDVVASTGNREAADYLKQLGASEVISREDVYDGTLKALSKQQWQGAVDPVGGKQLASLLSKIQygGSVAVSGLTGGGEVPATVYPFILRgVsLLGidSVYcpXDvRAAVWERXSSDLKPDQLLTIVDREVSLEETPGALKDILQNRIQGRVIVKL

>1yba_A

SLEKDKIKFLLVEGVHQKALESLRAAGYTNIEFHKGALDDEQLKESIRDAHFIGLRSRTHLTEDVINAAEKLVAIGCFCiGTNQVDLDAAAKRGIPVFNAPfSntRSvAELVIGELLLLLRGVPEANAKAHRGVWNKLAAGSFEARGKKLGIigYghiGTQLGILAESLGXYVYFydieNkLPLGNATQVQHLSDLLNXSDVVSLhvpEnPstKNXXGAKEISLXKPGSLLINasrGTVVDIPALCDALASKHLAGAAIdvFPTEPATNSDPFTSPLCEFDNVLLTPhIggSTQEAQENIGLEVAGKLIKYSDNGSTLSAVNFPEVSLPLHGGRRLXHIHENRPGVLTALNKIFAEQGVNIAAQYLQTSAQXGYVVIDIEADEDVAEKALQAXKAIPGTIRARLLY

>1z0z_A

MRAAVVYKTDGHVKRIEEALKRLEVEVELFNQPSEELENFDFIVSVGGdgTIlrILQKLKRCPPIFGINTGRVGllTHASPENFEVELKKAVEKFEVERFPRVSCSAMPDVLALneIAVLSRKPAkmIDVALRVDGVEVDRIrCdGFIVATQIgStgyAFsAGGPVVEPYLECFILIPIAPfRFGWKPYVVSMERKIEVIAEKAIVVAdgqKSVDFDGEITIEKSEFPAVFFKNEKRFRNLFGKVRSiG

>1z2i_A

TVLARLDELERFCRAVFLAVGTDEETADAATRAMMHGTRLGvDshGVRLLAHYVTALEGGRLNRRPQISRVSGFGAVETIDADHAHGARATYAAMENAMALAEKFGIGAVAIRNSShfGpaGAYALEAARQGYIGLAFcNsDSFVRLhdGAMRFHGtNpIAVGVPAADDMPWLldmaTSAvpYNRVLLYRSLGQQLPQGVASDGDGVDTRDPNAVEMLAPVGGEfGfkGaALAGVVEIFSAVLTGMRLSFDLAPMGGPDFSTPRGlGAFVLALKPEAFLERDVFDESMKRYLEVLRGSPAREDCKVmApGDreWAVAAKREREGAPVDPVTRAAFSELAEKFSVSPPTYH

>1z45_A

SKIVLVTgGAgyiGSHTVVELIENGYDCVVAdnLsnsTYDSVARLEVLTKHHIPFYEVdlcDRKGLEKVFKEYKIDSVIHfaglkAVGESTQIPLRYYHnNILgTVVLLELMQQYNVSKFVFsSsATVYGDATRFPNMIPIPEECPLGPTNPyGHTkYAIENILNDLYNSDKKSWKFAILRyFnpIGAHPSGLIGeDPLGIPNnLLPYMAQVAVGRREKLYIFRDGTPIRDYIHVVDLAKGHIAALQYLEAYNENEGLCREWNLGSGKGSTVFEVYHAFCKASGIDLPYVLNLTAKPDRAKRELKWQTELQVEDSCKDLWKWTTENPFGYQLRGVEARFSAEDMRYDARFVTIGAGTRFQATFANLGASIVDLKVNGQSVVLGYENEEGYLNPDSAYIGATIGRYANRISKGKFSLCNKDYQLTVNNGVNANHSSIGSFHRKRFLGPIIQNPSKDVFTAEYMLIDNEKDTEFPGDLLVTIQYTVNVAQKSLEIVYKGKLTAGEATPINLTNHSYFNLNKPYGDTIEGTEIMVRSKKSVDVDKNMIPTGNIVDREIATFNSTKPTVLGPKNPQFDCCFVVDENAKPSQINTLNNELTLIVKAFHPDSNITLEVLSTEPTYQFYTGDFLSAGYEARQGFAIEPGRYIDAINQENWKDCVTLKNGETYGSKIVYRFS

>1zbq_A

SPLRFDGRVVLVTgAGaglGRAYALAFAERGALVVVndlggDfKgVGKGSLaADKVVEEIRRRGGKAVANYDsveEGEKVVKTALDAFGRIDVVVNnagiLRDRSFARISDEDWDIIHRvhLRGSFQVTRAAWEHMKKQKYGRIIMtSsASGIYGNFGQANySAAkLGLLGLANSLAIEGRKSNIHCNTIApnaGsrmtQTVmPEDLVEAlKPEYVAPLVLWLCHESCEENGGLFEVGAGWIGKLRWERTLGAIVRQKNHPMTPEAVKANWKKICDFENASKPQSIQESTGSIIEVLSKIDS

>1zem_A

KKFNGKVCLVTgAGgniGLATALRLAEEGTAIALldmnREaLEKAEASVREKGVEARSYVCdvtSEEAVIGTVDSVVRDFGKIDFLFNnagyqGAFAPVQDYPSDDFARVLTiNVTGAFHVLKAVSRQMITQNYGRIVNtAsMAGVKGPPNMAAyGTSkGAIIALTETAALDLAPYNIRVNAISpGYmGPGfmwERqVELQAKVGSQYFSTDPKVVAQQMIGSVPMRRYGDiNEIPGVVAFLLGDDSSFMTGVNLPIAGG

>1zjz_A

SNRLDGKVAIITgGtlgiGLAIATKFVEEGAKVMItdrHSDVGEKAAKSVGTPDQIQFFQHdsSDEDGWTKLFDATEKAFGPVSTLVNnagiAVNKSVEETTTAEWRKLLAvNLDGVFFGTRLGIQRMKNKGLGASIINmSsiEGFVGDPSLGAyNASkGAVRIMSKSAALDCALKDYDVRVNTVHpGyiKtPlvDDLPGAEEAmSQRTKTPMGHIGEPNDIAYICVYLASNESKFATGSEFVVDGGYTAQ

>1zrq_B

FFHASQRDALNQSLAEVQGQINVSFeFfpPRTSEMEQTLWNSIDRLSSLKPKFVSVtYgANSGeRDRTHSIIKGIKDRTGLEAAPHLTCIDATPDELRTIARDYWNNGIRHIVALRGdLPPEMYASDLVTLLKEVADFDISVAAYPEVHPEAKSAQADLLNLKRKVDAGANRAITqfFFDVESYLRFRDRCVSAGIDVEIIPGIlPVSNFKqAKKfADmtNVRIPAWMAQMFDGLDDDAETRKLVGANIAMDMVKILSREGVKDFHFyTlNRAEMSYAICHTLGVRP

>2a5f_B

DKLYradsrPPDeIKQSGGLmPrGQsEYFDRGTQMNINLYDHaRGTQTGFvRHDDGYvstsISLRSaHLvGQTilSGHSTYYIYVIATAPNMFNVNDVLGAYSPHPDdQdVSALGGIPYSQIYGWYRVHFGVLDEQLHRNRGYRDRYYSNLDIAPAADGYGLAGFPPEHRAWREEPWIHHAPPGS

>2ag5_A

MGRLDGKVIILtaAaqgiGQAAALAFAREGAKVIAtdinESKLQELEKYPGIQTRVLdvtKKKQIDQFANEVERLDVLFNvaGFvHHGTVLDCEEKDWDfSMNlNVRSMYLMIKAFLPKMLAQKSGNIINmSsVASSVKGVVNRCVySTTkAAVIGLTKSVAADFIQQGIRCNCVCpGtvDtpslQErIQARGNPEEARNDFLKRQKTGRFATAEEIAMLCVYLASDESAYVTGNPVIIDGGWSLG

>2b69_A

RKRILITgGAgfvGSHLTDKLXXDGHEVTVVdnFftgRKRNVEHWIGHENFELINHdvVEPLYIEVDQIYHlaspaSPPNYXYNPIKTLKtNTIGTLNXLGLAKRVGARLLLaStsEVYGDPEVHPQSEDYWGHVNPIGPRACyDEGkRVAETXCYAYXKQEGVEVRVARiFntFGPRXHXNdGrVVSNFILQALQGEPLTVYGSGSQTRAFQYVSDLVNGLVALXNSNVSSPVNLGNPEEHTILEFAQLIKNLVGSGSEIQFLSEAQDDPQKRKPDIKKAKLXLGWEPVVPLEEGLNKAIHYFRKELEYQA

>2bi4_A

MANRMILNETAWFGRGAVGALTDEVKRRGYQKALIVTdKtlVQcGvVAKVTDKMDAAGLAWAIYDGVVpnpTITVVKEGLGVFQNSGADYLIAIGggspQdTCKAIGIISNNPEFADVRSLEGLSPTNKPSVPILAIPttAGtAAEVTInYvITDEEKRRkFvCVDPHDIPQVAFIDADMmDgmpPAlKAAtGVdALThAIEGYITRGAWALTDALHIKAIEIIAGALRGSVAGDKDAGEEMALGQYVAGMGfSNVGlGLVhGMAhPLGAFYNTPhgVANAILLPHVMRYNADFTGEKYRDIARVMGVKVEGMSLEEARNAAVEAVFALNRDVGIPPHLRDVGVRKEDIPALAQAALDDVcTGGNPREATLEDIVELYHTAW

>2c20_A

NSILICgGAgyiGSHAVKKLVDEGLSVVVVdnLqtgHEDAITEGAKFYNGdlrDKAFLRDVFTQENIEAVMhfaaDsLVGVSMEKPLQYYNnNVYGALCLLEVMDEFKVDKFIFsStAATYGEVDVDLITEETMTNPTNTyGETkLAIEKMLHWYSQASNLRYKIFRyFnvAGATPNGIIGEDhRPEThLIPLVLQVALGQREKIMMFGDDYNTPDGTCIRDYIHVEDLVAAHFLGLKDLQNGGESDFYNLGNGNGFSVKEIVDAVREVTNHEIPAEVAPRRAGDPARLVASSQKAKEKLGWDPRYVNVKTIIEHAWNWHQKQPNGYEK

>2c59_A

YTYKELEREQYWPSENLKISITgAGgfiASHIARRLKHEGHYVIASdwkKNEHMTEDMFCDEFHLVdlrVMENCLKVTEGVDHVFNlaadmGGMGFIQSNHSVIMYnNTMiSFNMIEAARINGIKRFFyaSsACIYPEFKQLETTNVSLKESDAWPAEPQDAyGLEkLATEELCKHYNKDFGIECRIGRfHniYGPFGtWKGGrEkAPAAFCRKAQTSTDRFEMWGDGLQTRSFTFIDECVEGVLRLTKSDFREPVNIGSDEMVSMNEMAEMVLSFEEKKLPIHHIPGPEGVRGRNSDNNLIKEKLGWAPNMRLKEGLRITYFWIKEQIEKEKAKGSDVSLYGSSKVVGTQAPVQLGSLRAADG

>2czc_A

MKVKVGVngYgtiGKRVAYAVTKQDDMELIGItktkPDFEAYRAKELGIPVYAasEEfIPRFEKEGFEVAGTlNDLLEKVDIIVDatpgGiGAKnKPLYEKAGVKAIFqGgeKADVAEVSFVAQANYEAALGKNYVRVVscNTTGLVRTLSAIREYADYVYAVMIRrAAdPNDTKRGPINAIKPTVEVPSHHGPDVQTVIPINIETMAFVVPTTLMHVHSVMVELKKPLTKDDVIDIFENTTRVLLFEKEKGFDSTAQIIEFARDLHREWNNLYEIAVWKESINIKGNRLFYIQAVHqeSDvIPENIDAIRAMFELADKWDSIKKTNKSLGILK

>2d1y_C

GLFAGKGVLVTgGArgiGRAIAQAFAREGALVALCdlrPEGKEVAEAIGGAFFQVdleDERERVRFVEEAAYALGRVDVLVNnaaiAAPGSALTVRLPEWRRVLEvNLTAPMHLSALAAREMRKVGGGAIVNvAsvQGLFAEQENAAyNASkGGLVNLTRSLALDLAPLRIRVNAVApGaiAtEavLEAIALSPDPERTRRDWEDLHALRRLGKPEEVAEAVLFLASEKASFITGAILPVDGGMTASF

>2d37_A

MAEVikSImrKFPlGVAIVTTNWKGELVGMTVntFNSLSLNPPLVSFfAdRMkGNDIPYKESKYFVVNFTDNEELFNIFALKPVKERFREIKYKEGIGGCPILYDSYAYIEAKLYDTIDVGdhSIIVGEVIDGYQIRDNFTPLVyMNrKYYKLSS

>2d4e_C

MRYADRVAGISWETIEEVRRRLKERPALHFIAGEFVPSESGETFPSLDPATNEVLGVAARGGEREVDRAAKAAHEAFQRWSRTKAKERKRYLLRIAELIEKHADELAVMECLDAGQVLRIVRAQVARAAENFAFYAEYAEHAMEDRTFPVDRDWLYYTVRVPAGPVGIiTpwnAPLMlSTWRIAPALAFGNTVVLkPaeWSPFTATKLAEILKEADLPPGVFNLVQGFgeEAgaALvAHPLVPLLTltgeTEtGKivMRnAADHLKRLSPeLgGKSPALVFADADLERALDAVVFQIFSFNGERcTASSRLLVEEKIFEDFVGKVVERARAIRVGHPLDPETEVGPLIHPEHLQRVLGYVEAGKREGARLLVGGERAKTSFRGEDLSRGNYLLPTVFVGENHMKIAQEeIfGPVLVAIPFKDEEEALRKANDTKYGlAAYVFTRDLERAHRLALELEAGMVYLNSHNVRHLPTPfgGVKGSGDRREGGTYALDFYTDLKTIALPLRPPHVPKFGK

>2d4v_A

THIQKPATGSPLTLLNGVLQVPDQPIIPFIEGDGiGCDVTPAMRSVVDAAVAKVYGGQRQIAWMELFAGQKAVQLYGEGQYLPDETMAAIREYKVAIkGpLetPvGGGIRSLnVAMrQDLDLYVCLRPVRYFEGTPSPMRHPEKVDMVIFRENSEDIYAGIEWPAGSPEAEKIIRFLREEMGVTKIRFPDSSAIGIKPVSTEGSERLIRRTIQYALEHGKPSVSLVHKGnIMKFTEGGFRDWGYALAEREFAGRVFTWRQKAAISKAEGKAAGQKAEQQAIADGKLIIKDViAdnFLqqILLrPEDYSVVATLNLNGDYVSDALAAEVGGigMAPGANLSDTHAIFeAThgtapdiAGQGKanPSSLILSAVMMLEHLGWGEAAQAIVAAMNATIAAGEVTGdLAALRGDVPALSTTEFTAALIRRF

>2dc1_A

MLVGLigYgaiGKFLAEWLERNGFEIAAIldvrGEHEKMVRGIDEFLQREMDVAVEaasqqaVKdyAEKILKAGIDLIVlstGAFADRDFLSRVREVCRKTGRRVYIASgAIGGLDAIFSASELIEEIVLTTRKNWRQFGRKGVIFEGSASEAAQKFPkNLnvAAtLSIASGKDVKVRLVADEVEENIHEILVRGEFGEMEIRVRNRPMREnPKtSYLaALSVTRILRNLKEGLVV

>2dfd_A

NAKVAVLgAsggiGQPLSLLLKNSPLVSRLTLydiaHTPGVAADLSHIETKAAVKGYLGPEQLPDCLKGCDVVVIpagvpRkPGMTRDDlFNTnATiVAtlTAACAQHCPEAMICVianPvNSTIPITAEVFKKHGVYNPNKIFGVtTlDIVRANTFVAELKGLDPARVNVPVIGGhAGKTIIPLISQCTPKVDFPQDQLTALTGRIQEAGTEVVKAKAGAgsatLSmAYAGARFVFSLVDAMNGKEGVVECSFVKSQETECTYFSTPLLLGKKGIEKNLGIGKVSSFEEKMISDAIPELKASIKKGEDFVKTL

>2dfv_A

EKXVAIXKTKPGYGAELVEVDVPKPGPGEVLIKVLATSIcgTDLhIYEWNEWAQSRIKPPQIXGHEVAGEVVEIGPGVEGIEVGDYVSVETHIVCGKCYACRRGQYHVCQNTKIFGVDTDGVFAEYAVVPAQNIWKNPKSIPPEYATLQEPLGnAVDTVLAGPISGKSVLItgAgplGLLGIAVAKASGAYPVIVsepsDFrRELAKKVGADYVINpfEEDVVKEVXDITDGNGVDVFLEFsGaPkaLEqGLQAVTPAGRVSLlglYPGKVTIDFNNLIIFKALTIYGItGRHLWETWYTVSRLLQSGKLNLDPIITHKYKGFDKYEEAFELXRAGKTGKVVFXLK

>2dld_A

MTKVFAYAIRKDEEPFLNEWKEAHKDIDVDYTDKLLTPETAKLAKGADGVVVYQQLDYTADTLQALADAGVTKMSLRNvGVDNIDMDKAKELGFQITNVPVySPNAiAEHAAIQAARVLRQDKRMDEKMAKRDLRWAPTIGREVRDQVVGVvgTghiGQVFMRIMEGFGAKVIAydifKNPELEKKGYYVDSLDDLYKQADVISLhvpDvPanVHMINDKSIAEMKDGVVIVNCsrGRLVDTDAVIRGLDSGKIFGFVMdtYEDEVGVFNKDWEGKEFPDKRLADLIDRPNVLVTPhTafYTTHAVRNMVVKAFNNNLKLINGEKPDSPVALNKNKf

>2dph_A

AGNKSVVYHGTRDLRVETVPYPKLEHNNRKLEHAVILKVVSTNIcgsDqhIYRGRFIVPKGHVLGHEITGEVVEKGSDVELMDIGDLVSVPfNVACGRCRNCKEARSDVCENNLVNPDADLGAFGfDLKGWSGGQAEYVLVPYADYMLLKFGDKEQAMEKIKDLTLISdiLPtGFHGCVSAGVKPGSHVyIagAgpvGRCAAAGARLLGAACVIVGdqnPErLKLLSDAGFETIDlRNSAPLRDQIDQILGKPEVDCGVDAvgFEahGlGDEANTETPNGaLNsLFDVVRAGGAIGIpgiYVGSDPDPVNKDAGSGRLHLDFGKMwTKSIRIMTGmaPVTNYNRHLTEAILWDQMPYLSKVMNIEVITLDQAPDGYAKfDKGSPAKFVIDPHGMLKNK

>2dt5_B

MKVPEAAISRLITYLRILEELEAQGVHRTSSEQLGGLAQVTAFQVRKDLSYFGSYGTRGVGYTVPVLKRELRHILGLNRKWGLCIvgMgrlGSalAdypGFGESFELRGFFdvdPEkVGRPVRGGVIEHvDLLPQRVPGRIEIALLtvprEaAQkaADlLVAAGIKGILNfapVVLEVPKEVAVENvDfLAGLTRLSFAILNPKWREEMMG

>2dvm_A

IREKALEFHKNNFPGNGKIEVIPKVSLESREELTLAYTPGVAEPCKEIARDPGKVYEYTSKGNLVAVVSDGSrILgLGNIGPLAGLPVMEGKALLFKRFGGVDAFPIMIKEQEPNKFIDIVKAIAPTFGGINLEDIAsPKCFYILERLREELDIPVFHDdQQGtAAVVLAGLLNALKVVGKKISEITLALfgAgaaGFATLRILTEAGVKPENVRVvelVNGKpRILTSDLDLEKLfPYrGWLLKKTNGENIEGGpQEALKDADVLISftrpGPGvIKPQWIEKMNEDAIVFPlAnPVPEILPEEAKKAGARIVATgRSDYPNQiNnLLGFPGIFRGALDVRARTITDSMIIAAAKAIASIVEEPSEENIIPSPLNPIVYAREARAVAEEAMKEGVARTKVKGEWVEEHTIRLIEFYENVIAPINKKRREYSKAITRA

>2e37_C

XKVGIvgSgXvGSATAYALALLGVAREVVLvdldRKlAQAHAEDILHATPFAHPVWVRAGSyGDLEGARAVVLaagvaQRpGETRLQlLDRnAQvFAqvVPRVLEAAPEAVLLVAtnPVDVXTQVAYRLSGLPPGRVVGSGTILDTARFRALLAEYLRVAPQSVHAYVLGEHGDSEVLVWSSAQVGGVPLLEFAEARGRALSPEDRARIDEGVRRAAYRIiEGKGatYYGIGAGLARLVRAILTDEKGVYTVSAFTPEVEGVLEVSLSLPRILGAGGVEGTVYPSLSPEEREALRRSAEILKEAAFALGF

>2ed4_A

MKEAfkEAlaRFASGVTVVAARLGEEERGMTAtaFMsLSlEPPLVALAVSERAKLLPVLEGAGAFTVSLLREGQEAVSEHFAGrPKEGIALEEGRVKGALAVLRCRLHALYPGGdhRIVVGLVEEVELGEGGPPLVyFQrGYRRLVWPS

>2ehu_A

MTVEPFRNEPIETFQTEEARRAMREALRRVREEFGRHYPLYIGGEWVDTKERMVSLNPSAPSEVVGTTAKAGKAEAEAALEAAWKAFKTWKDWPQEDRSRLLLKAAALMRRRKRELEATLVYEVGKNWVEASADVAEAIDFIEYYARAALRYRYPAVEVVPYPGEDNESFYVPLGAGVViApwnfPVAiFTGMIVGPVAVGNTVIAkPaeDAVVVGAKVFEIFHEAGFPPGVVNFLPGVgeEVgaYLVEHPRIRFINftgsLEvGLkiYEAAGRLAPGQTWFKRAYVeTgGKDAIIVDETADFDLAAEGVVVSAYGFQGQKcSAASRLILTQGAYEPVLERVLKRAERLSVGPAEENPDLGPVVSAEqERkVLSYIEIGKNEGQLVLGGKRLEGEGYFIAPTVFTEVPPKARIAQEeIfGPVLSVIRVKDFAEALEVANDTPYGlTGGVYSRKREHLEWARREFHVGNLYFNRKITGALVGVQPfgGFKLSGTNAKtGALDYLRLFLEMKAVAERFS

>2ekl_A

AIYTVKALITDPIDEILIKTLREKGIQVDYMPEISKEELLNIIGNYDIIVVRSRTKVTKDVIEKGKKLKIIARAGiGLDNIDTEEAEKRNIKVVYAPgAstDSaVELTIGLMIAAARKMYTSMALAKSGIFKKIEGLELAGKTIGIvgFgriGTKVGIIANAMGMKVLAydilDiREKAEKINAKAVSLEELLKNSDVISLhvtvSKDAKPiIDYPQFELMKDNVIIVNtsrAVAVNGKALLDYIKKGKVYAYATdvFWNEPPKEEWELELLKHERVIVTThIgaQTKEAQKRVAEMTTQNLLNAMKELGMI

>2ewm_B

QRLKDKLAVITgGangiGRAIAERFAVEGADIAIAdlvPAPEAEAAIRNLGRRVLTVKCdvSQPGDVEAFGKQVISTFGRCDILVNnagiyPLIPFDELTFEQWKKTFEiNVDSGFLMAKAFVPGMKRNGWGRIINlTsTTYWLKIEAYTHyISTkAANIGFTRALASDLGKDGITVNAIApSlvRtattEASAlSAMFDVLPNMLQAIPRLQVPLDLTGAAAFLASDDASFITGQTLAVDGGMVRH

>2fm3_A

MIERRKIAVigSgqiGGNIAYIVGKDNLADVVLfdiaEGiPQGKALDITHSMVMSTSKVIGTNDyADISGSDVVIItasiDDRSElLFGnARiLDsvAEGVKKYCAFVICitnPlDVMVSHFQKVSGLPHNKVCGmaGVlDSSRFRTFIAQHFGVNASDVSANVIGGhGDGMVPATSSVSVSFIKQGLITQEQIDEIVCHTRIAWKEVNLGtAyFApAAAAVKMAEAYLKDKKAVVPCSAFCSNHYGVKGIYMGVPTIIGKNGVEDILELDLTPLEQKLLGESINEVNTISKVLDNAP

>2g5c_B

QNVLIvgVgfXGGSFAKSLRRSGFKGKIYGydinPEsISKAVDLGIIDEGTTSIAKVEDFSPDFVXLsspvRtfREiAKKLSYILSEDATVTDqgsVKGKLVYDLENILGKRFVGGhPIAGteKsgVEYSLDNLYEGKKVILTPTKKTDKKRLKLVKRVWEDVGGVVEYXSPELHDYVFGVVSHLPHAVAFALVDTLIHXSTPEVDLFKYPGGGFKDFTRIAKSdPIXWRDiFLENKENVXKAIEGFEKSLNHLKELIVREAEEELVEYLKEVKIKRXEI

>2g76_A

LRKVLISDSLDPCCRKILQDGGLQVVEKQNLSKEELIAELQDCEGLIVRSATKVTADVINAAEKLQVVGRAGtgVDNVDLEAATRKGILVMNTPNGnsLSaAELTCGMIMCLARQIPQATASMKDGKWERKKFMGTELNGKTLGIlgLgriGREVATRMQSFGMKTIGydpiiSPEVSASFGVQQLPLEEIWPLCDFITVhtpLlPstTGlLNDNTFAQCKKGVRVVNcarGGIVDEGALLRALQSGQCAGAALdvFTEEPPRDRALVDHENVISCPhLgASTKEAQSRCGEEIAVQFVDMV

>2g8y_A

SGHRFDAQTLHSFIQAVFRQXGSEEQEAKLVADHLIAANLAGhDShGIGXFPSYVRSWSQGHLQINHHAKTVKEAGAAVTLDGDRAFGQVAAHEAXALGIEKAHQHGIAAVALHNSHhIGriGYWAEQCAAAGFVSIHFvSvVGIPXVAPfhGRDSRFGtNpFCVVFPRKDNFPLLldyaTSAIaFGkTRVAWHKGVPVPPGCLIDVNGVPTTNPAVXQESPLGSLLTFAEhkGyaLAAXCEILGGALSGGKTTHQETLQTSPDAILnCXTTIIINPELFGAPDCNAQTEAFAEWVKASPHDDDKPIlLpGEweVNTRRERQKQGIPLDAGSWQAICDAARQIGXPEETLQAFCQQLAS

>2gag_A

MSKPQRLSAEQSSRARINREEALSLTVDGAKLSAFRGDTVASALLANGVRRAGNSLYLDRPRGIFAAGVEEPNALVTVSARHEQDIDESMLPATTVPVTEDLNATLLSGLGVLDPTKDPAYYDHVHVHTDVLVvgAgpaGLAAAREASRSGARVMLLderAEAGgtlLDtAGeQIDGMDSSAWIEQVTSELAEAEETTHLQRTtvFGSYDANYLIAAQRRTVHLDGPSGPGVSRERIWHIRAKQVVLAtgahERPIVFENNDRPGIMLAGAVrSYLNRYGVRAGARIAVATTnDsAYeLVRELAATGGVVAVIDARSSISAAAAQAVADGVQVISGSVVVDTEADENGELSAIVVAELDEARELGGTQRFEADVLAVAGGfNPvVHlHSQRQGKLDWDTTIHAFVPADAVANQHLAgaMTGRldtASaLSTGAATGAAAATAAGFATVARTPQALETALGETRPVWLVPSVSGDDAVNYKFHFVDLQRDQTVADVLRATGAGMKSVEHIKRYTSISTANDQGKTSGVAAIGVIAAVLGIENPAAIGTTTFRAPyTPVAFAALAGRNRGDQLDPARITAMHSWHLSHGAEFEDVGQWKRPWYYPQAGETMDQAVYRESKAVRDSVGMLDATTLGKIEIRGKDAAEFLNRIYTNGYTKLKVGMGRYGVMCKADGMIFDDGVTLRLAEDRFLLHTTTGGAADVLDWLEEWLQTEWPDLDVTCTSVTEQLATVAVVGPRSRDVIAKLASTVDVSNEGFKFMAFKDVVLDSGIEARISRISFSGELAFEIAVPAWHGLRVWEDVYAAGEEFNITPYGTETMHVLRAEKGFIIVGQDTDGTVTPQDAGMEWVVSKLKDFIGNRSYSRADNAREDRKQLVSVLPVDKSLRLPEGAALVASDALASEGITPMEGWVTSSYDSPNLGRTFGLALIKNGRNRIGEVLKTPVGDQLVDVVVSETVLYDPEGSRRDG

>2gdz_A

AHMVNGKVALVTgAaqgiGRAFAEALLLKGAKVALVdwnLEAGVQCKAALHEQFEPQKTLFIQCdvADQQQLRDTFRKVVDHFGRLDILVNnagvNNEKNWEKTLQiNLVSVISGTYLGLDYMSKQNGGEGGIIINmSsLAGLMPVAQQPVyCASkHGIVGFTRSAALAANLMNSGVRLNAICpGfvNtailESIEKEENMGQYIEYKDHIKDMIKYYGILDPPLIANGLITLIEDDALNGAIMKITTSKGIHFQDYGSKENLYFQ

>2gr9_A

GMSVGFIGAGQLAFALAKGFTAAGVLAAHKIMASSPDMDLATVSALRKMGVKLTPHNKETVQHSDVLFLAVKPHIIPFILDEIGADIEDRHIVVSAAAGVTISSIEKKLSAFRPAPRVIRAMTNTPVVVreGATVYATGTHAQVEDGRLMEQLLssVgfCTEVEEDLIDAVTGLSGSGPAYAFTALDALADGGVKMGLPRRLAVRLGAQALLGAAkMLlhSeQHPGQLKDNVSSPGGATIHALHVLESGGFRSLLINAVEASCIRTRELQSMADQE

>2gru_A

TTKQIAFADRAFNFAFGEHVLESVESYIPRDEFDQYIMISdSgvpDSiVHYAAEYFGKLAPVHILRFQgGeEYkTLSTVTNLQERAIALGANRRTAIVAVGgglTGnVAGVAAGMMFrGIALIHVPttFlaASdsVLsIkqAVNLTSGKnLVGFYYPPRFVFADTRiLSEspPRqVKaGMCELVKNMLILENDNKEFTEDDLNSANVYSPKQLETFINFCISAKMSVLSEDIYEKKKGLIFEYGHTIGHAIELAEQGGIThGEAIAVGMIYAAKIANRMNLMPEHDVSAHYWLLNKIGALQDIPLKSDPDSIFHYLIHDNkRGYIKLDEDNLGMILLSGVGKPAMYNQTLLTPVRKTLIKEVIREGL

>2gsd_A

AKVVCVLYDDPINGYPTSYARDDLPRIDKYPDGQTLPTPKAIDFTPGALLGSVSGELGLRKYLESQGHELVVTSSKDGPDSELEKHLHDAEVIISQPfWPAYLTAERIAKAPKLKLALTAGigSdHVDLQAAIDNNITVAeVTYCnsNSvAEHVVMMVLGLVRNYIPSHDWARNGGWNIADCVARSYDVEGMHVGTvaAgriGLRVLRLLAPFDMHLHYtdrhRLPEAVEKELNLTWHATrEDMYGACDVVTLncpLhPetEHMINDETLKLFKRGAYLVNtarGKLCDRDAIVRALESGRLAGYAGdvWFPQPAPNDHPWRTMPHNGMTPhIsgTSLSAQTRYAAGTREILECYFEGRPIRDEYLIVQGGGLAGvGAhsySKGNATGGSEEAAKYEKL

>2gwl_A

SKQIQALRYYsAQGySVInKYLrGdDYPETQAKETLLSRDYLSTNEPSDEEFKNAMSVYINDIAEGLSSLPETDHRVVYrglklDkPAlSDvLKEyTTIGNIIIdKAFMsTsPDKawInDTILNIYLEKGHKGRILGDVAHFKGeAeMLFPPNTKLKIESIVNCGSQDFASQLSKLRLSDDATADTNRIKRIINMRVLNS

>2hdh_A

KIIVKHVTVigGglXGAGIAQVAAATGHTVVLvdqTEDiLAKSKKGIEESLRKVAKKKFAENPKAGDEFVAKTLSTIATSTDAASVVHSTDLVVeaiveNLKvkNElFKRLDKRAAEHTIFASntsSLQITSIANATTRQDRFAGLhFfnPVPVXKLVEVIKTPXTSQKTFESLVDFSKALGKHPVSCKDTPGFIVnRLLVPYLXEAIRLYERGDASKEDIDTAXKLGAGYPXGPFELLDYvGLDtTKFIVDGWHEXDAENPLHQPSPSLNKLVAENKFGKKTGEGFYKYKAA

>2hsd_A

NDLSGKTVIItgGarglGAEAARQAVAAGARVVLAdvlDEEGAATARELGDAARYQHldvtIEEDWQRVVAYAREEFGSVDGLVNnagisTGMFLETESVERFRKVVEiNLTGVFIGMKTVIPAMKDAGGGSIVNiSsAAGLMGLALTSSyGASkWGVRGLSKLAAVELGTDRIRVNSVHpGmtYtPmtAETGIRQGEGNYPNTPMGRVGEPGEIAGAVVKLLSDTSSYVTGAELAVDGGWTTGPTVKYVMGQ

>2hun_A

SMKLLVTgGMgfiGSNFIRYILEKHPDWEVINIdkLGygsNPANLKDLEDDPRYTFVKGdvADYELVKELVRKVDGVVHlaaesHVDRSISSPEIFLHsNVIGTYTLLESIRRENPEVRFVHvStdEVYGDILKGSFTENDRLMPSSPySATkAASDMLVLGWTRTYNLNASITRcTnnYGPYqfPEkLIPKTIIRASLGLKIPIYGTVRDWLYVEDHVRAIELVLLKGESREIYNISAGEEKTNLEVVKIILRLMGKGEELIELVEDRPGHDLRYSLDSWKITRDLKWRPKYTFDEGIKKTIDWYLKNEWWWKPLVDERILHPTPWKL

>2i2f_A

MKYMITSKGDEKSDLLRLNMIAGFGEYDMEYDDVEPEIVISIGGngTFlSAFHQYEERLDEIAFIGIHTGHlGfyADWRPAEADKLVKLLAKGEYQKVSYPLLKTTVKYGKKEATYLALneSTVkSSGGPFVVDVVINDIHFERFRGDGLCMSTPSGTtayNKsLGGALMHPSIEAMQLTEMASINNRVYRTIGSPLVFPKHHVVSLQPVNDKDFQISVdhLSILHRDVQEIRYEVSAKKIHFARFRSFPFWRRVHDSfIED

>2i65_B

RWRQTWSGPGTTKRFPETVLARGVKYTEIHPEMRHVDGQSVWDAFKGAFISKHPHDITEEDYQPLMKLGTQTVPINKILlwsrIkDLAHQFTQVQRDMFTleDTLLGYLAddLTWJGEFDTSKINYQSJPDwRKDHSNNPVSVFwKTVsRRfAEAAIDVVHVMLDGSrSKIFDKDstfGSVqVHNLQPEKVQTLEAWVIHGGREDSRDLKQDPTIKELESIISKRNIQFSKKNIYRPDKFLQLVKNPEDSSL

>2i9p_B

MPVGFigLgnmGNPMAKNLMKHGYPLIIydvfPDaCKEFQDAGEQVVSSpADVAEKADRIITmlpTSInAIeaYSGANGILKKVKKGSLLIDSStIDPAVSKELAKEVEKMGAVFMDAPvsGgvGAARSGNLTFMVGGVEDEFAAAQELLGCMGSNVVYCGAVGTGQAAkICNNMLLAISMIGTAEAMNLGIRLGLDPKLLAKILNMSSGRCWSSDTYNPVPGVMDGVPSANNYQGGfGTTlMAkdLGLAQDSATSTKSPILLGSLAHQIYRMMCAKGYSKKDFSSVFQFLREEET

>2ixa_A

KKVRIAFiaVglrGQTHVENMARRDDVEIVAFadpdPYmVGRAQEILKKNGKKPAKVFGNGnDDyKNMLKDKNIDAVFVSSpwEwhHEhGVAAMKAGKIVGMevSGAITLEECWDYVKVSEQTGVPLMALEnVCYRRDVMAILNMVRKGMFGELVHGTGGyQHDLRPVLFNSGINGKNGDGVEFGEKAFseAkwrTNHYKNRNGELyPThGVGPLHTMMDINRGNRLLRLSSFASKARGLHKYIVDKGGESHPNAKVEWKQGDIVTTQIQCHNGETIVLTHDTSLQRPYNLGFKVQGTEGLWEDFGWGEAAQGFIYFEKIMNHSHRWDSSEKWIKEYDHPMWKKHEQKAVGAGHGGMDYFLDNTFVECIKRNEAFPLDVYDLATWYSITPLSEKSIAENGAVQEIPDFTNGKWKNAKNTFAINDDY

>2npx_A

MKVIVLGSSHGGYEAVEELLNLHPDAEIQWYEKGDFISFLSCGMQLYLEGKVKDVNSVRYMTGEKMESRGVNVFSNTEITAIQPKEHQVTVKDLVSGEERVENYDKLIISPGAVPfElDIPGKDLDNIYLMrGRQWAIKLKQKTVDPEVNNVVVigsgyiGIeAAEAFAKAGKKVTVidilDRPlGvyLDKEFTDVLTEEMEANNITIATGEtvERYEGDGRVQKVVTDKNAYDADLVVVavgvRPNTAWLKGTLELHPNGLIKTDEYMRTSEPDVFAVGDATLIKYNPADTEVNIalATNARKQGRFAVKNLEEPVKPFPGVQGSsGlAVFDYKFASTGINEVMAQKLGKETKAVTVVEDYLMDFNPDKQKAWFKLVYDPETTQILGAQLMSKADLTANINAISLAIQAKMTIEDLAYADFFFQPAFDKPWNIINTAALEAVKQER

>2nsy_A

SMQEKIMRELHVKPSIDPKQEIEDRVNFLKQyVKKtGAKGFVLGISGGQDSTLAGRLAQLAVESIREEGGDAQFIAVRLPHGTQQDEDDAQLALKFIKPDKSWKFDIKSTVSAFSDQYQQETGDQLTDfNKGnVKarTRMIAQyAIGgQEGLlvLGTDHAAeAVTGfFtkYGdGGAdLLPLTGLTKRQGRTLLKELGAPERLYLKEPTaDlLDEKPQQSdETeLGISYDEIDDYLEGKEVSAKVSEALEKRYSMTEhkRQVPASMFDDwWK

>2o2s_A

PIDLRGQTAFVAgvadSHgygWAIAKHLASAGARVALGTwPPvLGLFQKSLQSGRLDEDRKLPDGSLIEFAGVYPldaaFDKPEDVPQDIKDNKRYAGVDGYTIKEVAVKVKQDLGNIDILVhslanGPEVTKPLLETSRKGYLAASSnSAYSFVSLLQHFGPIMNEGGSAVTlSyLAAERVVPGYGGGMSSAkAALESDTRTLAWEAGQKYGVRVNAISaGplKsraASAIGKSGEKSFiDYAIDYSYNNAPLRRDLHSDDVGGAALFLLSPLARAVSGVTLYVDNGLHAMGQAVDSRSMPP

>2o2z_A

GXKKKNVIVFGGGtgLSvLLRGLKTFPVSITAIVTVADDGGSSGRLRKELDIPPPGDVRNVLVALSEVEPLLEQLFQHRFENGGLSGHSLGNLLLAGXTSITGDFARGISEXSKVLNVRGKVLPASNRSIILHGEXEDGTIVTGESSIPKAGKKIKRVFLTPKDTKPLREGLEAIRKADVIVIGpgsLYTSVLPNLLVPGICEAIKQSTARKVYICnvXTqNGETDGYTASDHLQAIXDHCGVGIVDDILVHGEPISDTVKAkyAKeKAEPVIVDEHKLKALGVGTISDYFVLEQvLrhNASKVSEAILE

>2o4c_A

MRILADENIPVVDAFFADQGSIRRLPGRAIDRAALAEVDVLLVRSVTEVSRAALAGSPVRFVGTATigTDHLDLDYFAEAGIAWSSAPgAnaRGvVDYVLGCLLAMAEVRGADLAERTYGVvgAgqvGGRLVEVLRGLGWKVLVcdpprQArEPDGEFVSlERLLAEADVISLhtpLnRDGEhPtRHlLDEPRLAALRPGTWLVNAsrGAVVDNQALRRLLEGGADLEVALdvWEGEPQADPELAARCLIATPhIagySLEGKLRGTAQIYQAYCAWRGIAERVSLQDVLPETWLAGLQLNPGCDPAWALATLCRAVYDPRSDDAAFRRSLTGDSATRRAAFDALRKHYPPRREITGLRVATGGQAELQRVVRALGAQLV

>2p5u_A

MRVLVTgGAgfiGSHIVEDLLARGLEVAVldnLatgKRENVPKGVPFFRVdlrDKEGVERAFREFRPTHVSHqaaqaSVKVSVEDPVLDFEvNLLGGLNLLEACRQYGVEKLVfaStGGAIYGEVPEGERAEETWPPRPKSPyAASkAAFEHYLSVYGQSYGLKWVSLRyGnvYGPRQdPHGEaGVVAIFAERVLKGLPVTLYARKTPGDEGCVRDYVYVGDVAEAHALALFSLEGIYNVGTGEGHTTREVLMAVAEAAGKAPEVQPAPPRPGDLERSVLSPLKLMAHGWRPKVGFQEGIRLTVDHFRGAV

>2pd3_A

GFLKGKKGLIvgVanNKsiAYGIAQSCFNQGATLAFTYlNESlEKRVRPIAQELNSPYVYEldvsKEEHFKSLYNSVKKDLGSLDFIVHsvafAPKEALEGSLLETSKSAFNTAMEiSVYSLIELTNTLKPLLNNGASVLtlSyLGSTKYMAHYNVMGLAkAALESAVRYLAVDLGKHHIRVNALSaGpiRtlasSGIADfRMILKWNEINAPLRKNVSLEEVGNAGMYLLSSLSSGVSGEVHFVDAGYHVMGMGAVEEKDNKATLLWDLHKEQ

>2pd6_D

NRLRSALALVTgAgsgiGRAVSVRLAGEGATVAAcdldRAaAQETVRLLNHAAFQAdvSEARAARCLLEQVQACFSRPPSVVVScagiTQDEFLLHMSEDDWDKVIAvNLKGTFLVTQAAAQALVSNGCRGSIINiSsIVGKVGNVGQTNyAASkAGVIGLTQTAARELGRHGIRCNSVLpGfiAtpmtQKVPQKVVDKITEMIPMGHLGDPEDVADVVAFLASEDSGYITGTSVEVTGGLF

>2ph5_A

NTKKILFKNRFVIlgFgcvGQALXPLIFEKFDIKPSQVTIIaaeGTKVDVAQQYGVSFKLQqiTPQNYLEVIGSTLEENDFLIDvsigiSSLALIILCNQKGALYINaaTePWkERRTNYSLREEVLRLKDKTQKTALITHganpGLVSHFIKEALLNIAKDNGLTINRPKNAAEWANLAXTLGIKVIHVAEQDSQVTYPPKSPGEFVNTwsANGLILeGLQPAEIGWGTHEAHWPHDAYSHSNGPQCAIYLSRPSAGVXVRSWTPTLGAFHGFLIThAETISLTNFLTLKNGSELLYRPTVHYAYNPCPDARLSIFELKSNEWKPQNKNRLILNEIIDGCDELGVLLXGNQRGAYWYGSTLSIQEARQIAPYNNATSLQvVASXISGIIWAIEHPDEGIVEPEEVDHQYIIDIAKPYLGKVGGYYTDWTPLKNRGELYPEEVDLSDPWQFFNIRVNLE

>2pi1_A

XNVLFTSVPQEDVPFYQEALKDLSLKIYTTDVSKVPENELKKAELISVFVYDKLTEELLSKXPRLKLIHTRSvgFDHIDLDYCKKKGILVTHIPAySPESvAEHTFAXILTLVKRLKRIEDRVKKLNFSQDSEILARELNRLTLGVIgtgriGSRVAXYGLAFGXKVLCydvvKREDLKEKGCVYTSLDELLKESDVISLHvpYtKetHHXINEERISLXKDGVYLINTarGKVVDTDALYRAYQRGKFSGLGLdvFEDEEILILKKYTEGKATDKNLKILELACKDNVIITPhIayYTDKSLERIREETVKVVKAFVKGDLEQIKGNFVVGPS

>2pv7_B

GFKTINSDIHKIVIvgGygklGGLFARYLRASGYPISILdrEDwAVAESILANADVVIVsvpiNlTLEtIErLKPYLTENXLLADLtsVKREPLAKXLEVHTGAVLGLhPXFgADIASXAKQVVVRCDGRFPERYEWLLEQIQIWGAKIYQTNATEHDHNXTYIQALRHFSTFANGLHLSKQPINLANLLALSSPIYRLELAXIGRLFAqdAElYADIIXDKSENLAVIETLKQTYDEALTFFENNDRQGFIDAFHKVRDWFGDYSEQFLKESRQLLQQy

>2pzm_A

HMRILITgGAgclGSNLIEHWLPQGHEILVIdnFatgKREVLPPVAGLSVIEGsvtDAGLLERAFDSFKPTHVVHsaaayKDPDDWAeDAAtNVQGSINVAKAASKAGVKRLLNfQtALCYGRPATVPIPIDSPTAPFTSyGISkTAGEAFLMMSDVPVVSLRlAnvTGPRlAIGPIPTFYKRLKAGQKCFCSDTVRDFLDMSDFLAIADLSLQEGRPTGVFNVSTGEGHSIKEVFDVVLDYVGATLAEPVPVVAPGADDVPSVVLDPSKTETEFGWKAKVDFKDTITGQLAWYDKYGVTDIFSHLSAPK

>2q1u_A

NASKLANTNVMVvgGAgfvGSNLVKRLLELGVNQVHVVdnllsaEKINVPDHPAVRFSETsiTDDALLASLQDEYDYVFHlatYhGNQSSIHDPLADHEnNTLtTLKLYERLKHFKRLKKVVYsAaGEETDIVSLHNNDSPySMSkIFGEFYSVYYHKQHQLPTVRARfqnvYGPGEILGAGRWRGTPATVWrnVTPTFIYKALKGMPLPLENGGVATRDFIFVEDVANGLIACAADGTPGGVYNIASGKETSIADLATKINEITGNNTELDRLPKRPWDNSGKRFGSPEKARRELGFSADVSIDDGLRKTIEWTKANLAVIEQIMRKHDSALATY

>2q2q_D

TLKGKTALVTgStsgiGLGIAQVLARAGANIVLngfGDPAPALAEIARHGVKAVHHPAdlSDVAQIEALFALAEREFGGVDILVNnagiQHVAPVEQFPLESWDkIIAlNLSAVFHGTRLALPGMRARNWGRIINiAsvhGLVGSTGKAAyVAAkHGVVGLTKVVGLETATSNVTCNAICpGwvLtplvQKQIDDRAANGGDPLQAQHDLLAEKQPSLAFVTPEHLGELVLFLCSEAGSQVRGAAWNVDGGWLAQ

>2qg4_B

MFEIKKICCigAgyvGGPTCSVIAHMCPEIRVTVVdvnESrINAWNSPTLPIYEPGLKEVVESCRGKNLFFSTNiDDAIKEADLVFIsvntPTKTYGMGKGRAADLKyIEacARrIVQNSNGYKIVTEKstVPVRAAESIRRIFDANTKPNLNLQVLSNPeFlAeGTAIKDLKNPDRVLIGGDETPEGQRAVQALCAVYEHWVPREKILTTNTWSSELSKLAANAFLAQRISSINSISALCEATGADVEEVATAIGMDQRIGNKFLKASVGFGGscFQkDVLNLVYLCEALNLPEVARYWQQVIDMNDYQRRRFASRIIDSLFNTVTDKKIAILGFAFKKDTGDTrESSSIYISKYLMDEGAHLHIYDPKVPREQIVVDLSHDDQVSRLVTISKDPYEACDGAHAVVICTEWDMFKELDYERIHKKMLKPAFIFDGRRVLDGLHNELQTIGFQIETIGKKV

>2qjo_B

KYQYGIyigrfQPFhLghLRtLNLALEKAEQVIIILgsHRVAADTrNPWRSPERMAMIEACLSPQILKRVHFLTVRdWlYSdNLwLAAVQQQVLKITGGSNSVVVLghRkDAssyylNLFPQWDYLEtGhyPDfSsTAIRGAYFEGKEGDYLDKVPPAIADYLQTFQKSERYIALCDEYQFLQAYKQAWATAPYAPTFITTDAVVVQAGHVLMVRRQAKPGLGLIALPGGFIKQNETLVEGMLRELKEETRLKVPLPVLRGSIVDSHVFDAPGRSLRGRTITHAYFIQLPGGELPAVKGGDDAQKAWWMSLADLYAQEEQIYEDHFQIIQHFVSKV

>2rc3_C

HMKTVKHLLQEkGHtvvAIGPDDSVFNAMQKMAADNigaLlVMKDEKLVGILTErDFSRKSYLLDKPVKDTQVKEIMTRQVAYVDLNNTNEDCMALiteMrVrhLPVLDDGKVIGlLsigdLvkDAIS

>2vhx_E

MRVGIPTETKNNEFRVAITPAGVAELTRRGHEVLIQAGAGEGSAITDADFKAAGAQLVGTADQVWADADLLLKVKEPIAAEYGRLRHGQILFTFLhLAASRACTDALLDSGTTSIAYeTVQTADGAlPLlaPmsEVaGRLAAQVGAYHLMRTQGGRGVLMGGVPGVEPADVVVigAgtaGYNAARIANGMGATVTVLdinIDkLRQLDAEFCGRIHTRYsSAYElEGAVKRADLVIGAvlvpGAKApKlVSNSLVAHMKPGAVLVDiaidqGGCFEGSRPTTYDHPTFAVHDTLFYCVaNmpASVPKTSTYALTNATMPYVLELADHGWRAACRSNPALAKGLSTHEGALLSERVATDLGVPFTEPASVLAHH

>3b4w_A

SATEYDKLFIGGKWTKPSTSDVIEVRCPATGEYVGKVPMAAAADVDAAVAAARAAFDNGPWPSTPPHERAAVIAAAVKMLAERKDLFTKLLAAETGQPPTIIETMHWMGSMGAMNYFAGAADKVTWTETRTGSYGQSIVSREPVGVVGAiVawnVPLFlAVNKIAPALLAGCTIVLkPaAETPLTANALAEVFAEVGLPEGVLSVVPGgiETgqALtSNPDIDMFTftgsSAvGRevGRRAAEMLKPCTLeLgGKSAAIILEDVDLAAAIPMMVFSGVMNAGQGcVNQTRILAPRSRYDEIVAAVTNFVTALPVGPPSDPAAQIGPLISEKqRTRVEGYIAKGIEEGARLVCGGGRPEGLDNGFFIQPTVFADVDNKMTIAQEeIfGPVLAIIPYDTEEDAIAIANDSVYGlAGSVWTTDVPKGIKISQQIRTGTYGINWYAFDPGSPfGGYKNSGIGReNGPEGVEHFTQQKSVLLPMGYTV

>3b6j_A

AKVLVLYYSmyGhIETMARAVAEGASKVDGAEVVVKRVPEtmpPQlFEKagGKTQTAPVATPQELADYDAIIFGTPTrFGNMSgqMRTFLDQTGGLWASGALYGKLASVFSSTGTGGGQEQTITSTWTTLAHHGMVIVPIGYAAQELFDVSQVRGGTPYGATTIAGGdgSRQPSQEELSIARYQGEYVAGLAVKLNG

>3bts_B

AAPIRVGFvgLnAAkgwAIKTHYPAILQLSSQFQITALyspKIEtSIATIQRLKLSNATAFPTLESFASSSTIDMIVIaiqvAshYevVMPLLEFSKNNPNLKYLFVeWALACSLDQAESIYKAAAERGVQTIIsLqGRKSPYILRAKELISQGYIGDINSIEIAGNGGWYGYERPVKSPKyIYEIGNGvDLVTtTFGhTIDILQYMTSSYFSRINAMVFNNIPEQELIDERGNRLGQRVPKTVPDHLLFQGTLLNGNVPVSCSFKGGKNLVIDIHGTKRDLKLEGDAaEISNLVLYYSDAGKEIMEVYHLRNYNAIVGNIHRLYQSISDFHFNTKKIPELPSQFVMQGFDFEGFPTLMDALILHRLIESVYKSNMMGSTLNVSNISHYS

>3cea_A

TRKPLRAAIigLgrlGERHARHLVNKIQGVKLVAAcaldSNqLEWAKNELGVETTYTNyKDXIDTENIDAIFIvaptPfhPEXTIYAXNAGLNVFCekpLGLDFNEVDEXAKVIKSHPNQIFQSGFXRRYDDSYRYAKKIVDNGDIGKIIYXRGYGIDPISGXESFTkfATeADSGGIFVdXNIhDIDLIRWFTGQDPVQAYGLTSNIAAPQLADIGEFETGVAQLKXSDGVIATLIGGRHAAHGNQVELEVXGSNGWVRIGEHPDLNRVTVFNDQGVVRPSLQSfGERFDTAfTDEVQDFVNNVIVGKQPEVTVDDGIKALKIAKACQQSANIGKLVDIQL

>3cin_A

HMVKVLILgQgyvASTFVAGLEKLRKGEIEPYGVPLARELPIGFEDIKIVGSydvdRAkIGKKLSEVVKQyWNDVDSLTSDPEIRKGVhLGsvRNLPiEAEGLEDSMTLKEAVDTLVKEWTELDPDVIVNtcttEAFVPFGNKEDLLKAIENNDKERLTaTQVyAYAAALYANKRGGAAFVNvipTFIANDPAFVELAKENNLVVFGDdgAtGATPFTADVLSHLAQRNRYVKDVAQFNIGGnmdfLALTDDGKNKSKEFTKSSIVKDILGYDAPHYIKPTGYLEPLGDKkFIAIHIEYVSFNGATDELMINGRINdsPAlGGLLVDLVRLGKIALDRKEFGTVYPVNAFYMkNPGPAEEKNIPRIIAYEKMRIWAGLKPKW

>4mdh_A

SEPIRVLVtgAagqiAYSLLYSIGNGSVFGKDQPIILVLldiTPmMGVLDGVLMELQDCALPLLKDVIATDKEEIAFKDLDVAILvgsMprRDGMERKDLlKANVKiFKCqGAALDKYAKKSVKVIVvgnPaNTNCLTASKSAPSIPKENFSClTRlDHNRAKAQIALKLGVTSDDVKNVIIWGNhSSTQYPDVNHAKVKLQAKEVGVYEAVKDDSWLKGEFITTVQQRGAAVIKARKLssAMSaAKAICDHVRDIWFGTPEGEFVSMGIISDGNSYGVPDDLLYSFPVTIKDKTWKIVEGLPINDFSREKMDLTAKELAEEKETAFEFLSSA

>2a9k_B

TYQEFTNIDQAKAWGNAQYKKYGLSKSEKEAIVSytKSasEInGKLrQNKGVINGFPSNLIKQVELLDKSFNKMKTPENIMLFrGddPayLGTEFQNTLLNSNGTINKTAFEKAKAKFLNKDrLeYGYIsTsLMNVsQfAGrPIITKFKVAKGSKAGYIDPIsAfAGqLeMLLPRHSTYHIDDMRLSSDGKQIIITATMMGTAINPK

>2bkj_B

NNTIETILAHRSIrKFTAVPITDEQRQTIIQAGLAASSSSMLQVVSIVRVTDSEKRNELAQFAgNqAyVESAAEFLVFCIDYQRHATINPDVQADFTELTLIGAVDSGIMAQNCLLAAESMGLGGVYIggLRnSAAQVDELLGLPENSAVLFGMCLGHPDQNPEVKPRLPAHVVVHENQYQELNLDDIQSYDQTMQAYySTWSQEVTGkLAGESrPHILPYLNSKGLAKR

>2c8h_A

NTYQEFTNIDQAKAWGNAQYKKYGLSKSEKEAIVSytKSasEInGKLrQNKGVINGFPSNLIKQVELLDKSFNKMKTPENIMLFrGddPayLGTEFQNTLLNSNGTINKTAFEKAKAKFLNKDrLeYGYIsTsLMNVSAfAGrPIITKFKVAKGSKAGYIDPISAfAGqLeMLLPRHSTYHIDDMRLSSDGKQIIITATMMGTA

>1x14_B

HHHGRIGIPRERLTNETRVAATPKTVEQLLKLGFTVAVESGAGQLASFDDKAFVQAGAEIVEGNSVWQSEIILKVNAPLDDEIALLNPGTTLVSFIWPAQNPELMQKLAERNVTVMAMDSVPrisRAqSLDALsSMaNIAGYRAIVEAAHEFGRFFTGQITAAGKVPPAKVMVigAgvaGLAAIGAANSLGAIVRAfdtrPEVKEQVQSMGAEFLELDMSDAFIKAeMELFAAQAKEVDIIVTtalipGKPApKlITREMVDSMKAGSVIVDLAAQNGGNCEYTVPGEIFTTENGVKVIGYTDLPGRLPTQSSQLYGTNLVNLLKLLCKEKDGNITVDFDDVVIRGVTVIRAGEITWPAPPIQv

>1x31_A

SKPQRLSAAQTAGARINRDEALTLTVDGQQLSAFRGDTVASAMLANGLRSCGNSMYLDRPRGIFSAGVEEPNALITVGARHQADINESMLPATTVSVTDGLNATLLSGLGVLDPSEDPAYYDHVHVHTDVLVvgAgpaGLAAAREASRSGARVMLLderPEAGgtlREASGeQIDGIDAAQWIDAVTEELAAAEETTHLQRTtvFGSYDANYILAAQRRTVHLDGPSGQGVSRERIWHIRAKQVVLAtaahERPIVFENNDRPGIMLAGSVRSYLNRFGVRAGSKIAVATTndsVYpLVSELAASGGVVAVIDARQNISAAAAQAVTDGVTVLTGSVVANTEADASGELSAVLVATLDEQRNLGEAQRFEADVLAVSGGfNPvVHlHSQRQGKLNWDTSIHAFVPADAVANQHLAgaLTGLldtASaLSTGAATGAAAASAAGFEKIAEVPQALAvPAGETRPVWLVPSLSGDDAVHYKFHFVDLQRDQTVADVLRATGAGMQSVEHIKRYTSISTANDQGKTSGVAAIGVIAAVLGIENPAQIGTTTFRAPyTPVSFAALAGRTRGELLDPARLTAMHPWHLAHGAKFEDVGQWKRPWYYPQDGESMDEAVYRECKAVRDSVGMLDASTLGKIEIRGKDAAEFLNRMYTNGYTKLKVGMGRYGVMCKADGMIFDDGVTLRLAEDRFLMHTTTGGAADVLDWLEEWLQTEWPELDVTCTSVTEQLATVAVVGPRSRDVIAKLASSLDVSNDAFKFMAFQDVTLDSGIEARISRISFSGELAFEIAIPAWHGLQVWEDVYAAGQEFNITPYGTETMHVLRAEKGFIIVGQDTDGTVTPQDAGMEWVVSKLKDFVGKRSFSREDNVREDRKHLVSVLPVDSSLRLAEGAALVAADAVASEGVTPMEGWVTHAYNSPALGRTFGLALIKNGRNRIGEVLKTPVDGQLVDVQVSDLVLFDPEGSRRD

>1hzz_A

MKIAIPKERRPGEDRVAISPEVVKKLVGLGFEVIVEQGAGVGASITDDALTAAGATIASTAAQALSQADVVWKVQRPMTAEEGTDEVALIKEGAVLMCHLGALTNRPVVEALTKRKITAYAMELMPrIsRaqSMdIlsSQsNLAGYRAVIDGAYEFARAFPMMMTAAGTVPPARVLVFgVgvAGlQAIATAKRLGAVVMAtdvrAATKEqVESLGGKFITVDAGgyaKEmGEEfRKKqAEAVLKELVKTDIAITtalipGKPApVlITEEMVTKMKPGSVIIDLAVEAGGNCPLSEPGKIVVKHGVKIVGHTNVPSRVAADASPLFAKNLLNFLTPHVDKDTKTLVMKLEDETVSGTCVTRD

>1o5i_B

GIRDKGVLVLaAsrgiGRAVADVLSQEGAEVTIBarnEElLKRSGHRYVVBdlrKDLDLLFEKVKEVDILVLnaggPKAGFFDELTNEDFKEAIDsLFLNMIKIVRNYLPAMKEKGWGRIVAiTsFSVISPIENLYTSNSArMALTGFLKTLSFEVAPYGITVNCVApGWTEterVKELLSEEKKKQVESQIPMRRMAKPEEIASVVAFLCSEKASYLTGQTIVVDGGLSKFPL

>1nvb_A

NPTKISILGRESIIADFGLWRNYVAKDLISDCSSTTYVLVTdTniGSiyTPSFEEAFRKRAAEITPSPRLLIYNRPpgeVSkSRQTKADIEDWMLSQNPPCGRDTVVIALGggviGdLTGFVASTYMRGVRYVQVPttLlaMVdsSIGGktaIDTPLGknLIGAIWQPTKIYIDLEfLEtlpVReFInGMAeVIKTAAISSEEEFTALEENAETILKAVRREVTPGEHRFEGTEEILKARILASARHkAYVVSADEREGGLRNLLNWGHSIGhAIeAILTPQIlhGECVAIGMVKEAELARHLGILKGVAVSRIVKCLAAYGLPTSLKDARIRKLTAGKHCSVDQLMFNMALDKKNDGPKKKIVLLSAIGTPYETRASVVANEDIRVVL

>1ml3_B

MPIKVGIngFgriGRMVFQALCEDGLLGTEIDVVAVVdmnTDAEYFAYQMRYDTVHGKFKYEVTTTKSSPSVAKDDTLVVNGHRILCVKaQrNPADLPWGKLGVEYVIEStglftAKAAAEGHLRGGARKVVIsapASGGAKTLVMGVNHHEYNPSEHHVVSNAscTTNCLAPIVHVLVKEGFGVQTGLMTTIHSYtatQKTvDgvSVKDWRGGRAAAVNIIPSTTGAAKAVGMVIPSTQGKLTGMSFRVPTPDVSVVDLTFTAARDTSIQEIDAALKRASKTYMKGILGYTDEELVSADFINDNRSSIYDSKATLQNNLPKERRFFKIVSWYDneWGySHRVVDLVRHMASKDRSARL

>1s20_G

XKVTFEQLKAAFNRVLISRGVDSETADACAEXFARTTESGvYshGVNRFPRFIQQLENGDIIPDAQPKRITSLGAIEQWDAQRSIGNLTAKKXXDRAIELAADHGIGLVALRNANhwXrGGSYGWQAAEKGYIGICWtNsIAVXPPWGAKECRIGtNpLiVAIPSTPITXvdXsXSXfsYGXLEVNRLAGRQLPVDGGFDDEGNLTKEPGVIEKNRRILPXGYWKGSGXSIVLDXIATLLSDGASVAEVTQDNSDeYGISQIFIAIEVDKLIDGPTRDAKLQRIXDYVTSAERADENQAIrLpgHefTTLLAENRRNGITVDDSVWAKIQALLEH

>1wdk_A

MIYEGKAITVTALESGIVELKFDLKGESVNKFNRLTLNELRQAVDAIKADASVKGVIVSSGKDVFIVGADITEFVENFKLPDAELIAGNLEANKIFSDFEDLNVPTVAAINGIALGGGLEMCLAADFRVMADSAKIGLPEVKLGIYPGFGGTVRLPRLIGVDNAVEWIASGKENRAEDALKVSAVDAVVTADKLGAAALDLIKRAISGELDYKAKRQPKLEKLKLNAIEQMMAFETAKGFVAGQAGPNYPAPVEAIKTIQKAANFGRDKALEVEAAGFAKLAKTSASNCLIGLFLNDQELKKKAKVYDKIAKDVKQAAVlgAgimGGGIAYQSASKGTPILMkdinEHgIEqGLAEAAKLLVGRVDKGRMTPAKMAEVLNGIRPTLSyGDFGNVDLVVeavveNPKvkQAvLAeVENHVREDAILASntsTISISLLAKALKRPENFVGMhFfnPVHMMPLVEVIRGEKSSDLAVATTVAYAKKMGKNPIVVNDCPGFLVNRVLFPYFGGFAKLVSAGVDFVRIDKVMEKFGWPMGPAYLMDVVGIDtGHHGRDVMAEGFPDRMKDDRRSAIDALYEAKRLGQKNGKGFYAYEKKLVDSSVLEVLKPIVYEQRDVTDEDIINWMMIPLCLETVRCLEDGIVETAAEADMGLVYGIGFPLFRGGALRYIDSIGVAEFVALADQYAELGALYHPTAKLREMAKNGQSFFG

| **List of Negative proteins (non-ligand binding) as obtained from PDB and at 40% CDHIT redundancy** | | | | |
| --- | --- | --- | --- | --- |
| 1a36_A  1a3a_C  1a3h_A  1a3k_A  1a3q_A  1aaj_A  1ab4_A  1ac0_A  1acf_A  1ad6_A  1ade_A  1ae9_A  1aja_A  1ajm_A  1ajr_A  1ak1_A  1ako_A  1aly_A  1at0_A  1ath_A  1ati_A  1atz_B  1au7_A  1aud_A  1b0z_A  1b24_A  1b2p_A  1b35_A | 1bam_A  1bbw_A  1bkb_A  1bkh_B  1bkn_A  1bkp_A  1bkr_A  1bkz_A  1bl0_A  1ble_A  1bm0_A  1bm8_A  1ceo_A  1cex_A  1cez_A  1cf7_B  1cfe_A  1cfr_A  1cfy_A  1d1d_A  1d1n_A  1d1u_A  1dyn_A  1dys_A  1dzf_A  1dzl_A  1e0k_A  1e0r_B | 1el6_A  1elk_A  1ewv_A  1ewz_A  1ex6_A  1ey4_A  1eyb_A  1eyg_A  1eyh_A  1f6y_A  1f7c_A  1f7v_A  1f8x_A  1fi4_A  1fje_B  1fjh_A  1fus_A  1fux_A  1fva_B  1fvk_A  1gqe_A  1gqn_A  1gqq_A  1gqz_A  1gri_A  1h99_A  1h9d_A | 1hjr_A  1hka_A  1hkf_A  1hkg_A  1hks_A  1wis_A  1wiw_B  1wix_A  1wj1_A  2be1_A  2ben_A  2bfx_B  2bg5_A  2bgh_A  2bgk_A  2iia_A  2ijq_A  2ijr_A  2ijz_A  2ikb_A  2iks_A  3ep0_A  3ep1_A  3epu_A  1b35_B  1b35_C  1bak_A | 2yuw_A  2yux_A  2yuy_A  2yv4_A  2yva_A  2yvh_B  2yvn_A  2yvq_A  2yvs_A  3d1b_A  3d1n_I  3d21_A  3d26_A  3d2a_A  3d33_A  3d3a_A  3d3c_A  3d3k_A  3eoy_C  3eoy_G  1e15_A  1e17_A  1ekq_B  1hao_H  1har_A  1hbq_A  1h9d_D |
